# Supplementary figures and images for: Fine-tuning FAM161A gene augmentation therapy to restore retinal function (part 1 of 2)
Source: EMBO Mol Med. 2024 Mar 19;16(4):805–22. doi: 10.1038/s44321-024-00053-x (PMC11018783; doi:10.1038/s44321-024-00053-x)

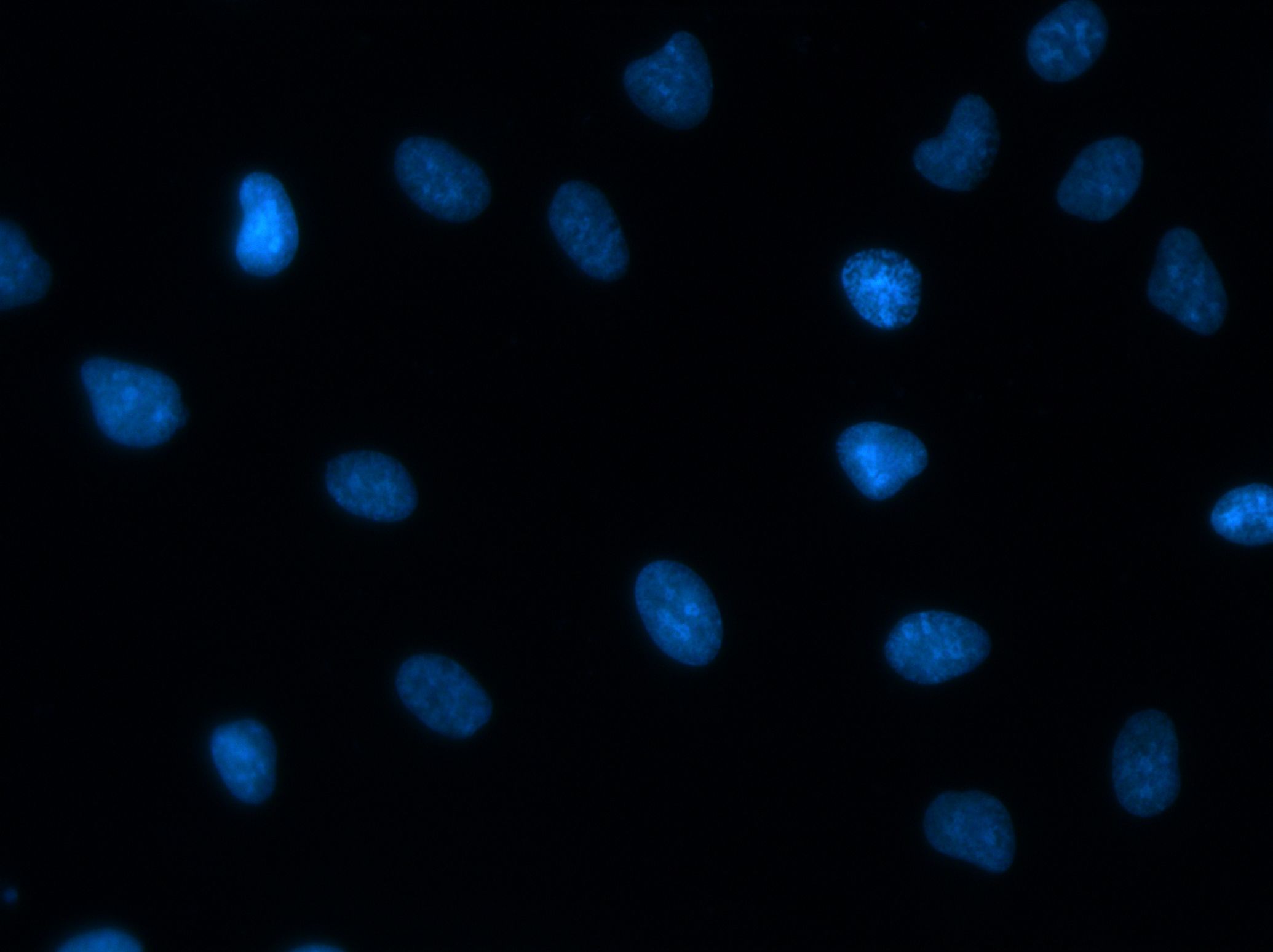

Supplement: Supplementary file 2 — Source Data Fig. 1 [file 44321_2024_53_MOESM2_ESM.zip › Figure 1/1A/ARPE19 dapi.tif]

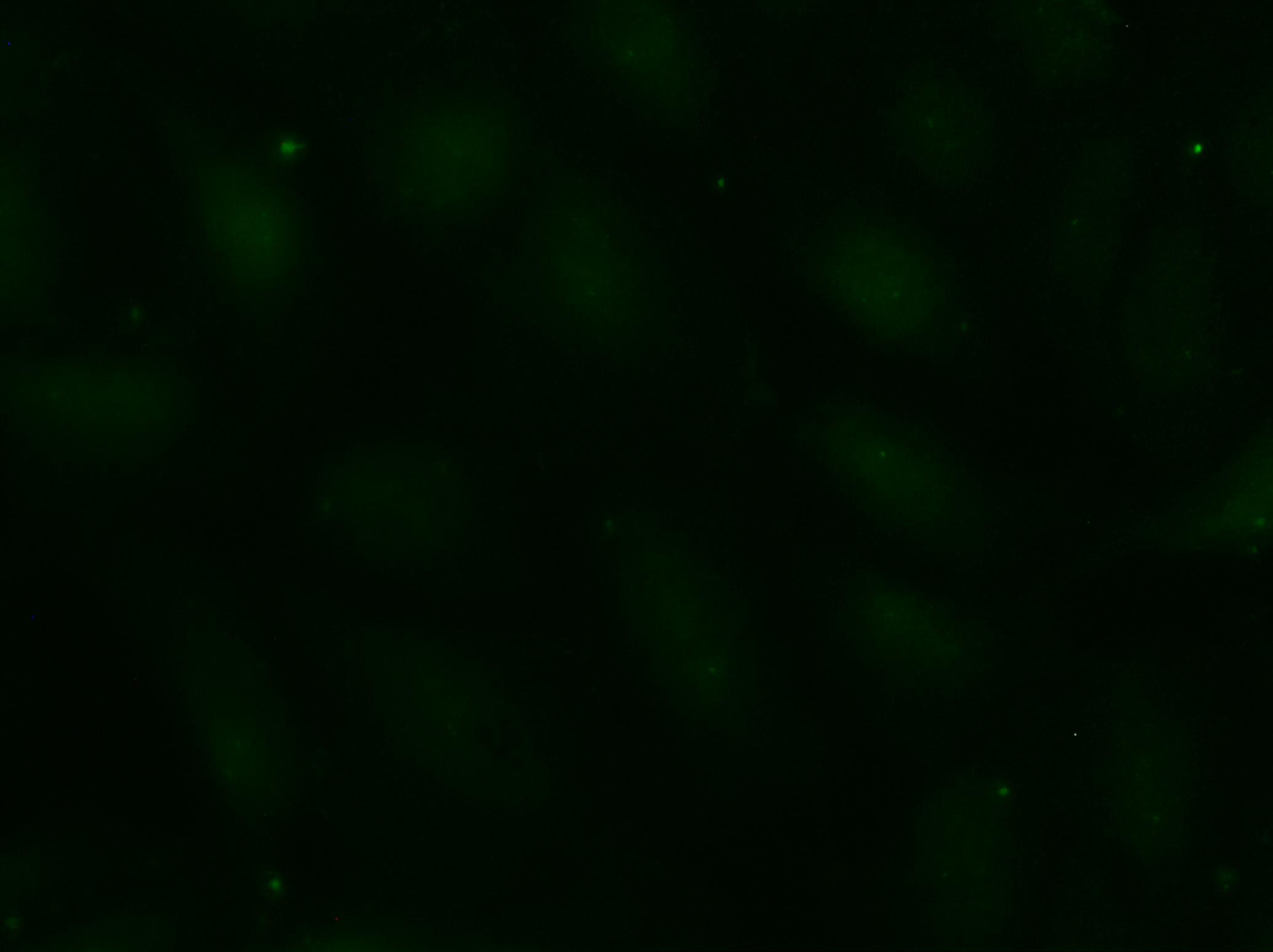

Supplement: Supplementary file 2 — Source Data Fig. 1 [file 44321_2024_53_MOESM2_ESM.zip › Figure 1/1A/ARPE19 FAM161A.tif]

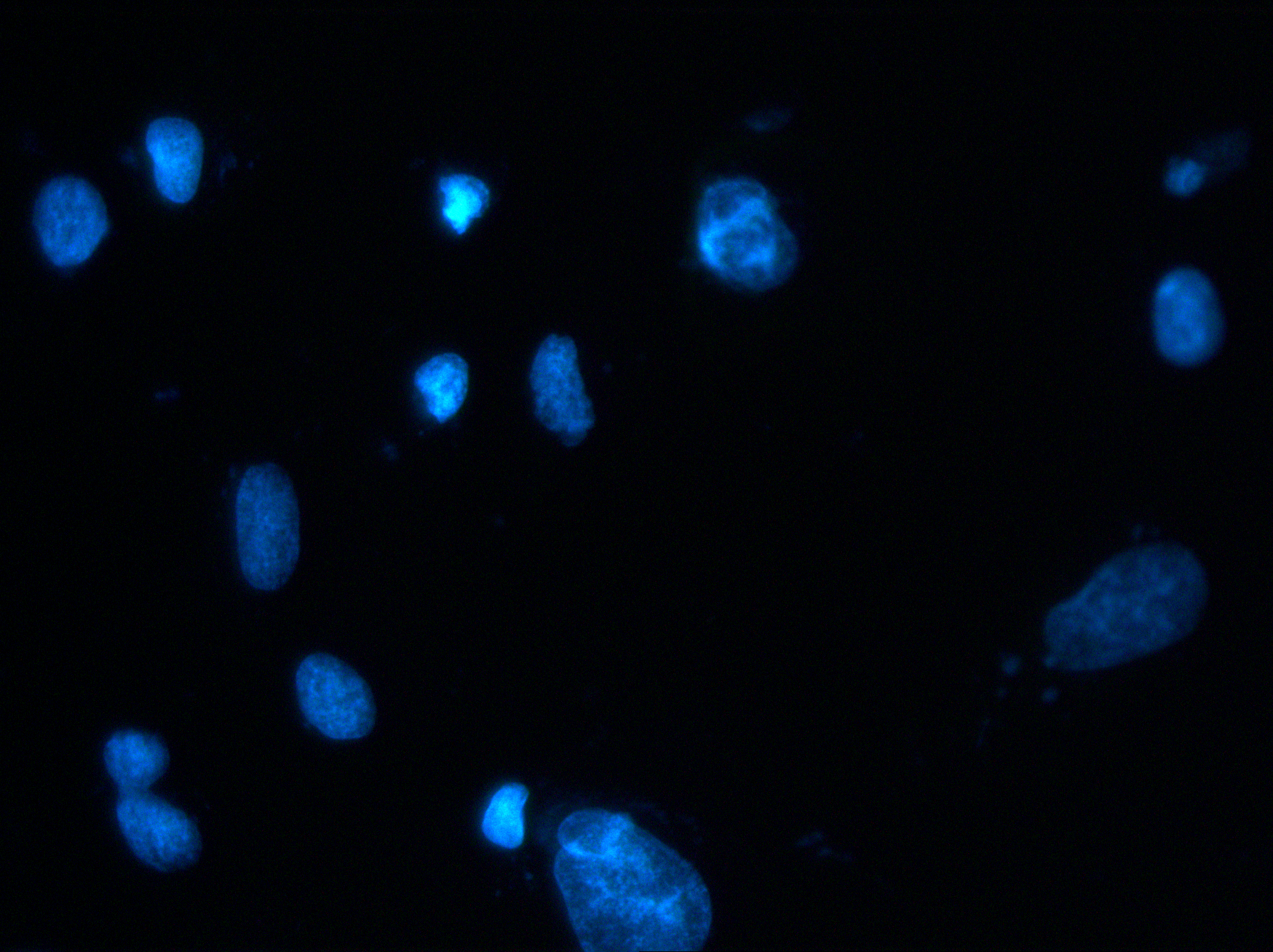

Supplement: Supplementary file 2 — Source Data Fig. 1 [file 44321_2024_53_MOESM2_ESM.zip › Figure 1/1A/ARPE19+HL dapi.tif]

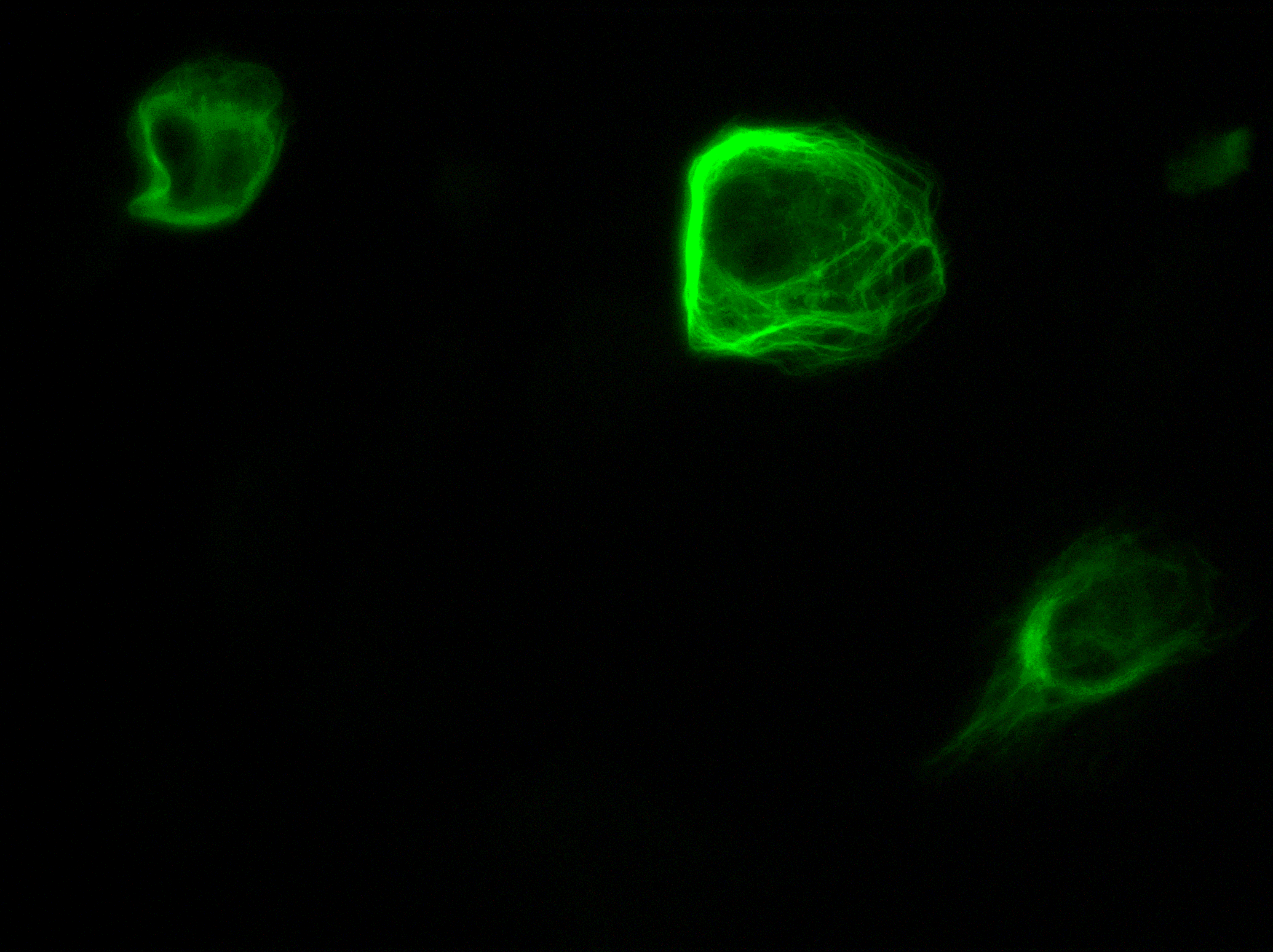

Supplement: Supplementary file 2 — Source Data Fig. 1 [file 44321_2024_53_MOESM2_ESM.zip › Figure 1/1A/ARPE19+HL FAM161A.tif]

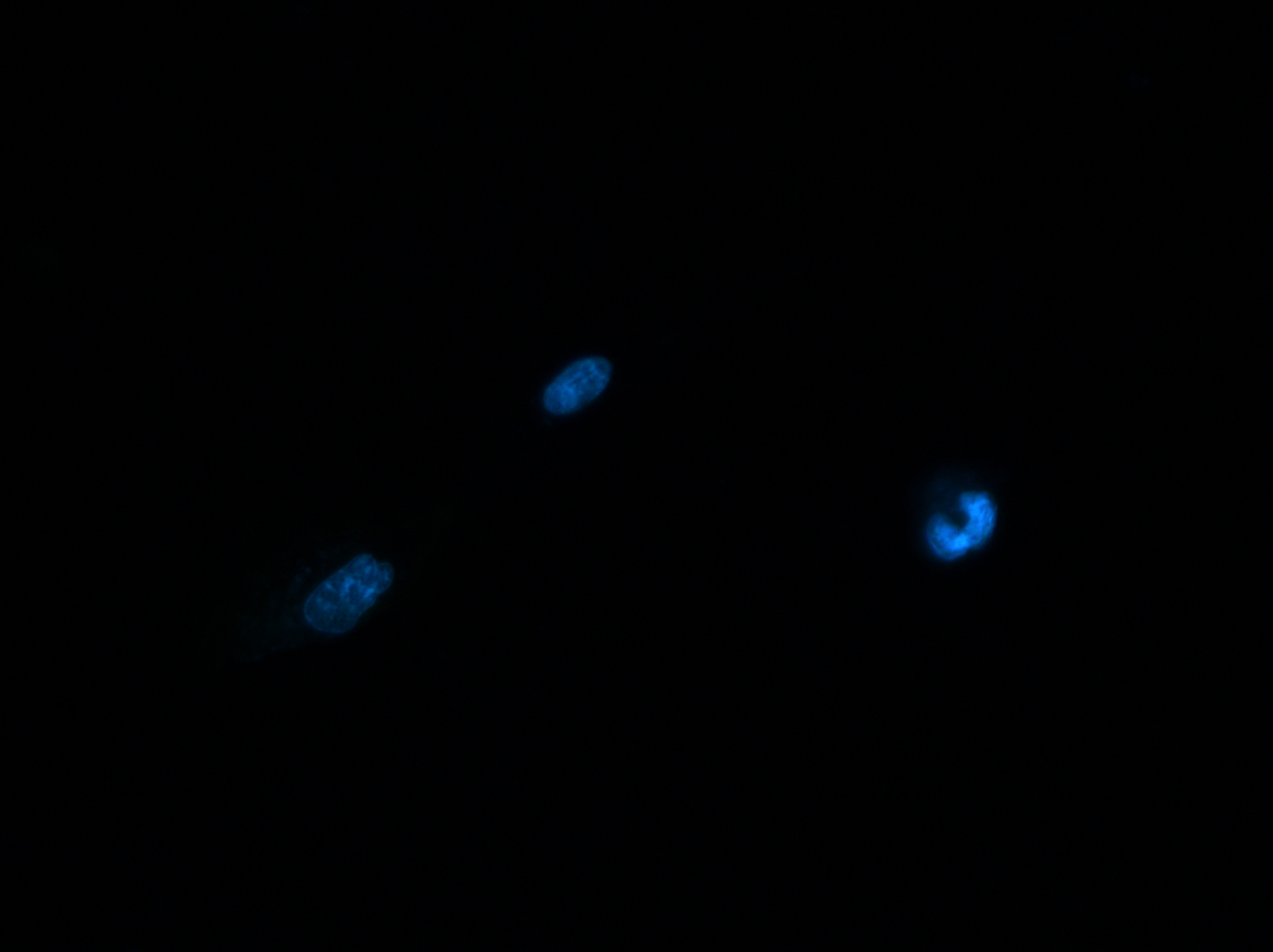

Supplement: Supplementary file 2 — Source Data Fig. 1 [file 44321_2024_53_MOESM2_ESM.zip › Figure 1/1A/ARPE19+HS dapi.tif]

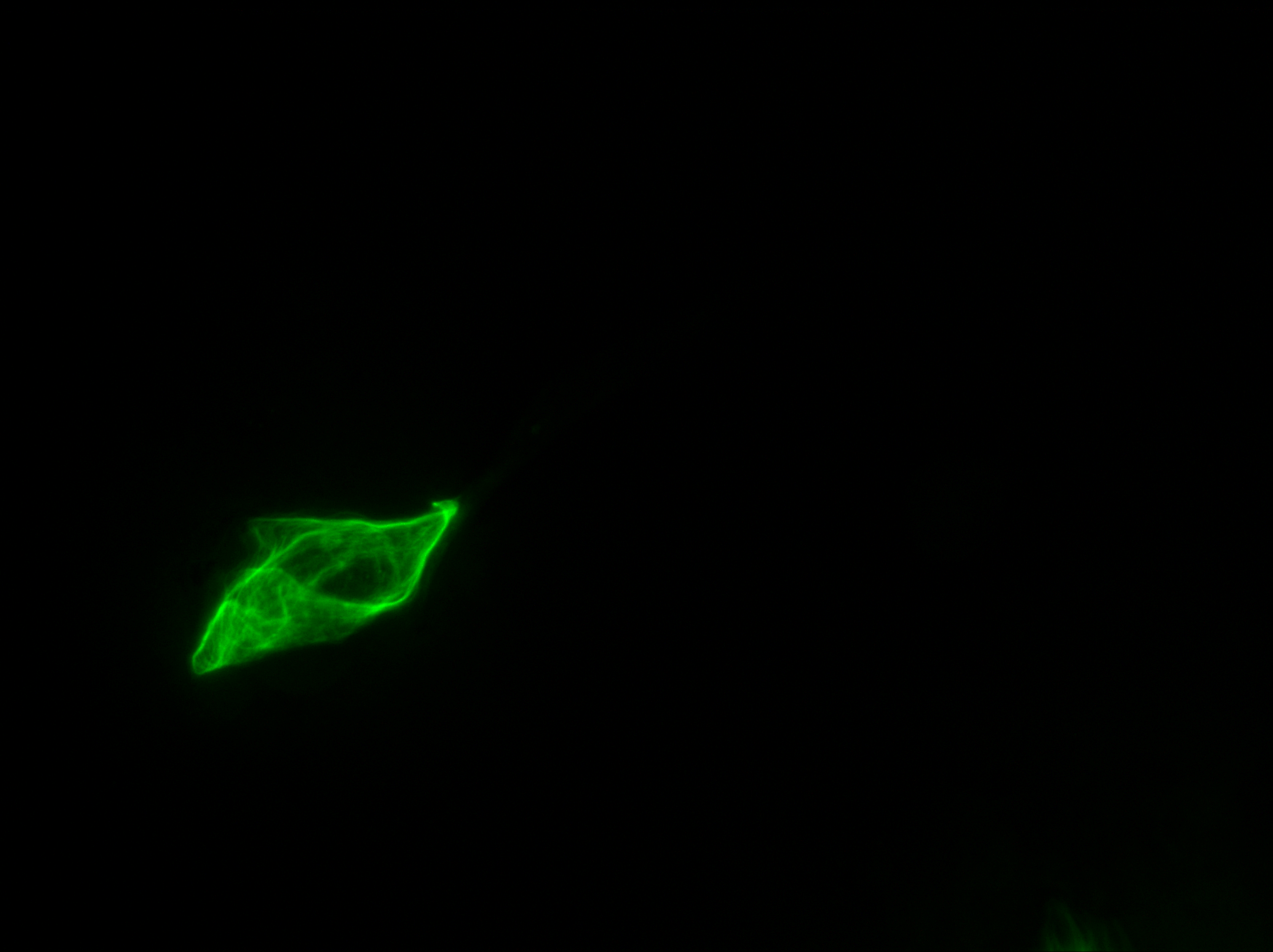

Supplement: Supplementary file 2 — Source Data Fig. 1 [file 44321_2024_53_MOESM2_ESM.zip › Figure 1/1A/ARPE19+HS FAM161A.tif]

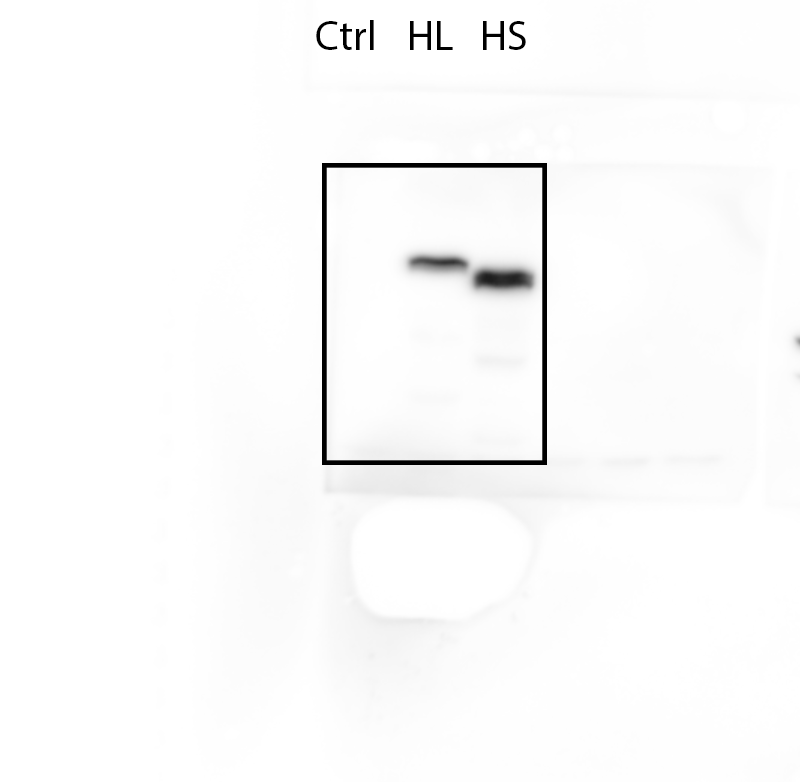

Supplement: Supplementary file 2 — Source Data Fig. 1 [file 44321_2024_53_MOESM2_ESM.zip › Figure 1/1B/Western 661W FAM161A.tif]

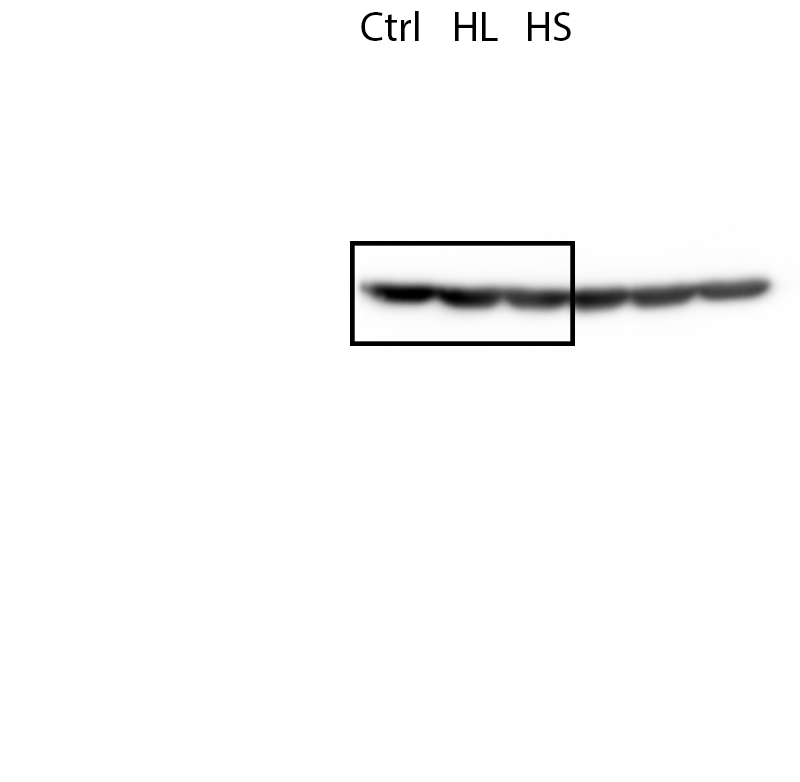

Supplement: Supplementary file 2 — Source Data Fig. 1 [file 44321_2024_53_MOESM2_ESM.zip › Figure 1/1B/Western 661W GADPH.tif]

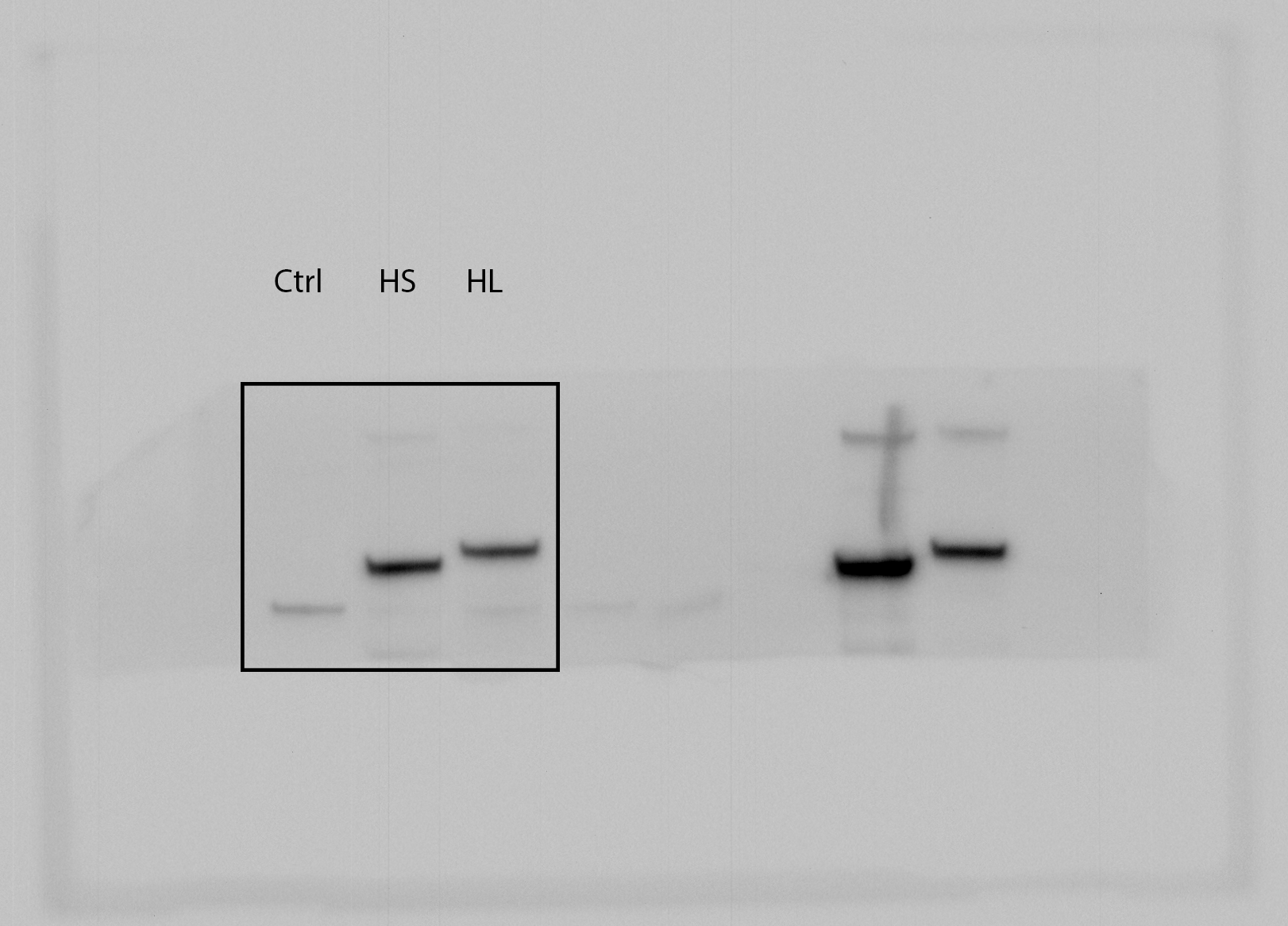

Supplement: Supplementary file 2 — Source Data Fig. 1 [file 44321_2024_53_MOESM2_ESM.zip › Figure 1/1B/Western ARPE19 FAM161A.tif]

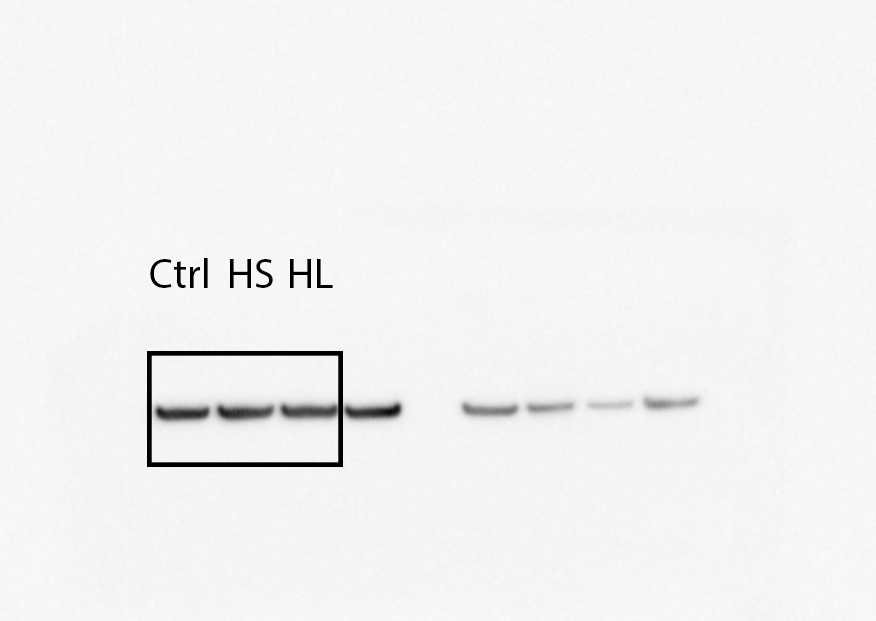

Supplement: Supplementary file 2 — Source Data Fig. 1 [file 44321_2024_53_MOESM2_ESM.zip › Figure 1/1B/Western ARPE19 GAPDH.tif]

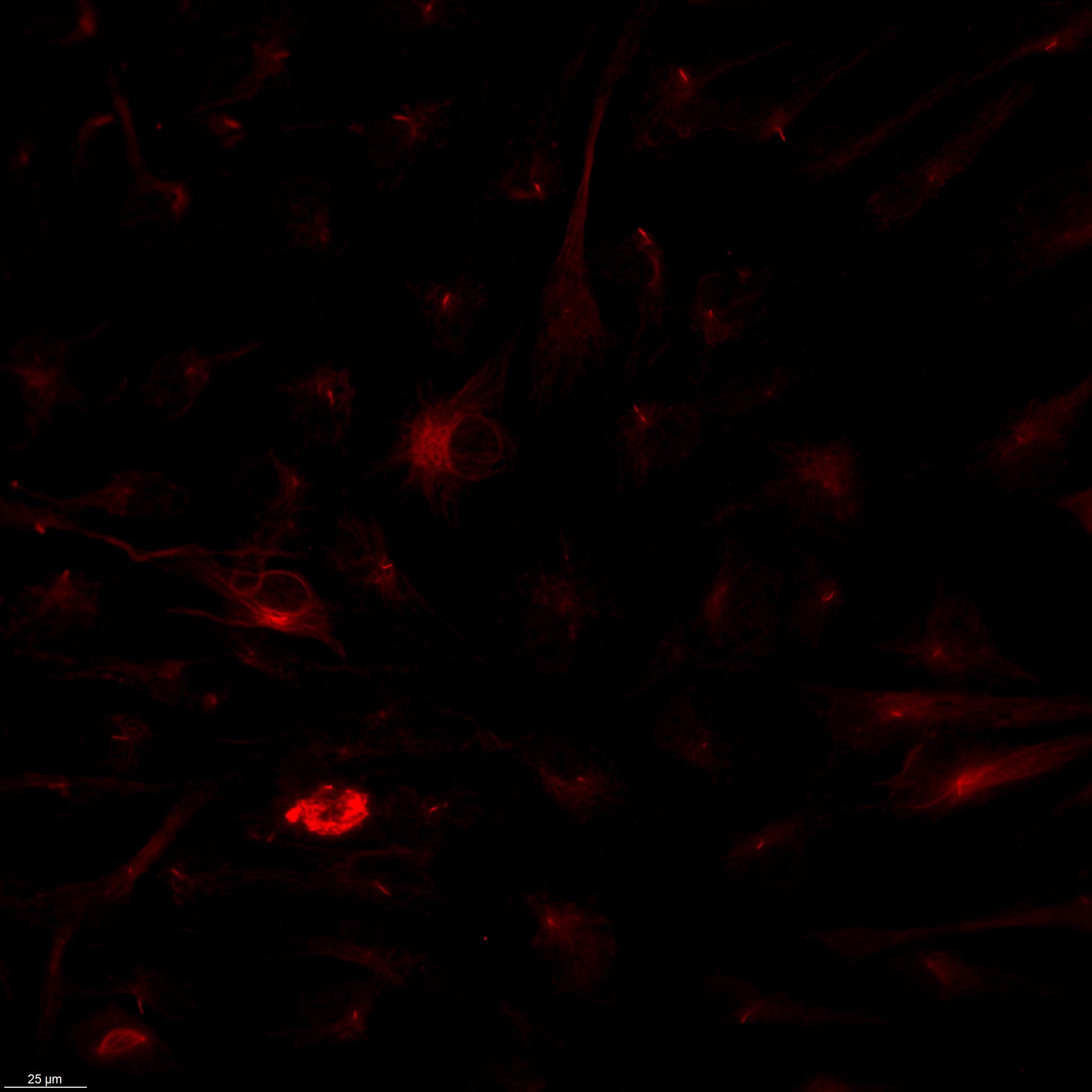

Supplement: Supplementary file 2 — Source Data Fig. 1 [file 44321_2024_53_MOESM2_ESM.zip › Figure 1/1C/HL Ac-TUB.tif]

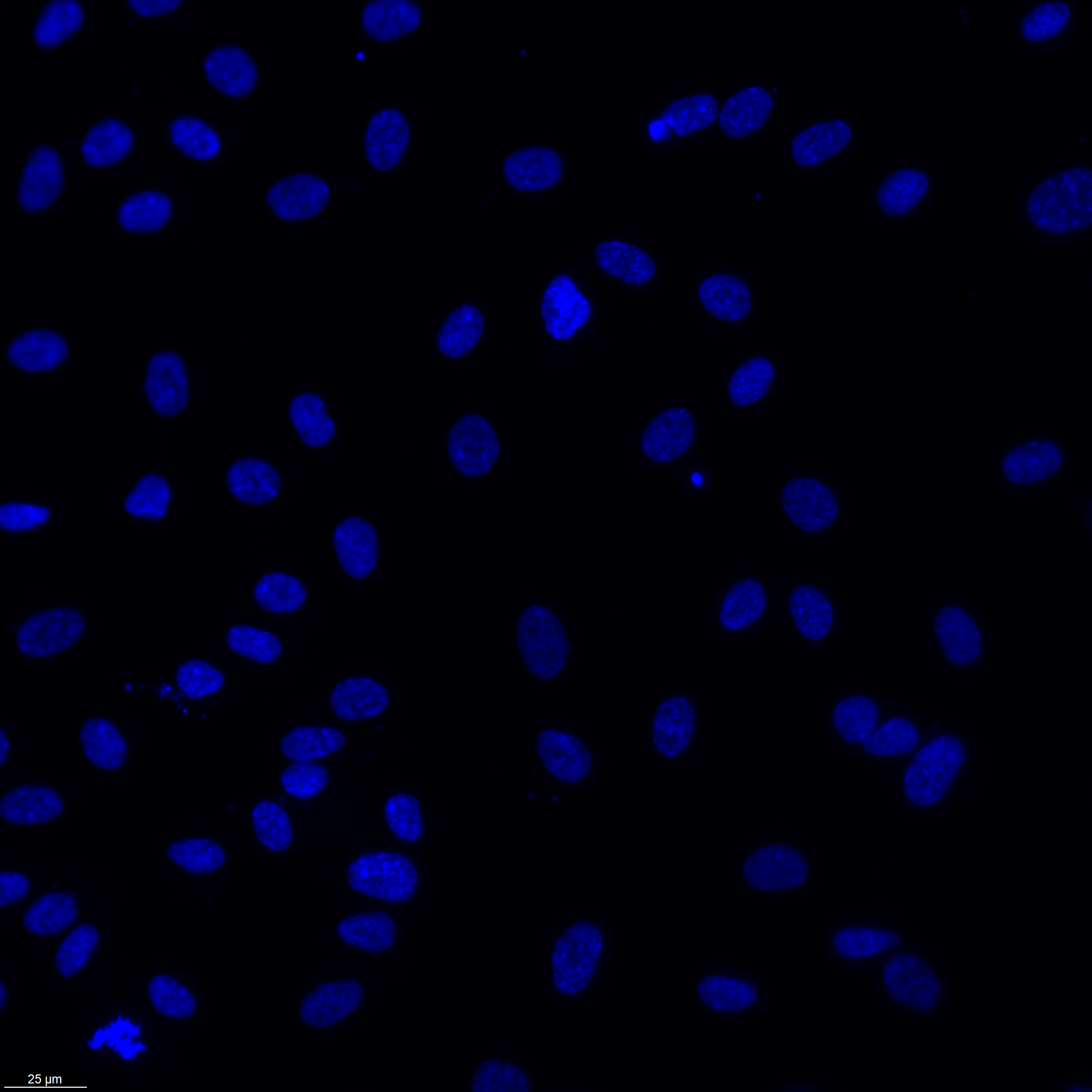

Supplement: Supplementary file 2 — Source Data Fig. 1 [file 44321_2024_53_MOESM2_ESM.zip › Figure 1/1C/HL dapi.tif]

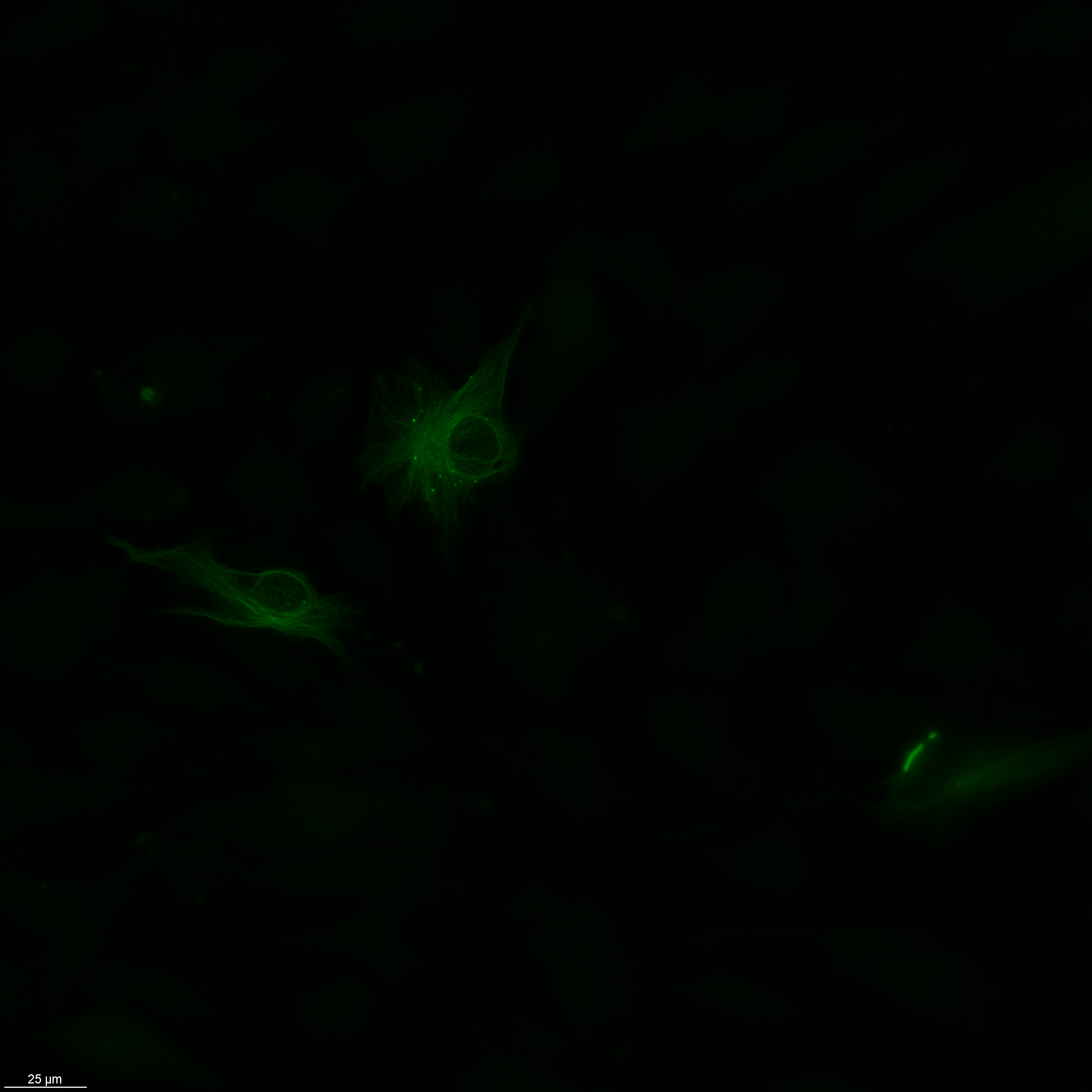

Supplement: Supplementary file 2 — Source Data Fig. 1 [file 44321_2024_53_MOESM2_ESM.zip › Figure 1/1C/HL FAM161A.tif]

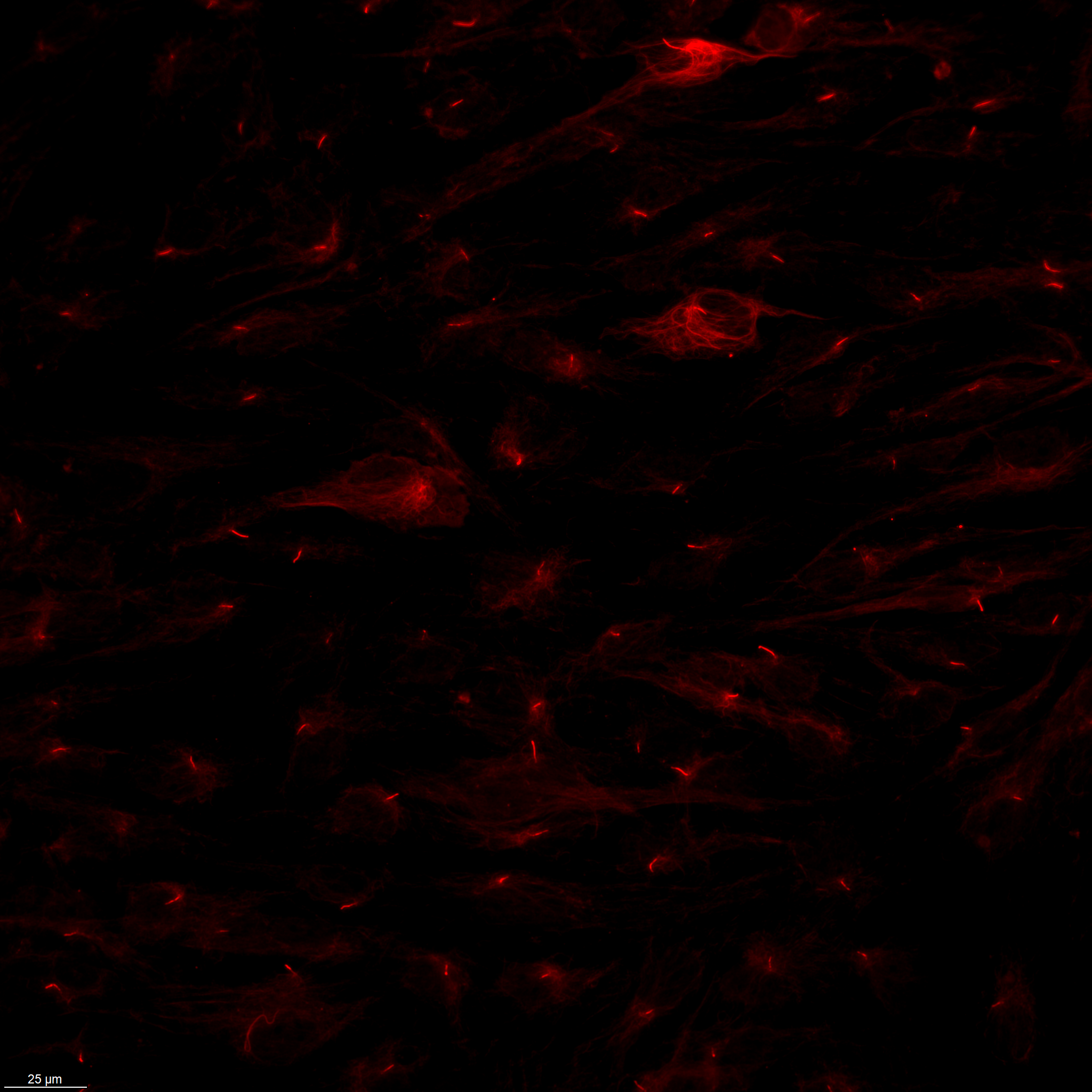

Supplement: Supplementary file 2 — Source Data Fig. 1 [file 44321_2024_53_MOESM2_ESM.zip › Figure 1/1C/HS Ac-TUB.tif]

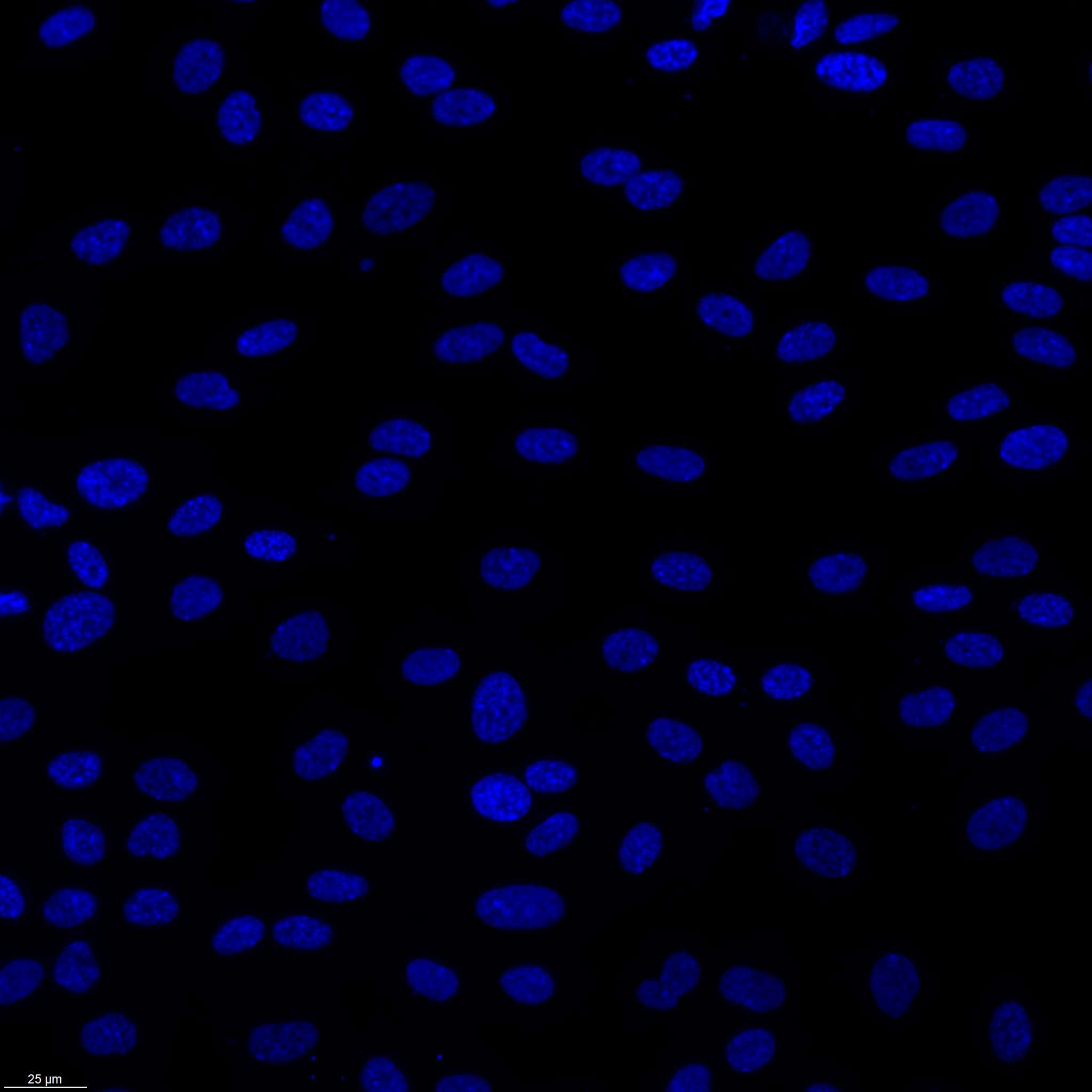

Supplement: Supplementary file 2 — Source Data Fig. 1 [file 44321_2024_53_MOESM2_ESM.zip › Figure 1/1C/HS dapi.tif]

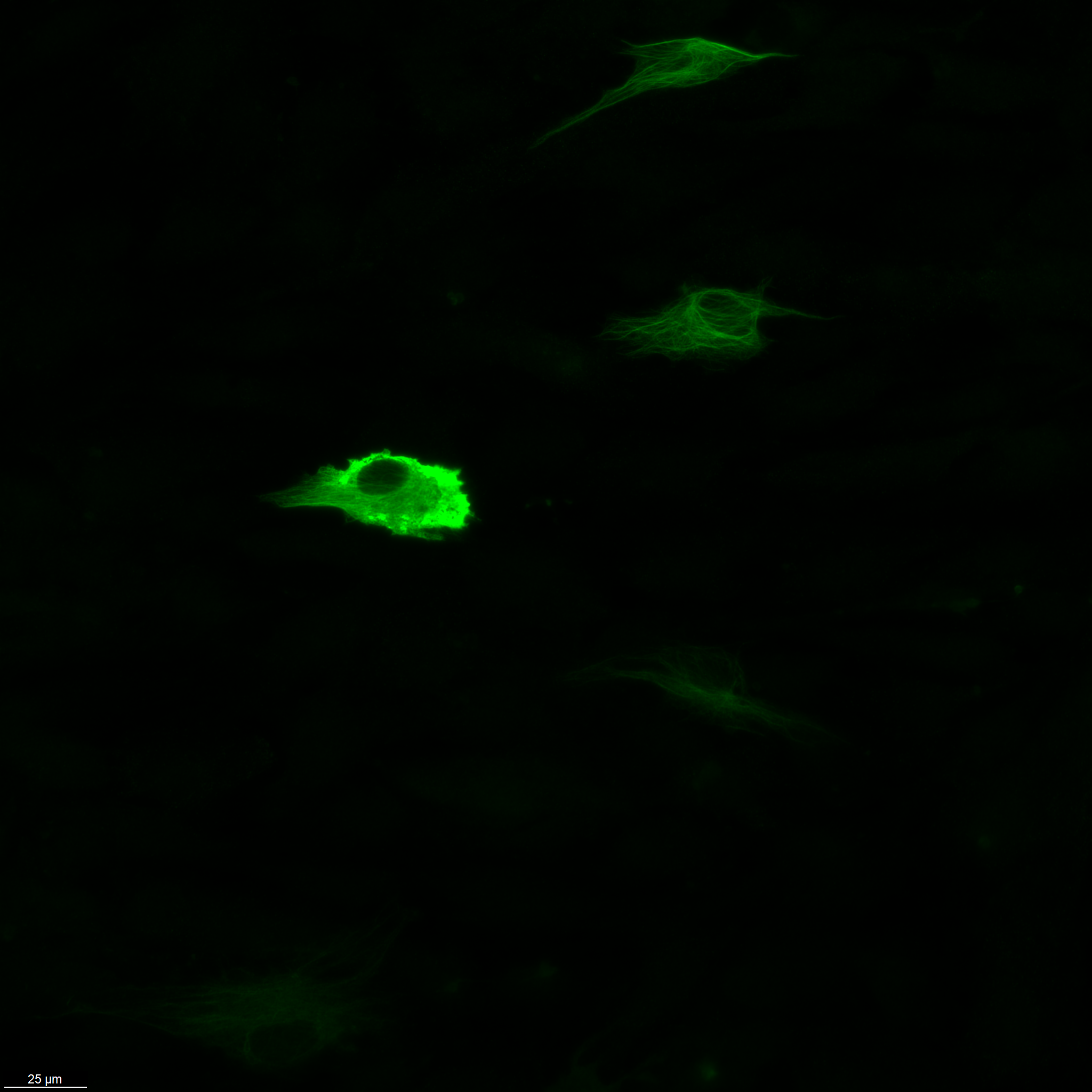

Supplement: Supplementary file 2 — Source Data Fig. 1 [file 44321_2024_53_MOESM2_ESM.zip › Figure 1/1C/HS FAM161A.tif]

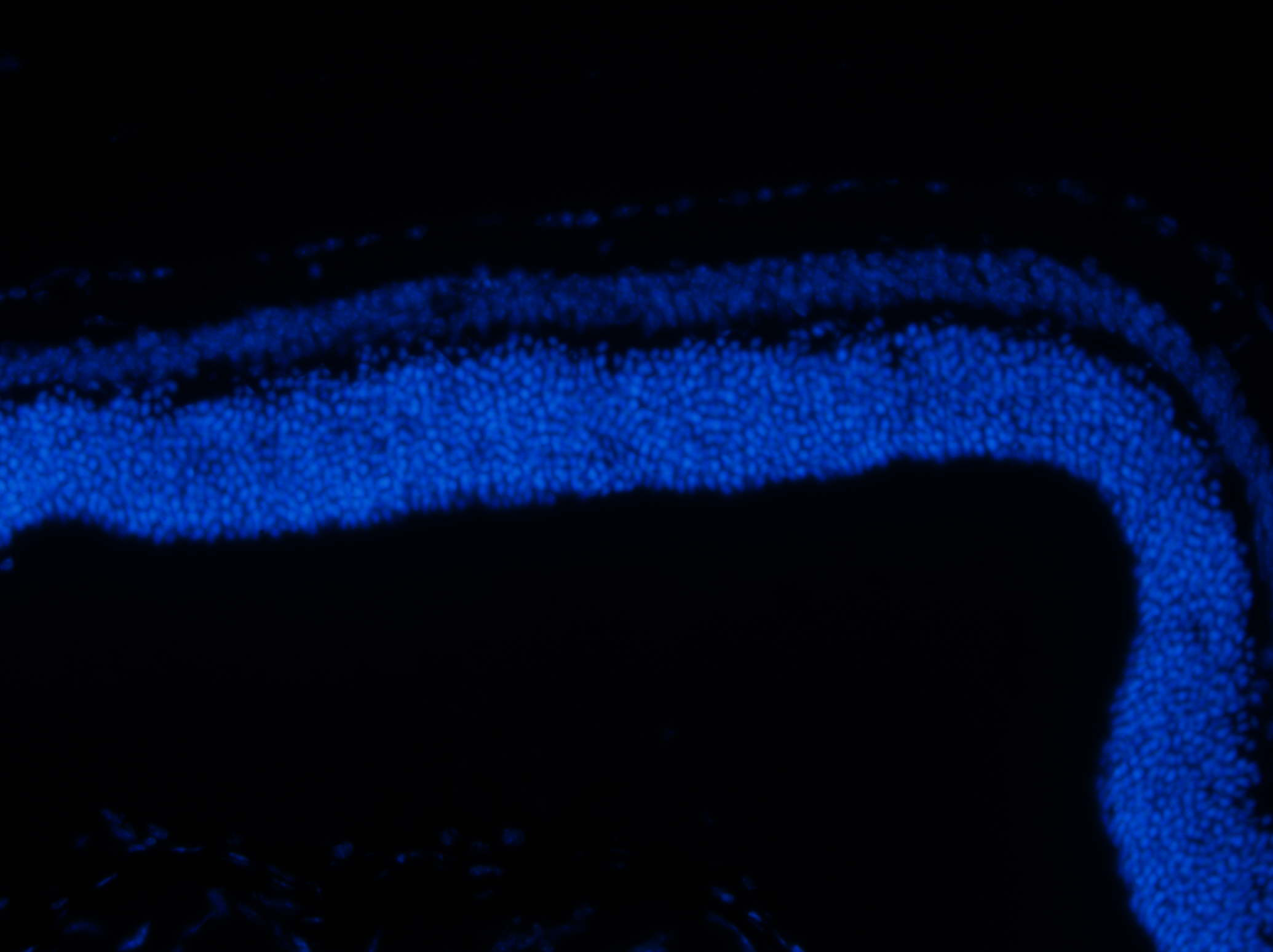

Supplement: Supplementary file 2 — Source Data Fig. 1 [file 44321_2024_53_MOESM2_ESM.zip › Figure 1/1E/DAPI.tif]

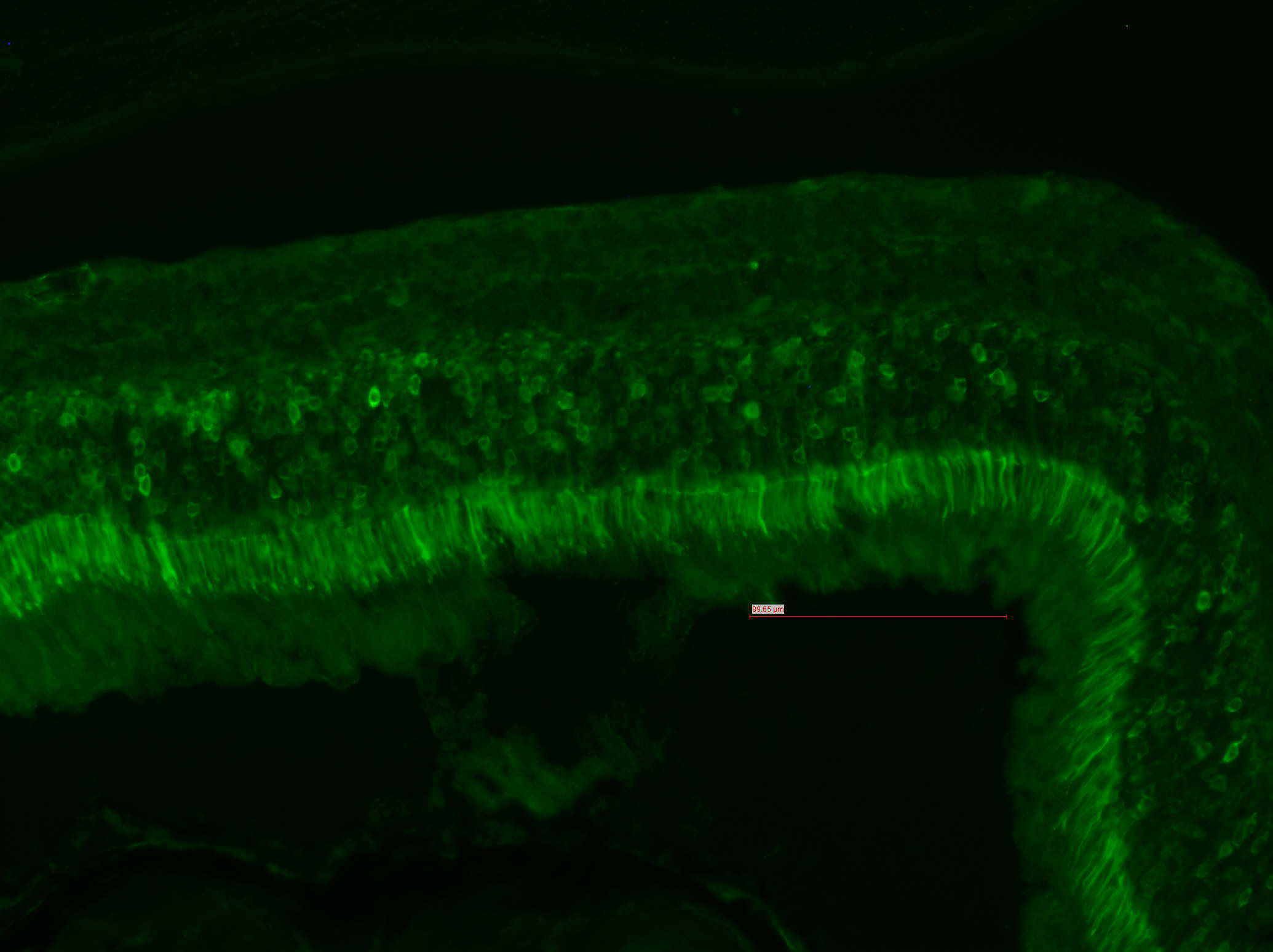

Supplement: Supplementary file 2 — Source Data Fig. 1 [file 44321_2024_53_MOESM2_ESM.zip › Figure 1/1E/GFP.tif]

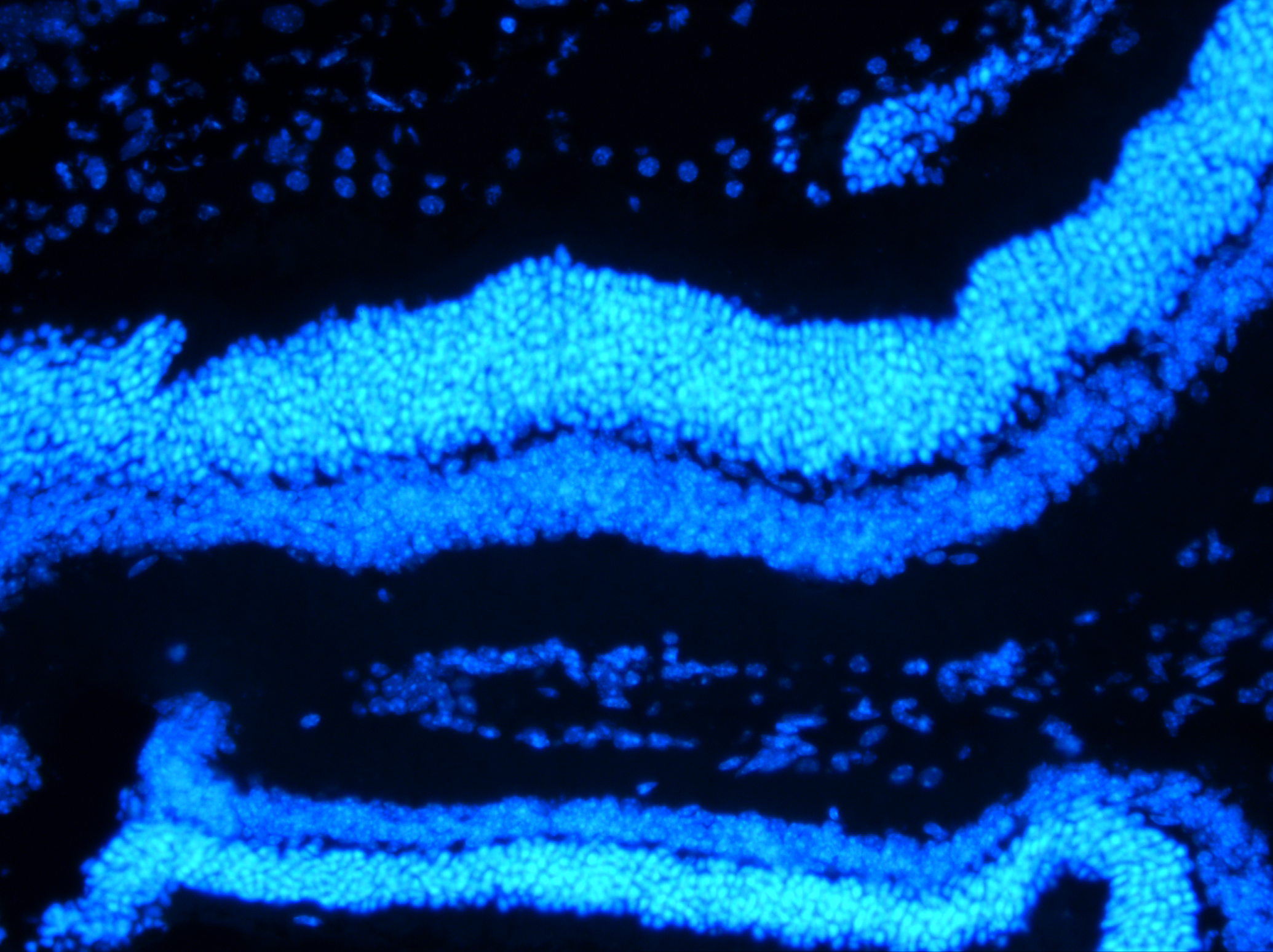

Supplement: Supplementary file 2 — Source Data Fig. 1 [file 44321_2024_53_MOESM2_ESM.zip › Figure 1/1F/DAPI.tif]

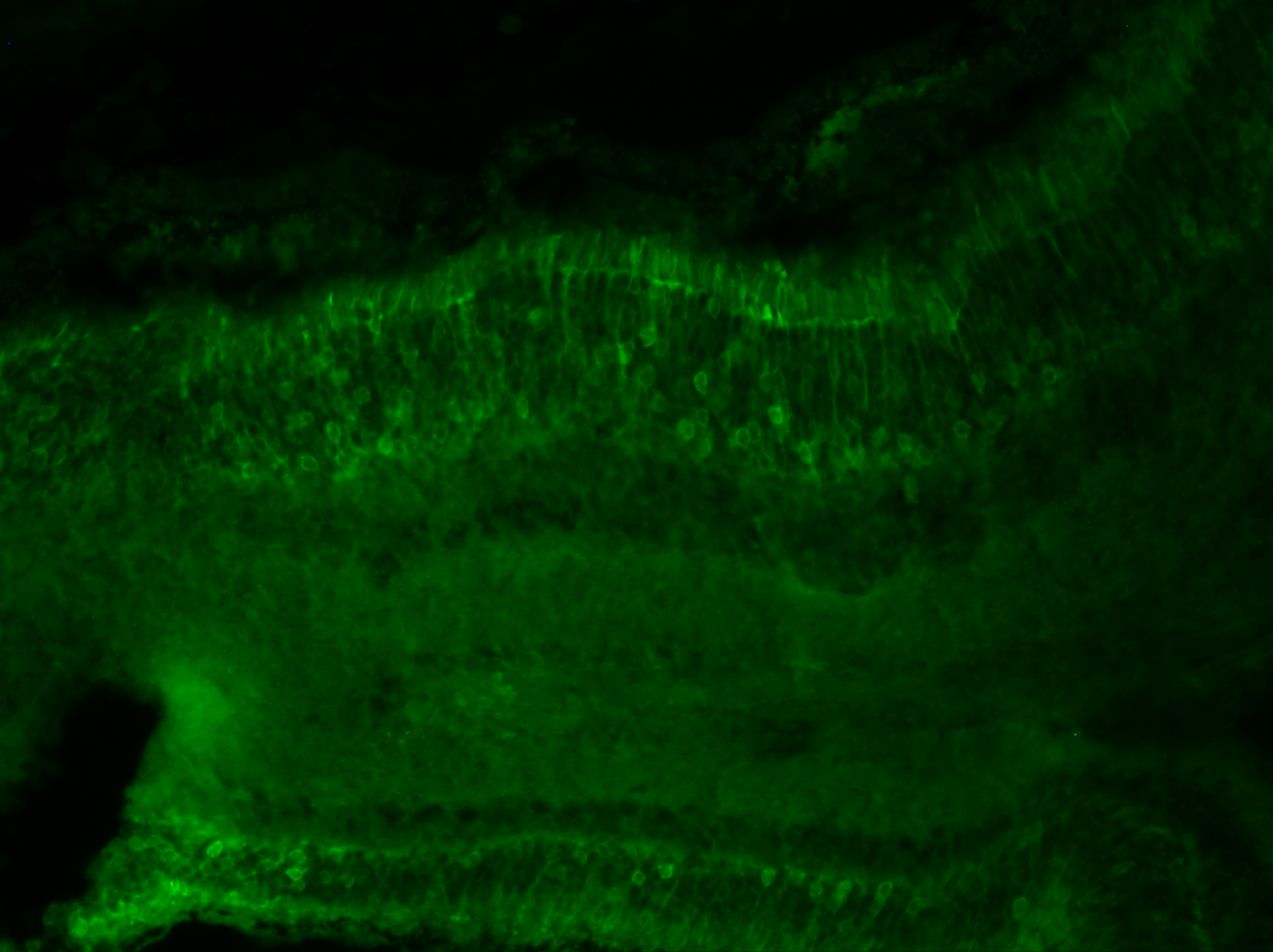

Supplement: Supplementary file 2 — Source Data Fig. 1 [file 44321_2024_53_MOESM2_ESM.zip › Figure 1/1F/GFP.tif]

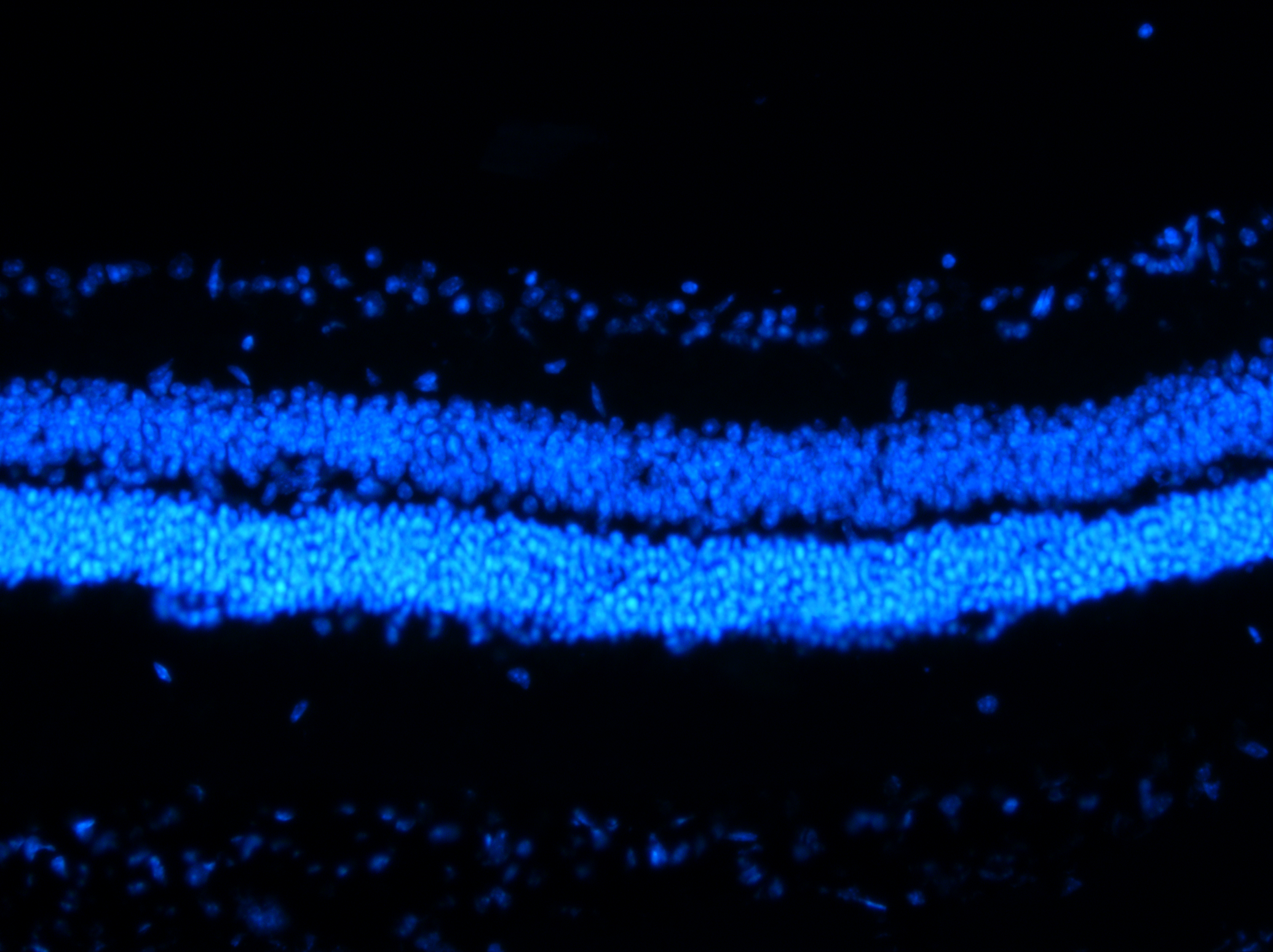

Supplement: Supplementary file 2 — Source Data Fig. 1 [file 44321_2024_53_MOESM2_ESM.zip › Figure 1/1G/DAPI.tif]

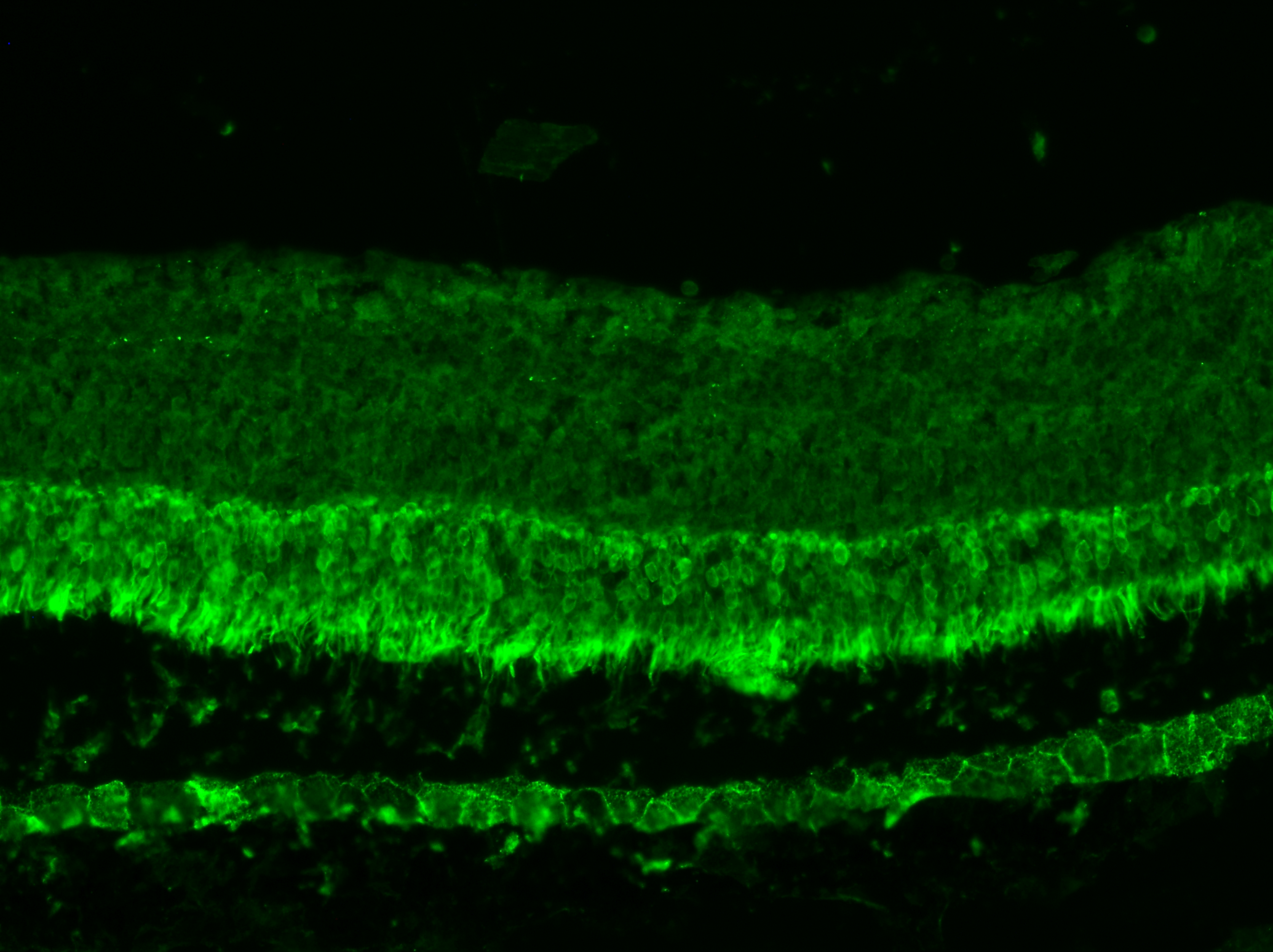

Supplement: Supplementary file 2 — Source Data Fig. 1 [file 44321_2024_53_MOESM2_ESM.zip › Figure 1/1G/GFP.tif]

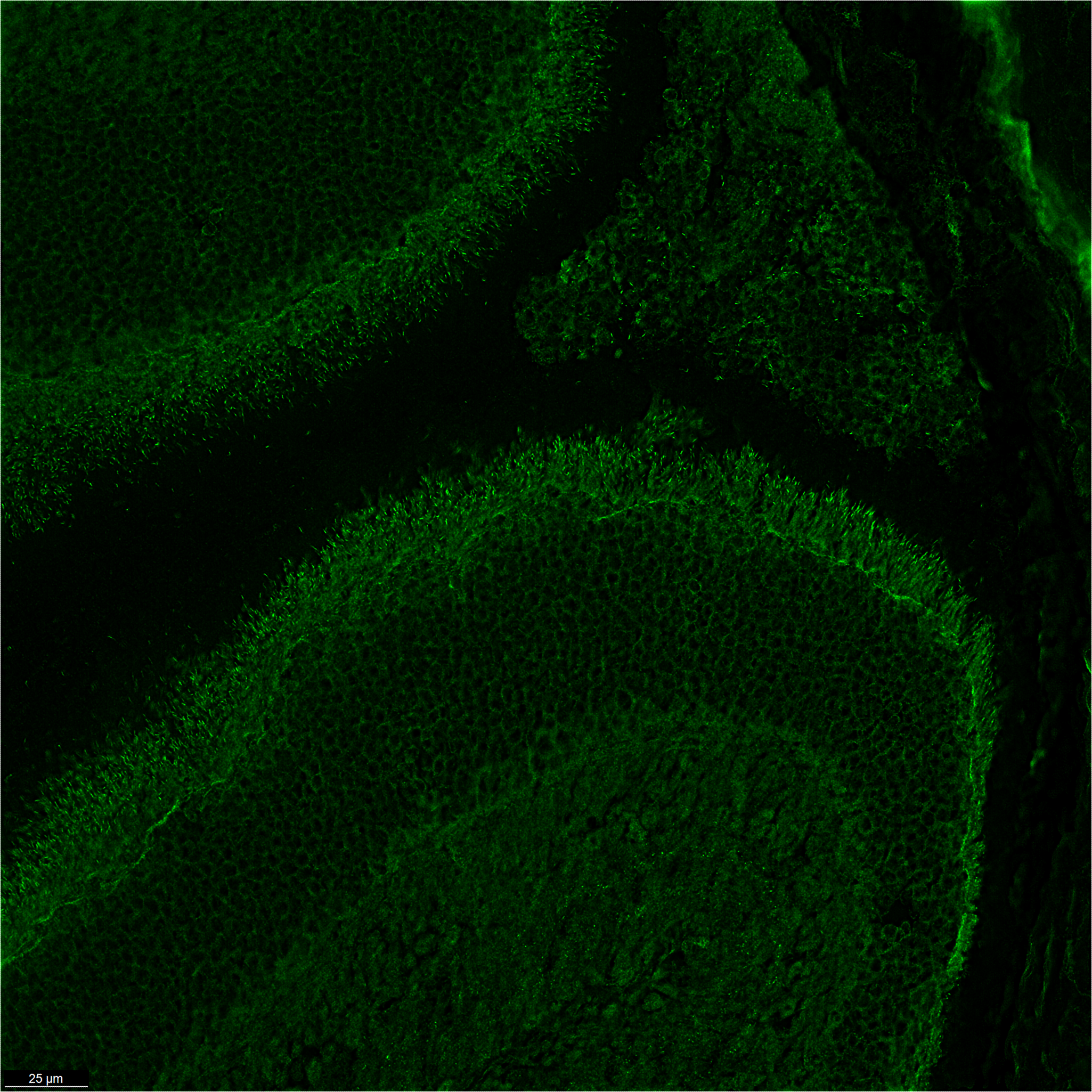

Supplement: Supplementary file 3 — Source Data Fig. 2 [file 44321_2024_53_MOESM3_ESM.zip › Figure 2/2E/FAM161A.tif]

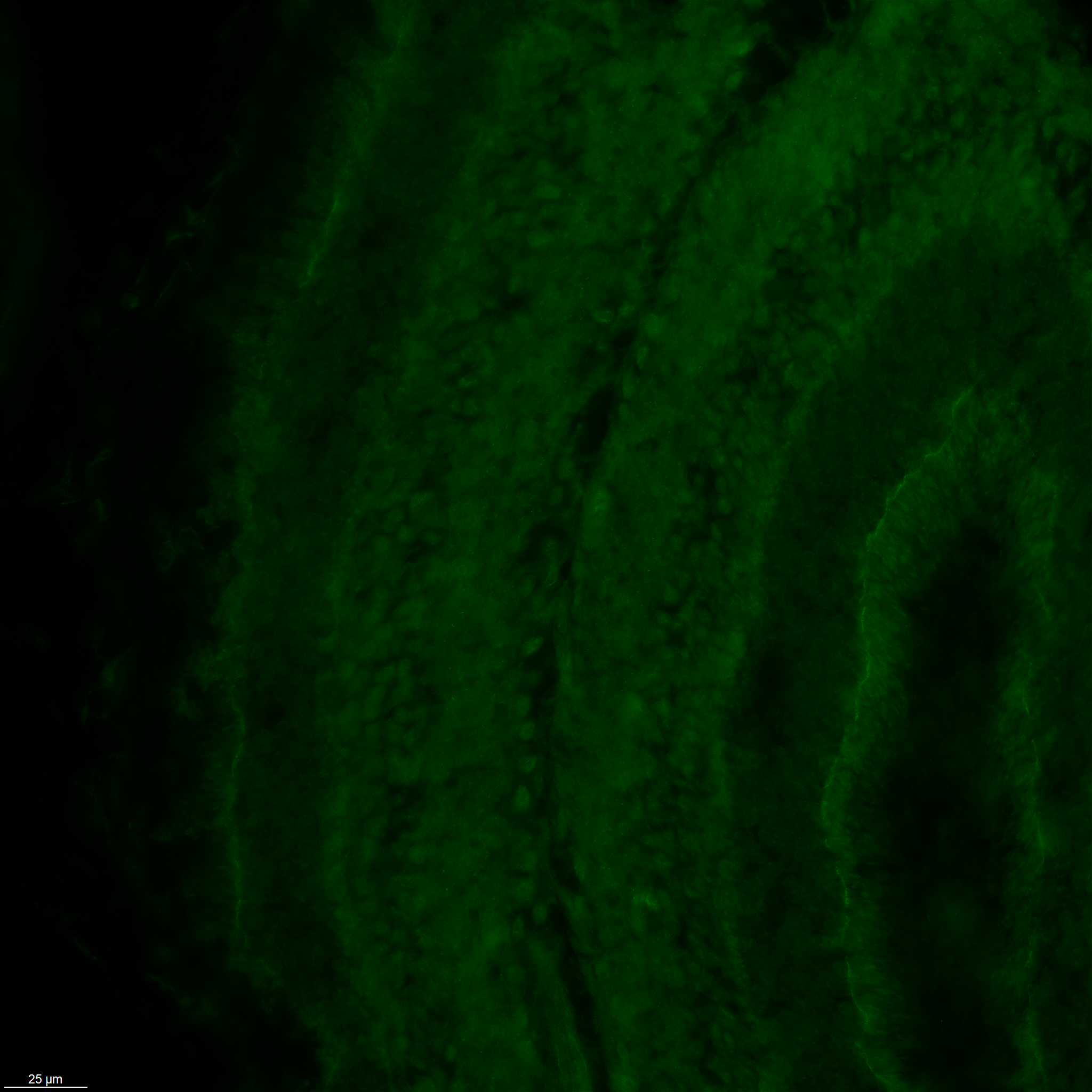

Supplement: Supplementary file 3 — Source Data Fig. 2 [file 44321_2024_53_MOESM3_ESM.zip › Figure 2/2F/FAM161A.tif]

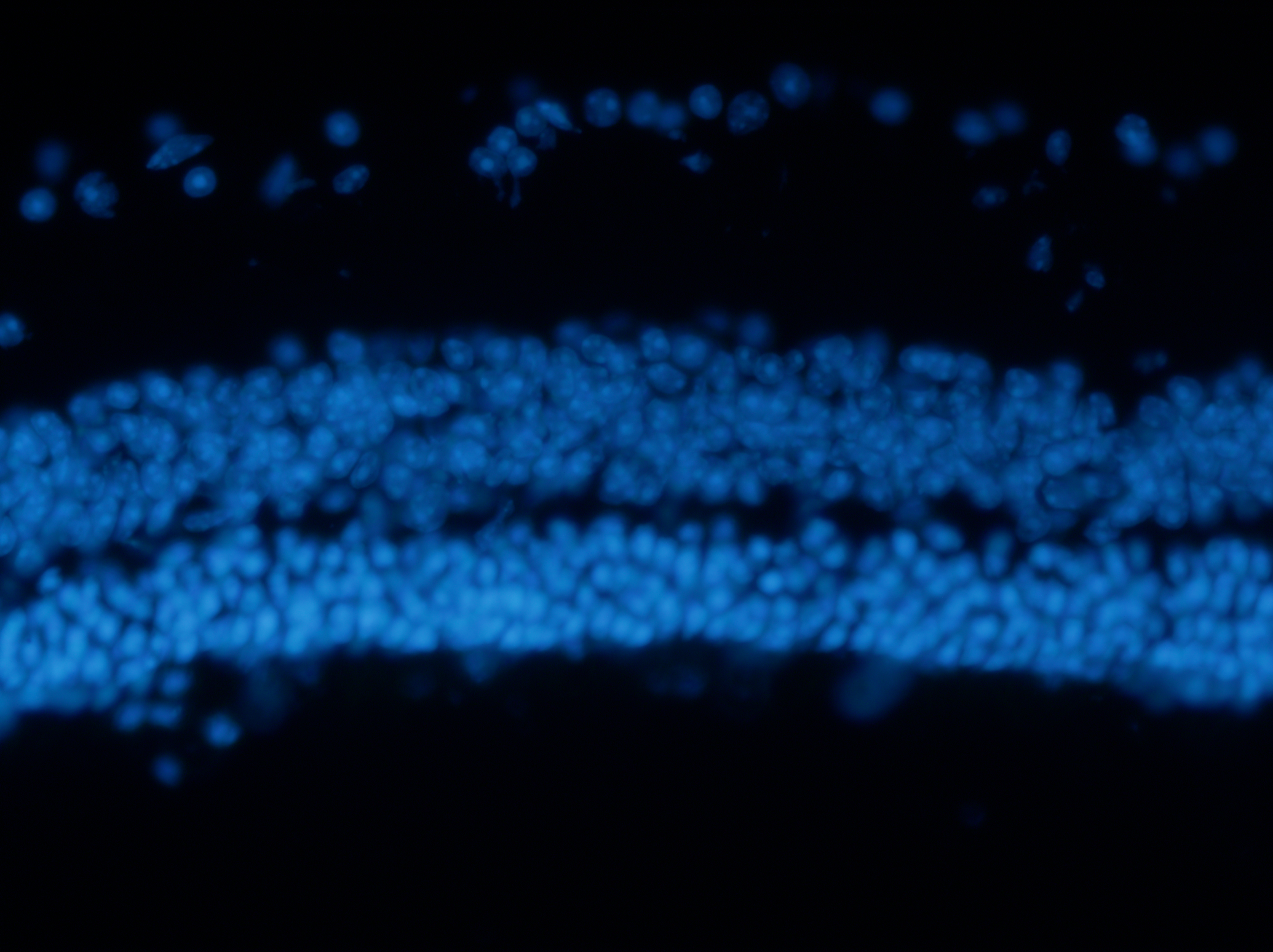

Supplement: Supplementary file 3 — Source Data Fig. 2 [file 44321_2024_53_MOESM3_ESM.zip › Figure 2/2G/DAPI.tif]

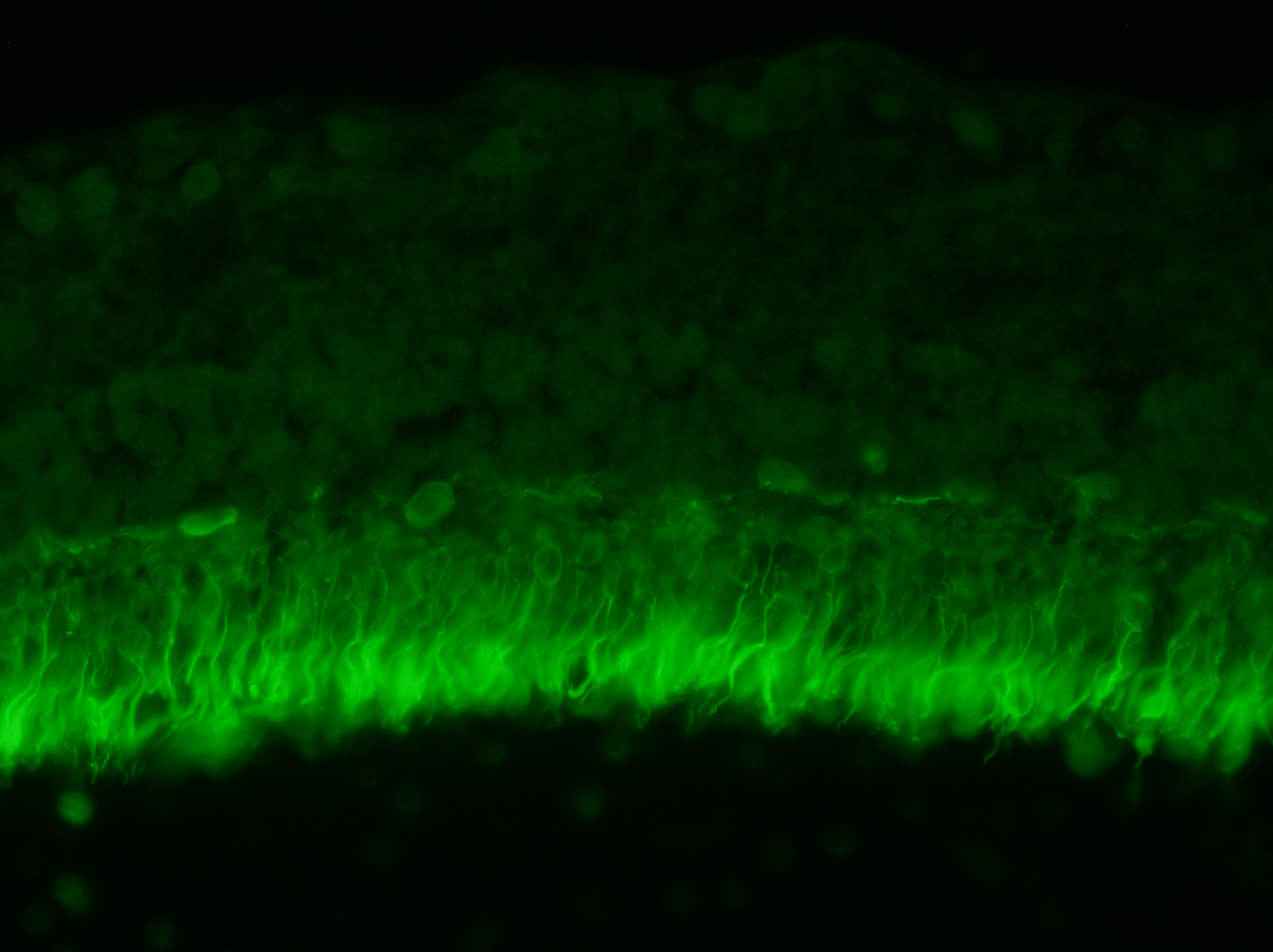

Supplement: Supplementary file 3 — Source Data Fig. 2 [file 44321_2024_53_MOESM3_ESM.zip › Figure 2/2G/FAM161A.tif]

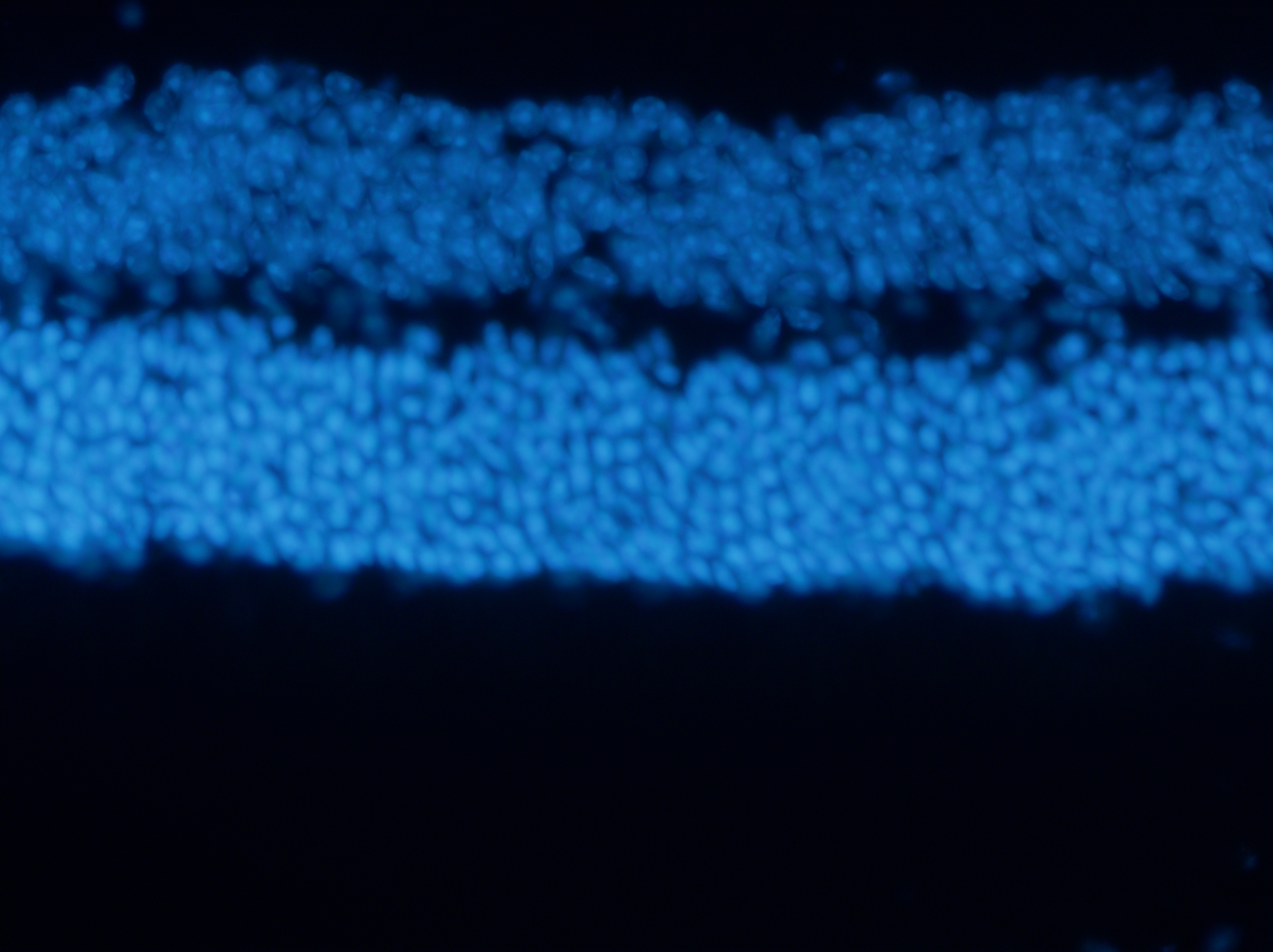

Supplement: Supplementary file 3 — Source Data Fig. 2 [file 44321_2024_53_MOESM3_ESM.zip › Figure 2/2H/DAPI.tif]

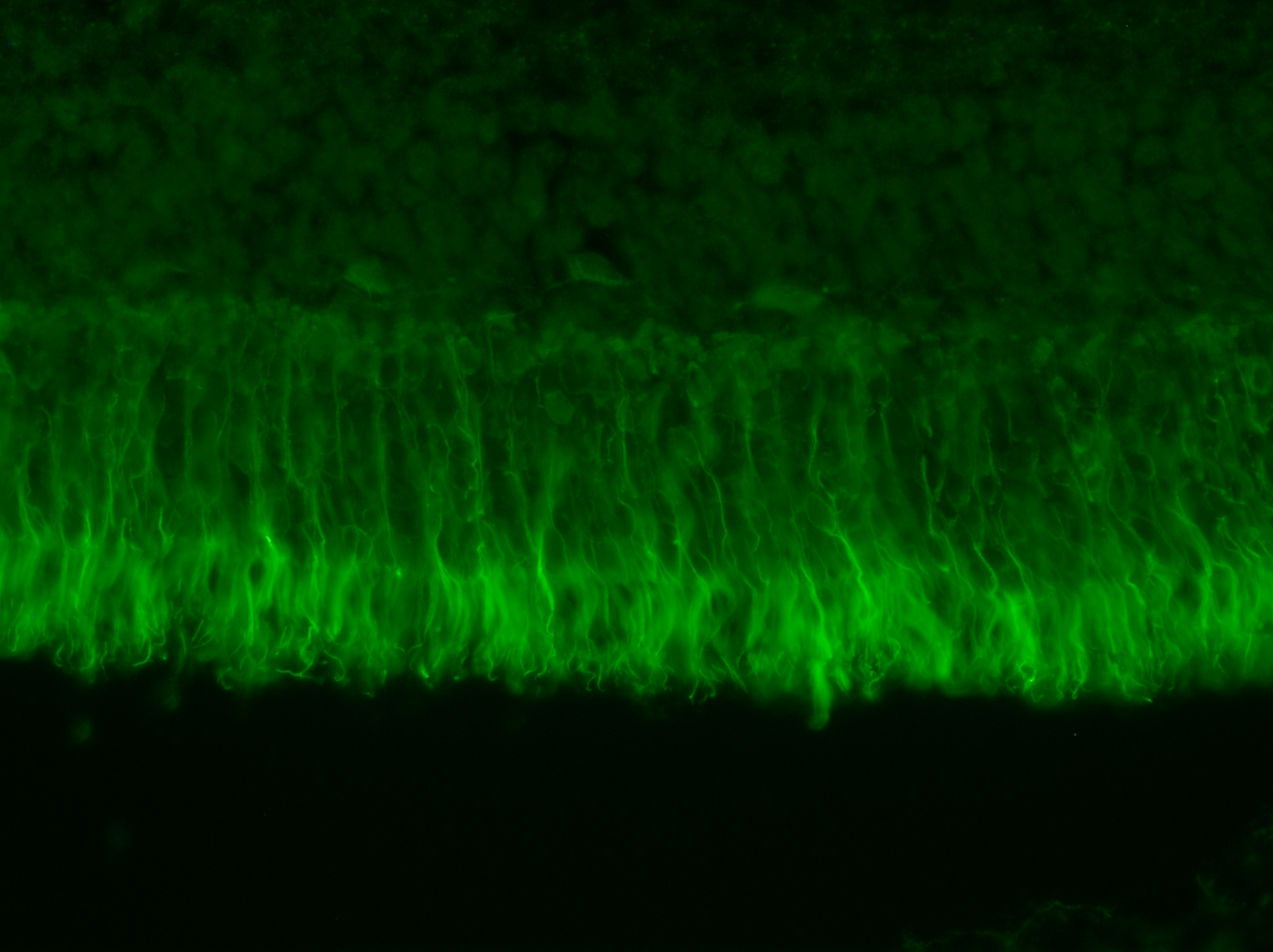

Supplement: Supplementary file 3 — Source Data Fig. 2 [file 44321_2024_53_MOESM3_ESM.zip › Figure 2/2H/FAM161A.tif]

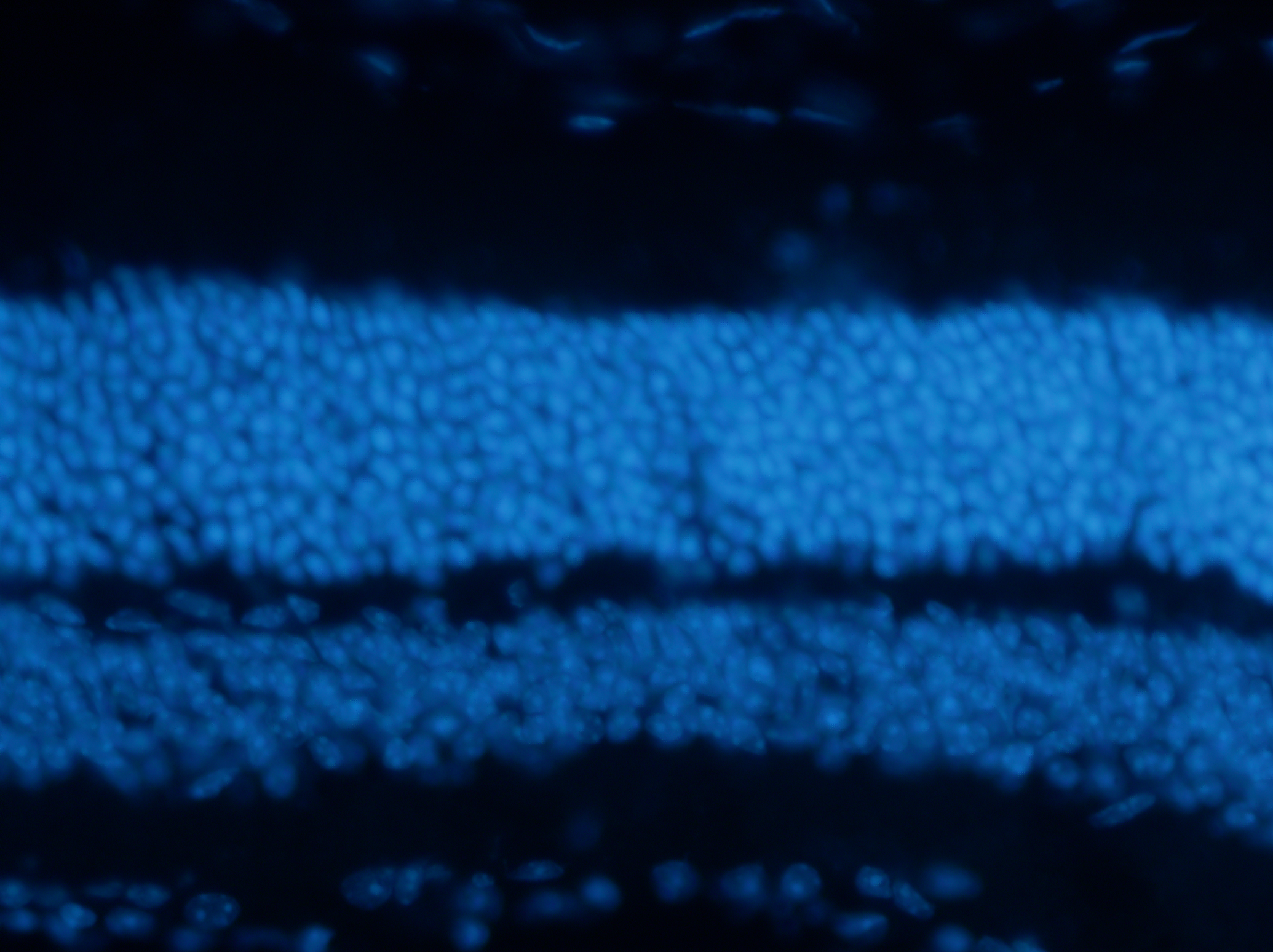

Supplement: Supplementary file 3 — Source Data Fig. 2 [file 44321_2024_53_MOESM3_ESM.zip › Figure 2/2I/DAPI.tif]

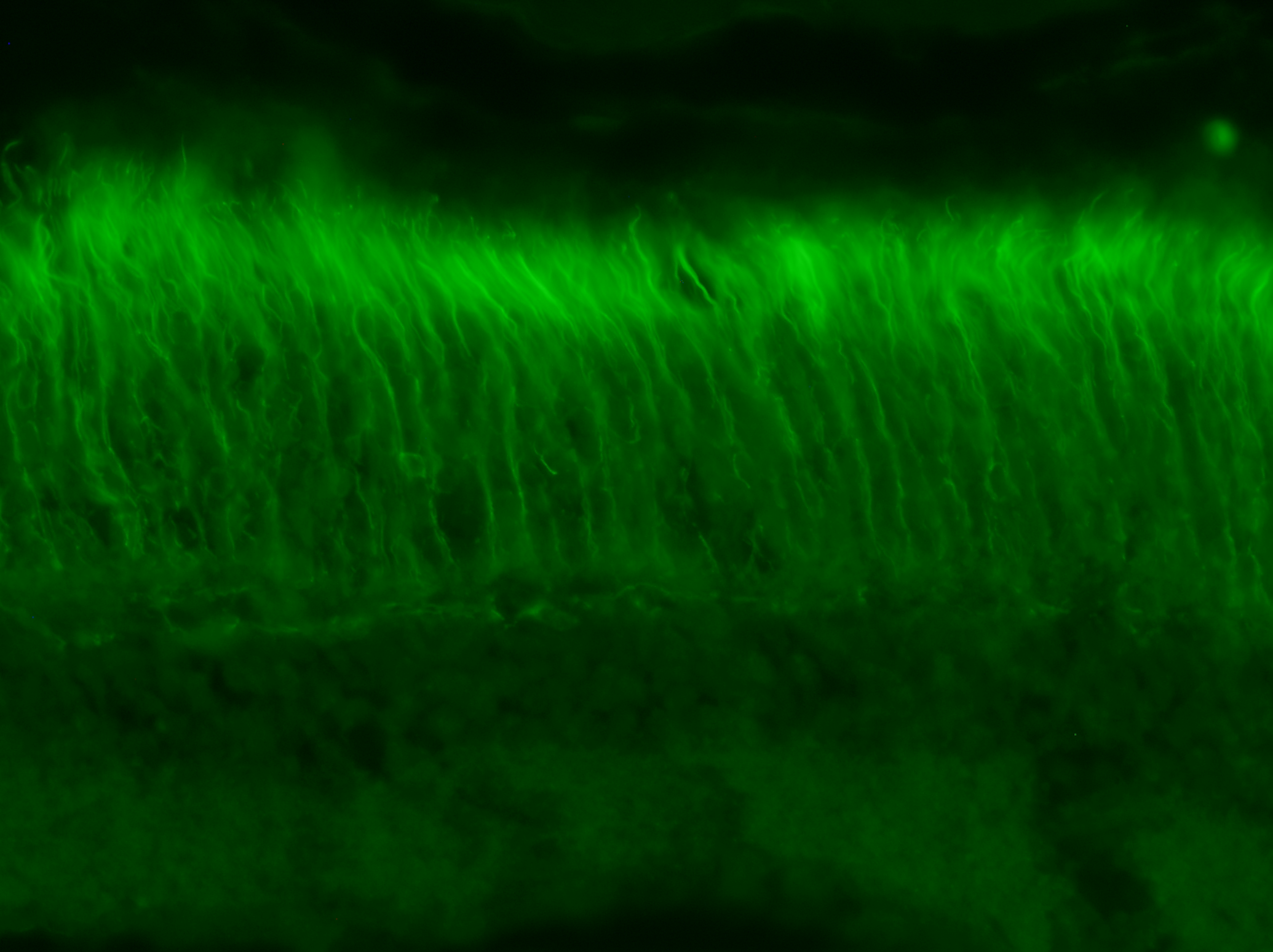

Supplement: Supplementary file 3 — Source Data Fig. 2 [file 44321_2024_53_MOESM3_ESM.zip › Figure 2/2I/FAM161A.tif]

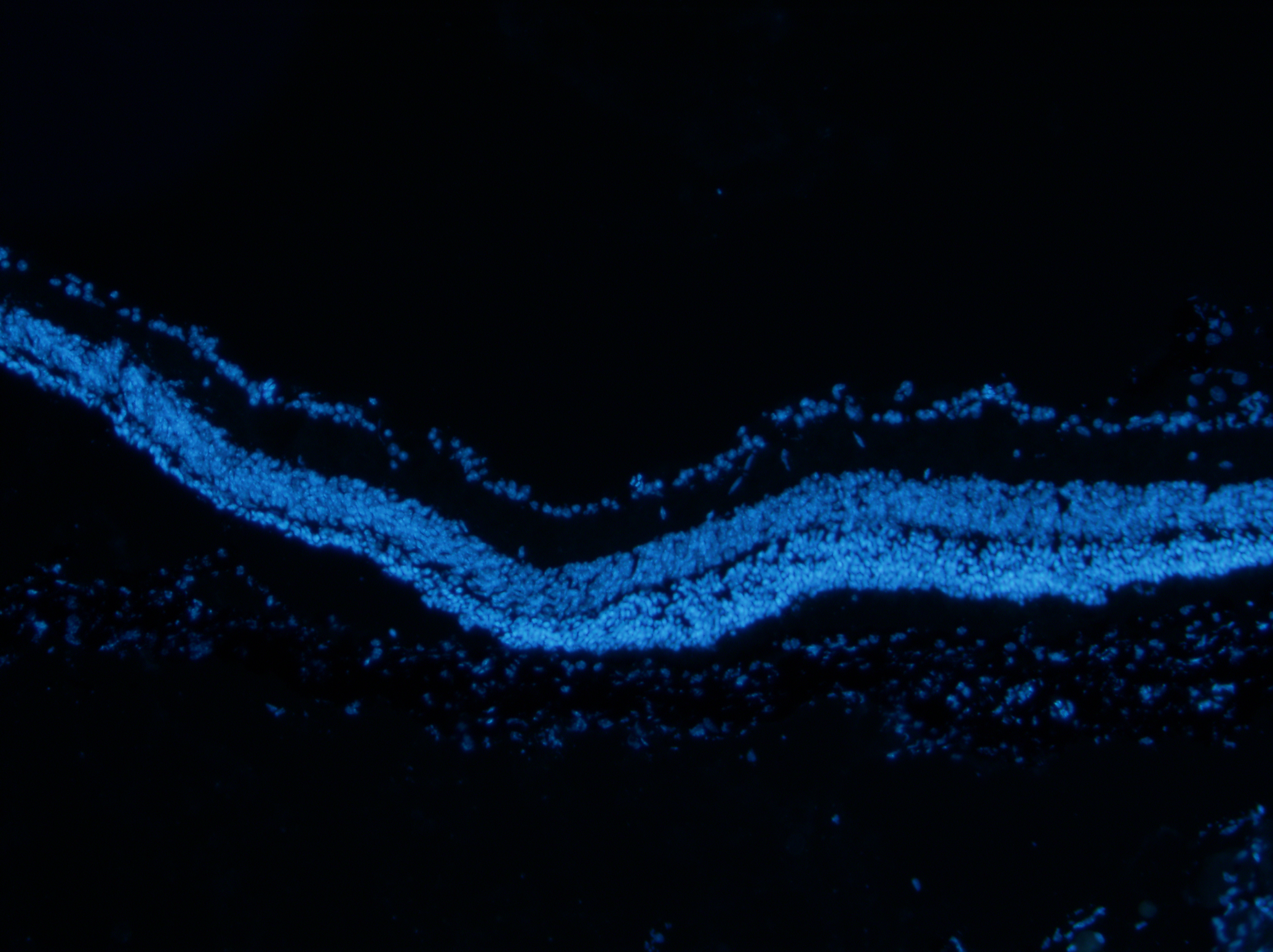

Supplement: Supplementary file 3 — Source Data Fig. 2 [file 44321_2024_53_MOESM3_ESM.zip › Figure 2/2J_K/J DAPI.tif]

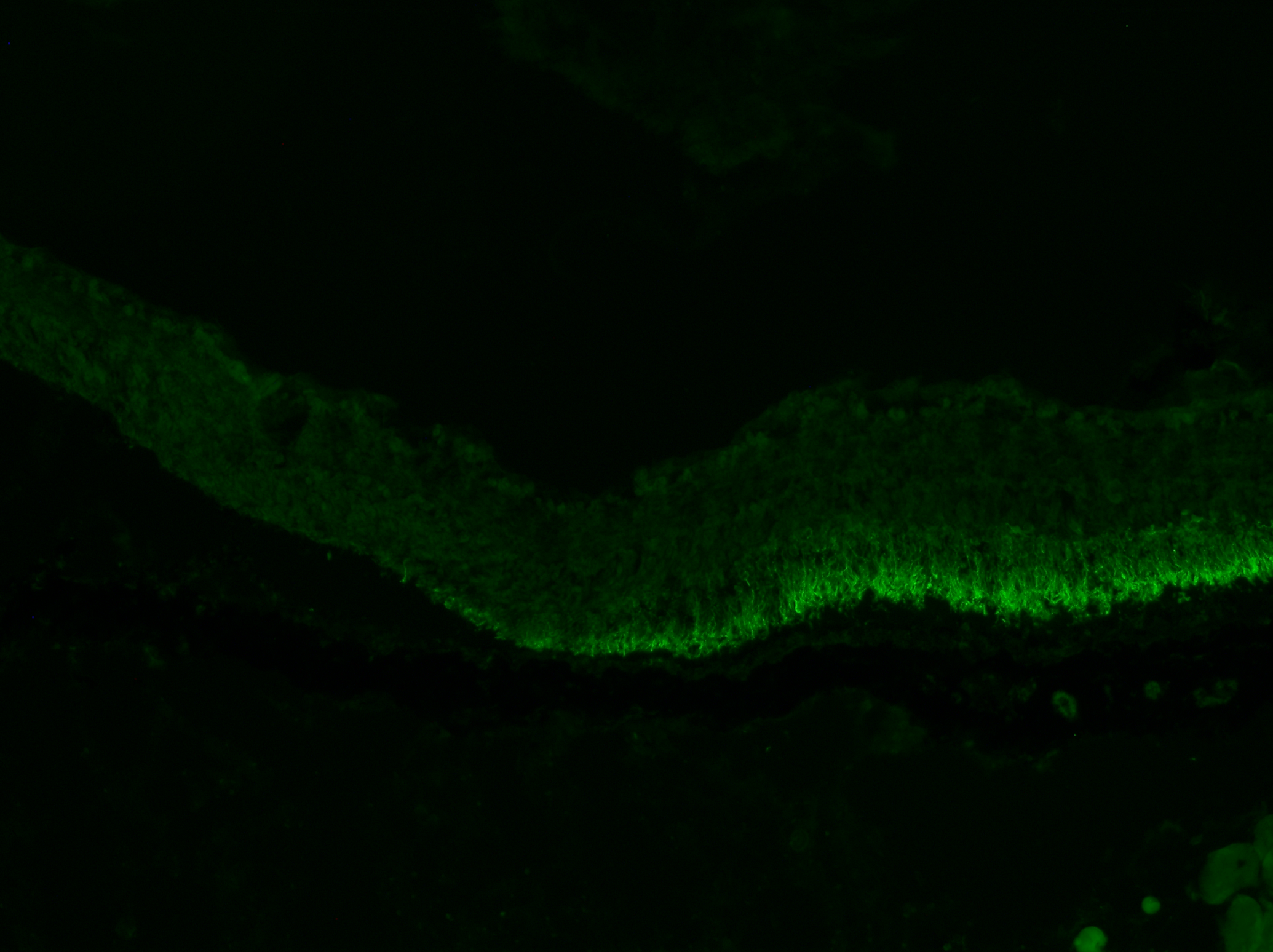

Supplement: Supplementary file 3 — Source Data Fig. 2 [file 44321_2024_53_MOESM3_ESM.zip › Figure 2/2J_K/K FAM161A.tif]

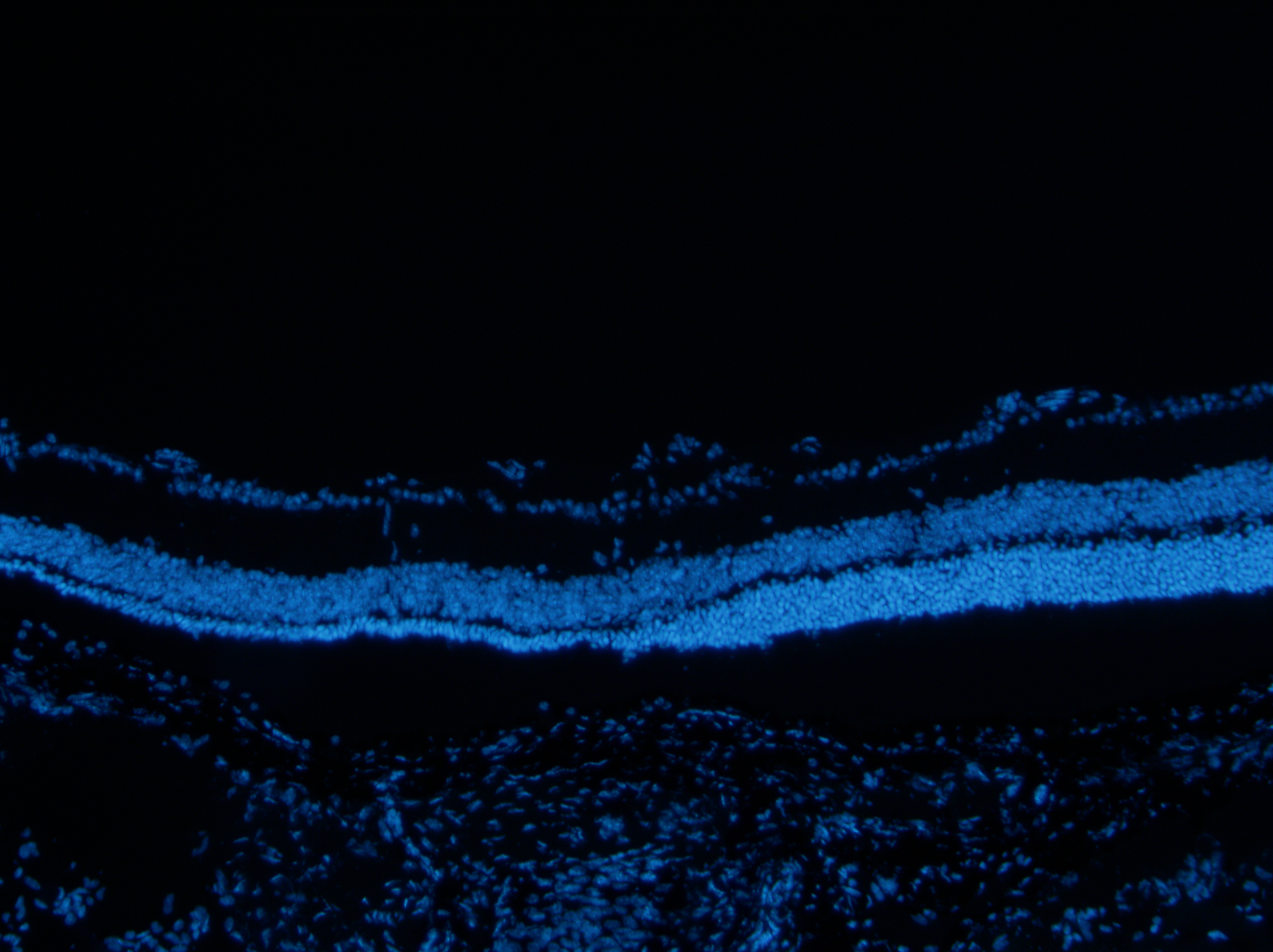

Supplement: Supplementary file 3 — Source Data Fig. 2 [file 44321_2024_53_MOESM3_ESM.zip › Figure 2/2L_M/L DAPI.tif]

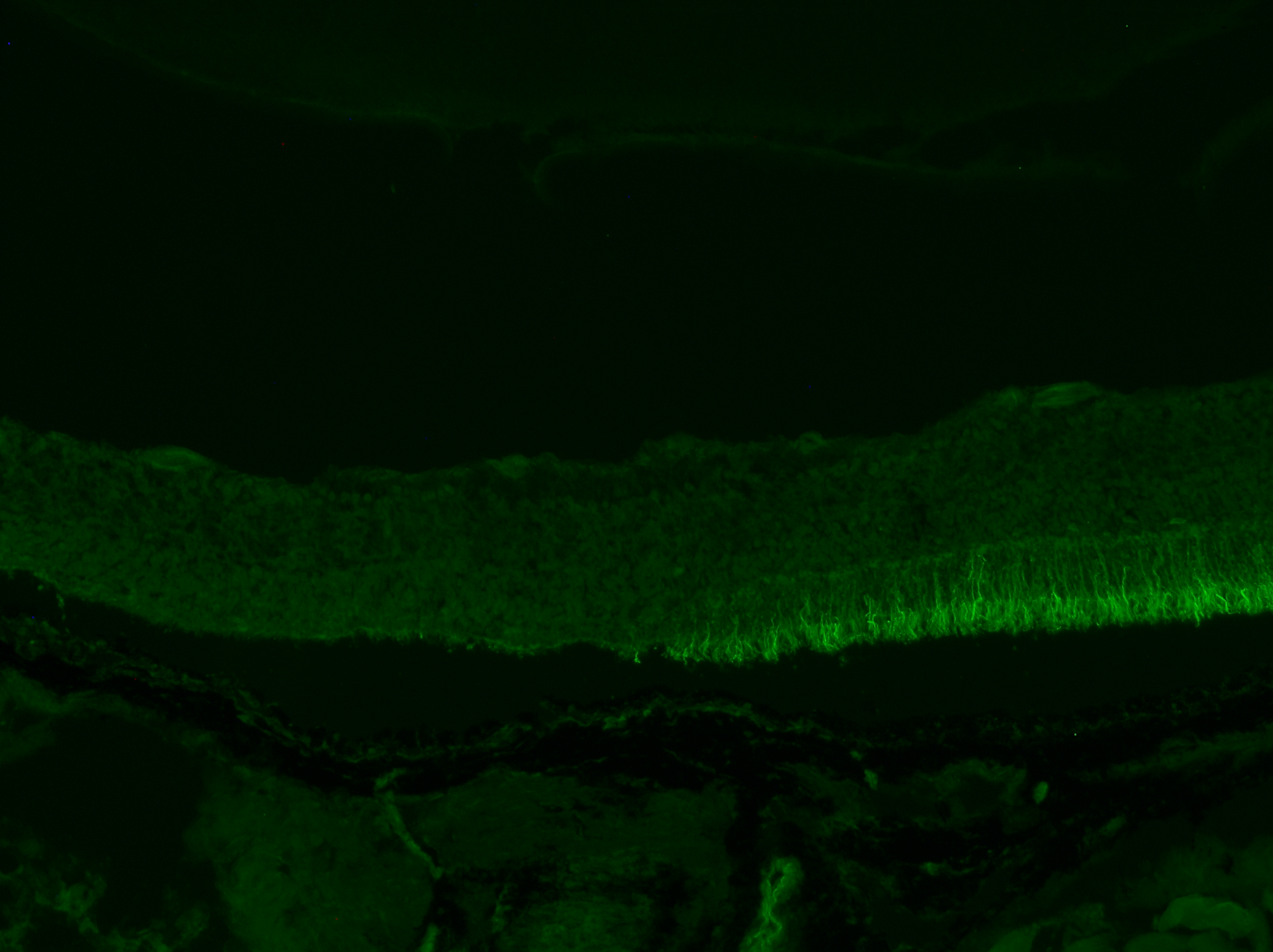

Supplement: Supplementary file 3 — Source Data Fig. 2 [file 44321_2024_53_MOESM3_ESM.zip › Figure 2/2L_M/M FAM161A.tif]

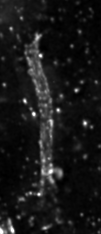

Supplement: Supplementary file 4 — Source Data Fig. 3 [file 44321_2024_53_MOESM4_ESM.zip › Figure 3/IRBP GRK1 HS + HL/Treated region/Cep290.tif]

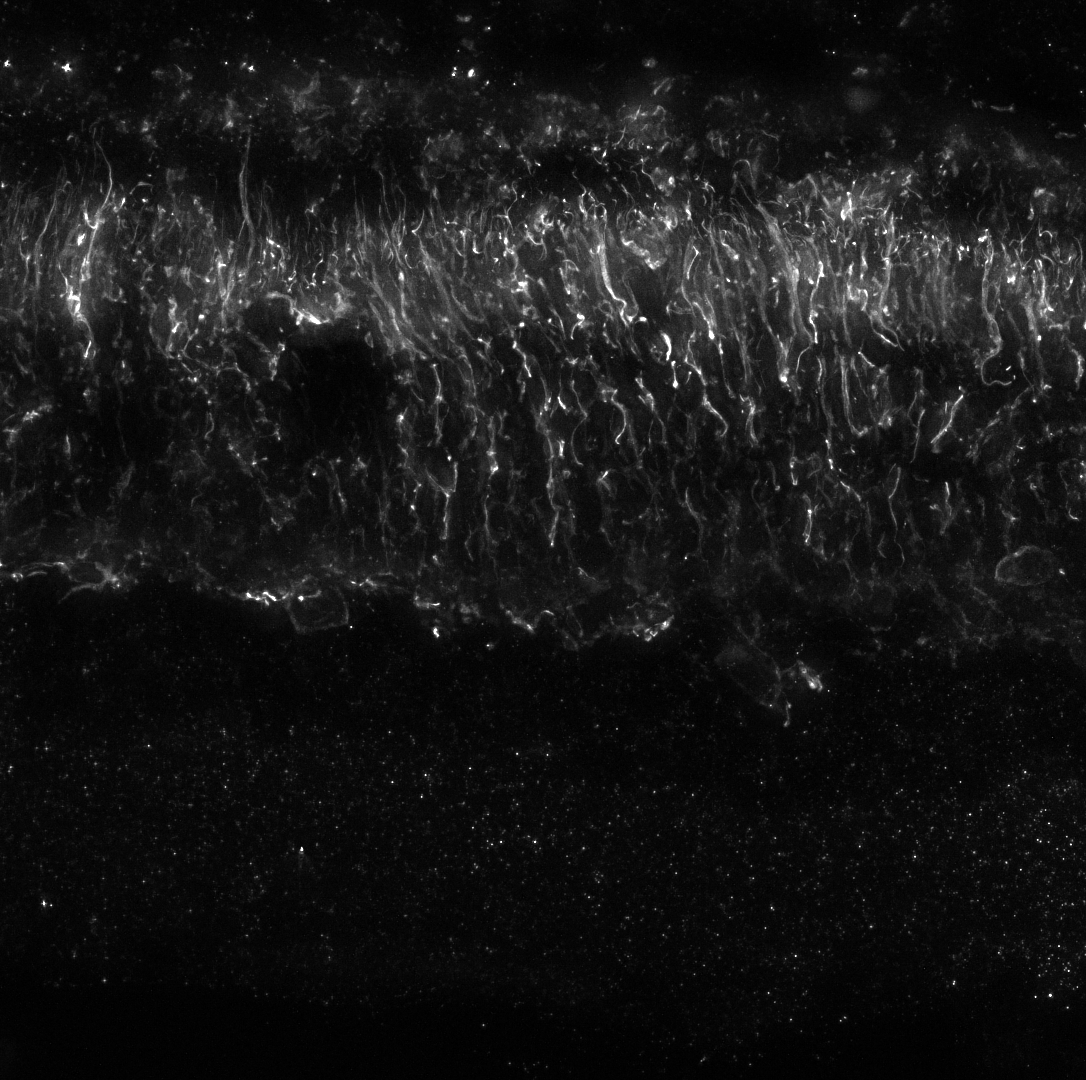

Supplement: Supplementary file 4 — Source Data Fig. 3 [file 44321_2024_53_MOESM4_ESM.zip › Figure 3/IRBP GRK1 HS + HL/Treated region/Fam161a Low mag.tif]

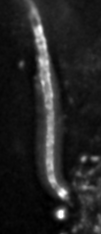

Supplement: Supplementary file 4 — Source Data Fig. 3 [file 44321_2024_53_MOESM4_ESM.zip › Figure 3/IRBP GRK1 HS + HL/Treated region/Fam161a.tif]

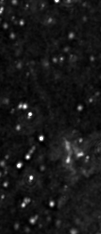

Supplement: Supplementary file 4 — Source Data Fig. 3 [file 44321_2024_53_MOESM4_ESM.zip › Figure 3/IRBP GRK1 HS + HL/Treated region/IFT81.tif]

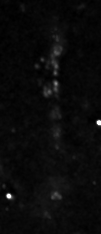

Supplement: Supplementary file 4 — Source Data Fig. 3 [file 44321_2024_53_MOESM4_ESM.zip › Figure 3/IRBP GRK1 HS + HL/Treated region/LCA5.tif]

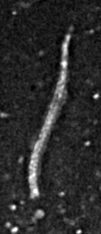

Supplement: Supplementary file 4 — Source Data Fig. 3 [file 44321_2024_53_MOESM4_ESM.zip › Figure 3/IRBP GRK1 HS + HL/Treated region/POC5.tif]

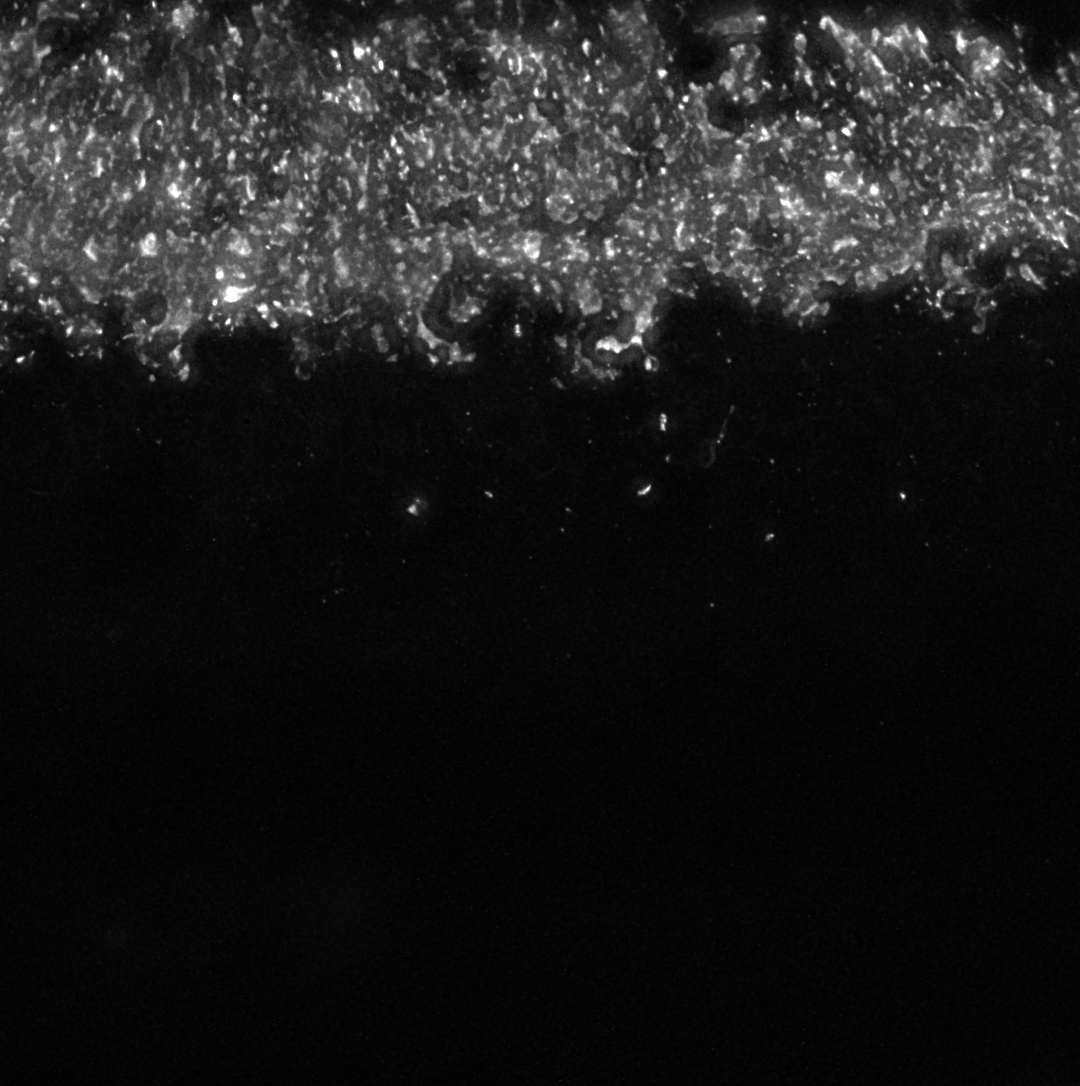

Supplement: Supplementary file 4 — Source Data Fig. 3 [file 44321_2024_53_MOESM4_ESM.zip › Figure 3/IRBP GRK1 HS + HL/Treated region/Rhodopsin.tif]

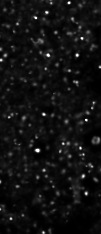

Supplement: Supplementary file 4 — Source Data Fig. 3 [file 44321_2024_53_MOESM4_ESM.zip › Figure 3/IRBP GRK1 HS + HL/untreated region/Cep290.tif]

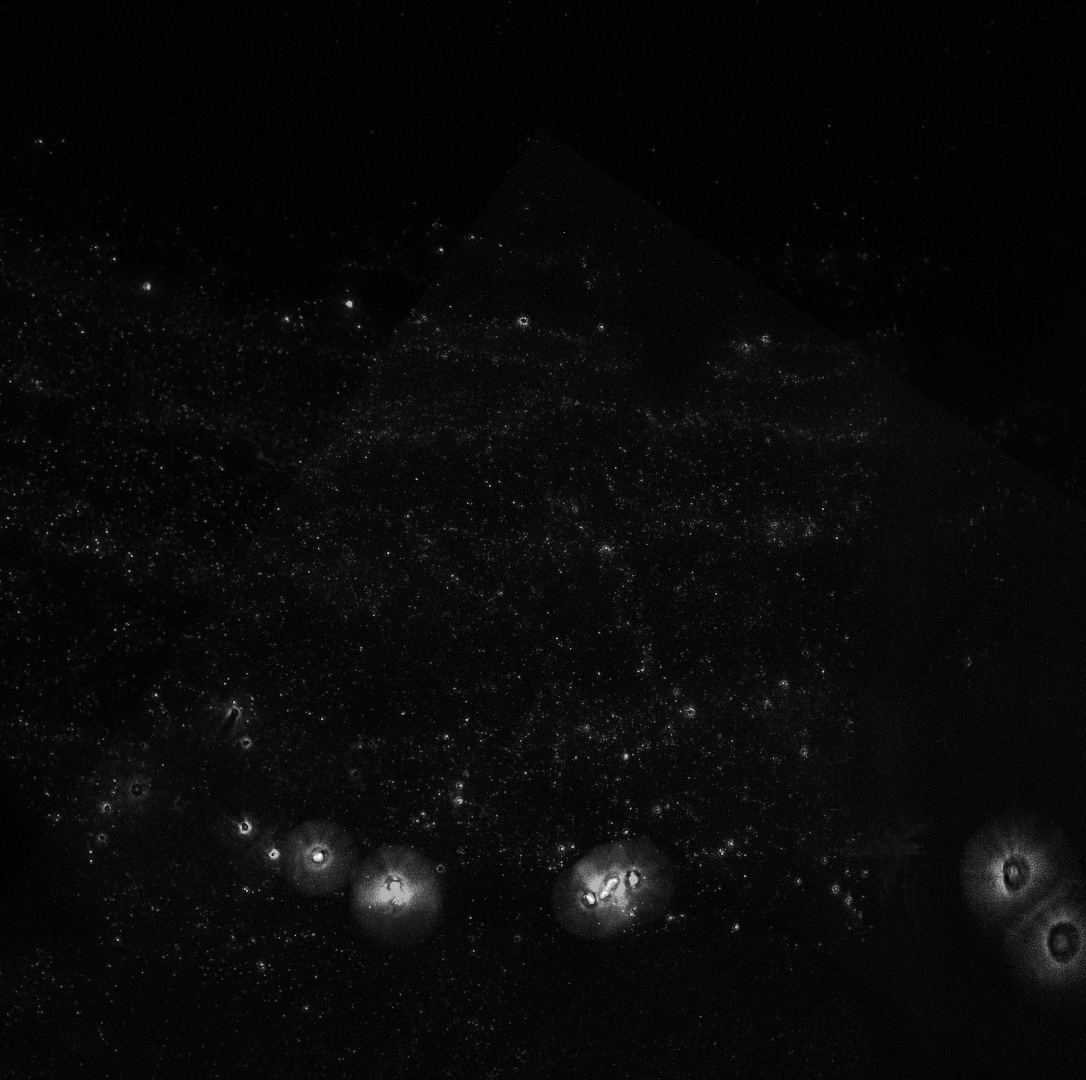

Supplement: Supplementary file 4 — Source Data Fig. 3 [file 44321_2024_53_MOESM4_ESM.zip › Figure 3/IRBP GRK1 HS + HL/untreated region/Fam161a low mag.tif]

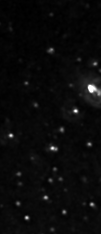

Supplement: Supplementary file 4 — Source Data Fig. 3 [file 44321_2024_53_MOESM4_ESM.zip › Figure 3/IRBP GRK1 HS + HL/untreated region/Fam161a.tif]

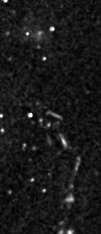

Supplement: Supplementary file 4 — Source Data Fig. 3 [file 44321_2024_53_MOESM4_ESM.zip › Figure 3/IRBP GRK1 HS + HL/untreated region/IFT81.tif]

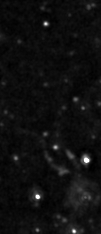

Supplement: Supplementary file 4 — Source Data Fig. 3 [file 44321_2024_53_MOESM4_ESM.zip › Figure 3/IRBP GRK1 HS + HL/untreated region/Lca5.tif]

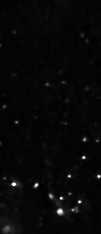

Supplement: Supplementary file 4 — Source Data Fig. 3 [file 44321_2024_53_MOESM4_ESM.zip › Figure 3/IRBP GRK1 HS + HL/untreated region/Poc5.tif]

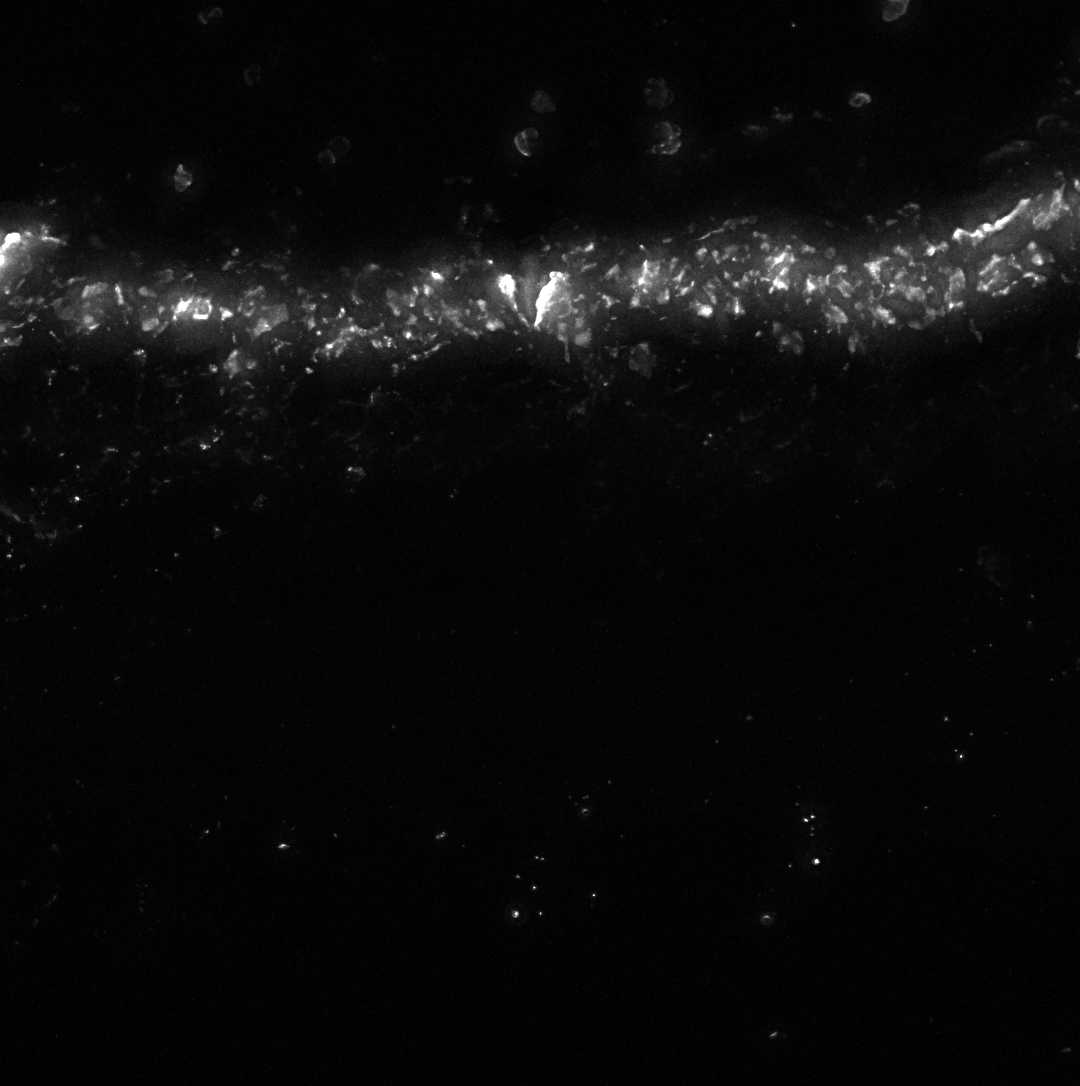

Supplement: Supplementary file 4 — Source Data Fig. 3 [file 44321_2024_53_MOESM4_ESM.zip › Figure 3/IRBP GRK1 HS + HL/untreated region/Rhodopsin.tif]

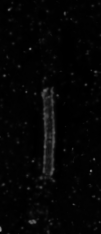

Supplement: Supplementary file 4 — Source Data Fig. 3 [file 44321_2024_53_MOESM4_ESM.zip › Figure 3/WT/Cep290.tif]

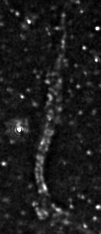

Supplement: Supplementary file 4 — Source Data Fig. 3 [file 44321_2024_53_MOESM4_ESM.zip › Figure 3/WT/Fam161a.tif]

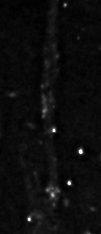

Supplement: Supplementary file 4 — Source Data Fig. 3 [file 44321_2024_53_MOESM4_ESM.zip › Figure 3/WT/IFT81.tif]

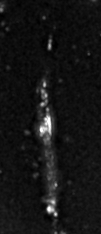

Supplement: Supplementary file 4 — Source Data Fig. 3 [file 44321_2024_53_MOESM4_ESM.zip › Figure 3/WT/Lca5.tif]

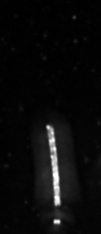

Supplement: Supplementary file 4 — Source Data Fig. 3 [file 44321_2024_53_MOESM4_ESM.zip › Figure 3/WT/Poc5.tif]

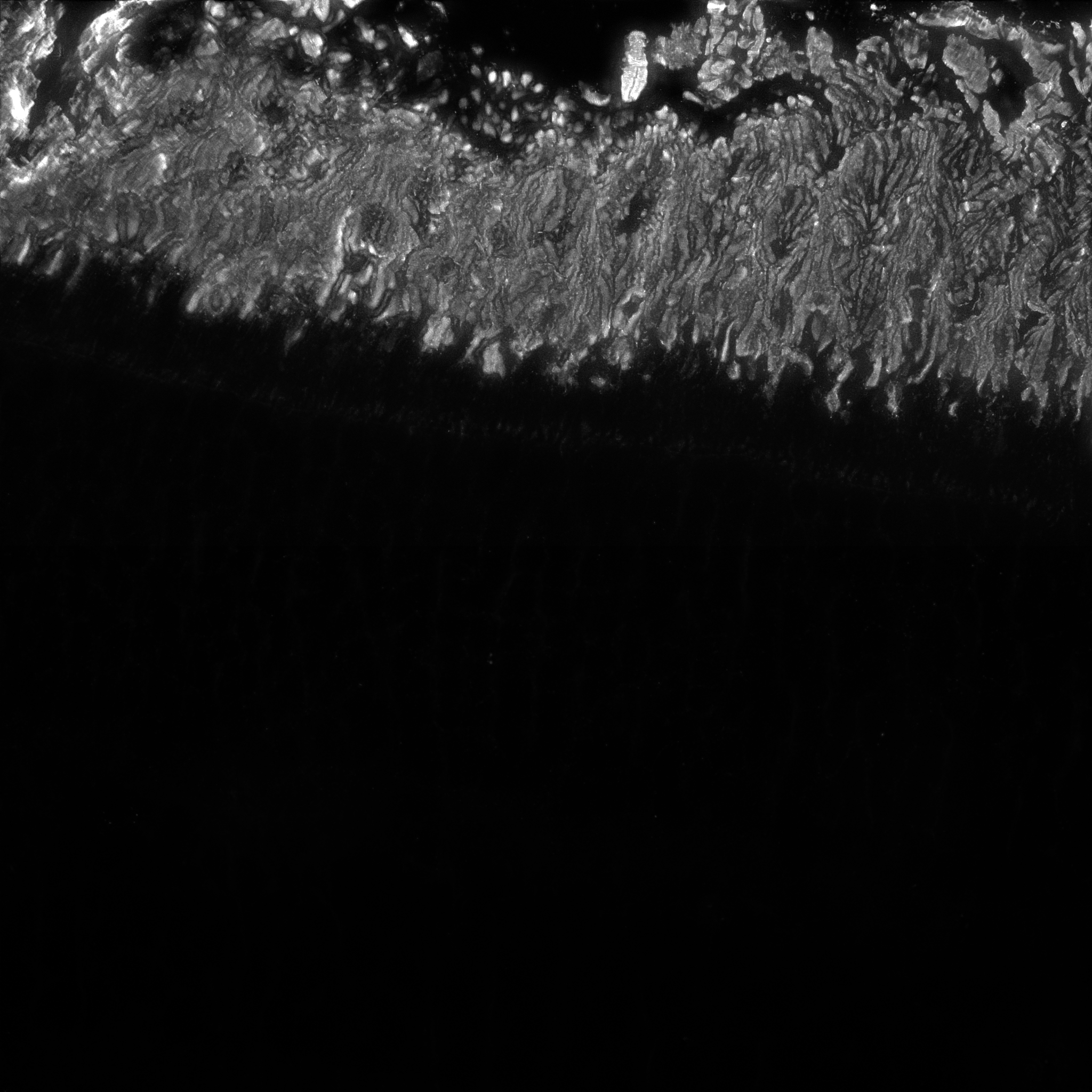

Supplement: Supplementary file 4 — Source Data Fig. 3 [file 44321_2024_53_MOESM4_ESM.zip › Figure 3/WT/Rhodopsin.tif]

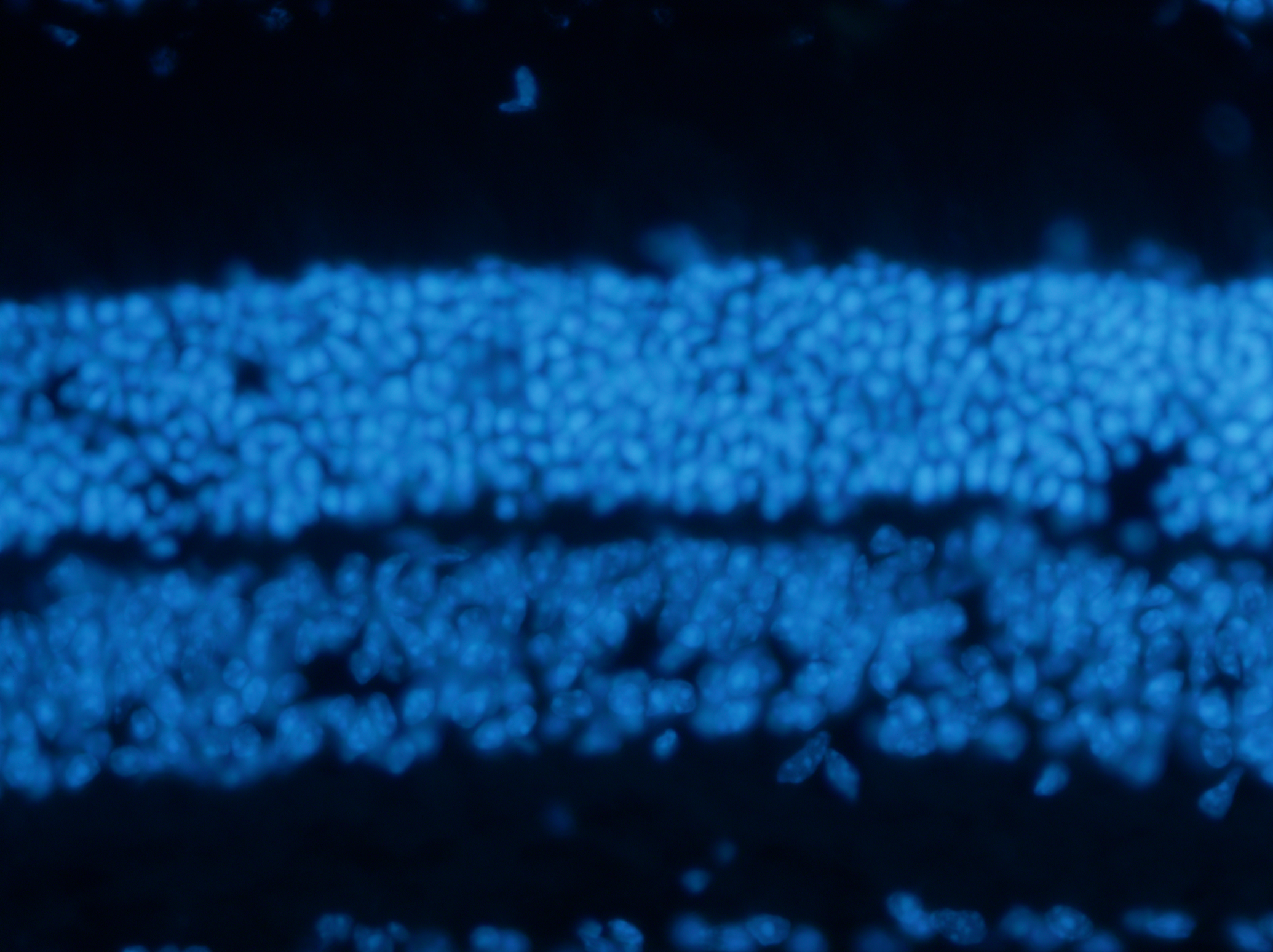

Supplement: Supplementary file 5 — Source Data Fig. 4 [file 44321_2024_53_MOESM5_ESM.zip › Figure 4/4A/DAPI.tif]

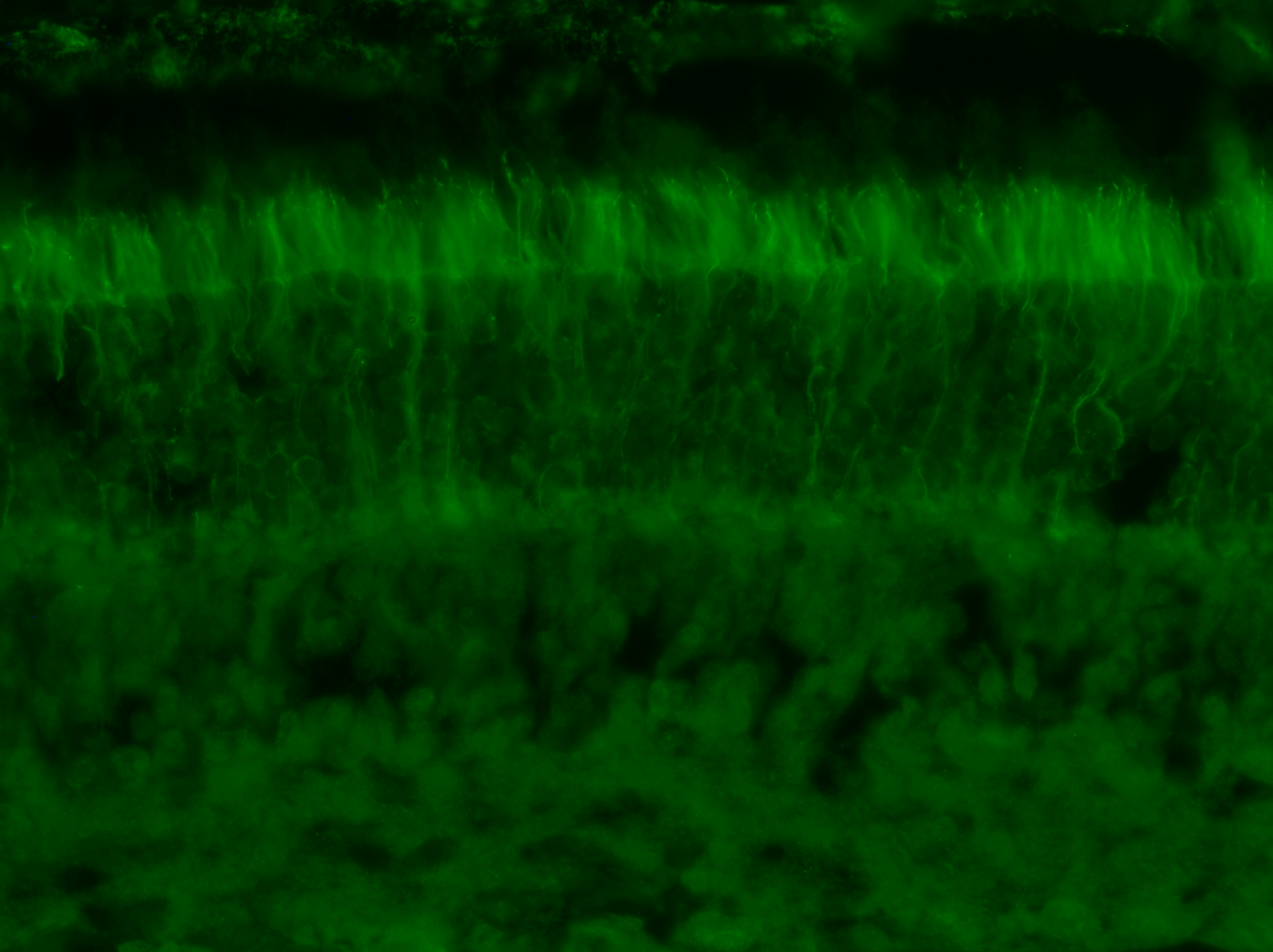

Supplement: Supplementary file 5 — Source Data Fig. 4 [file 44321_2024_53_MOESM5_ESM.zip › Figure 4/4A/FAM161A.tif]

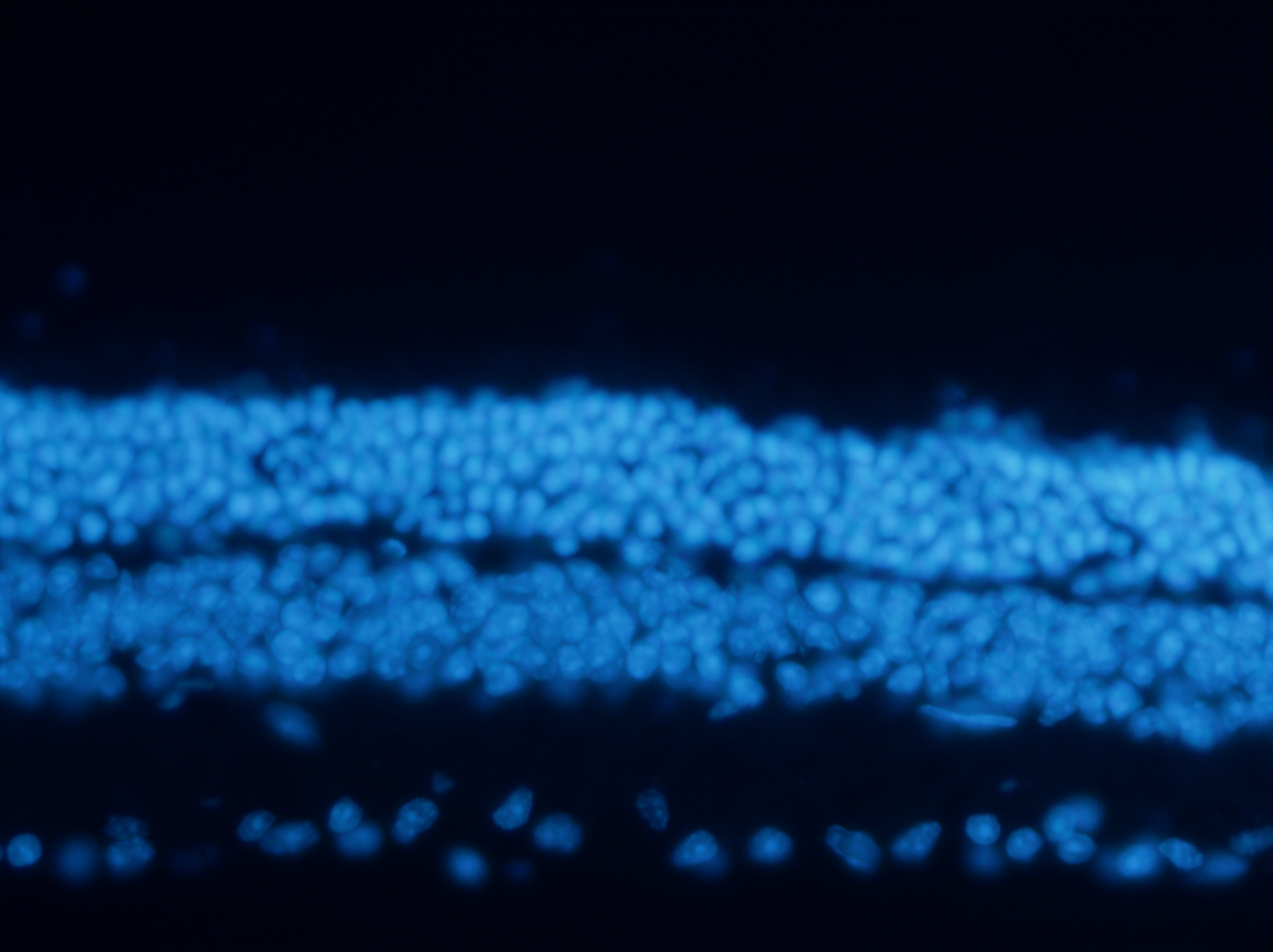

Supplement: Supplementary file 5 — Source Data Fig. 4 [file 44321_2024_53_MOESM5_ESM.zip › Figure 4/4B/DAPI.tif]

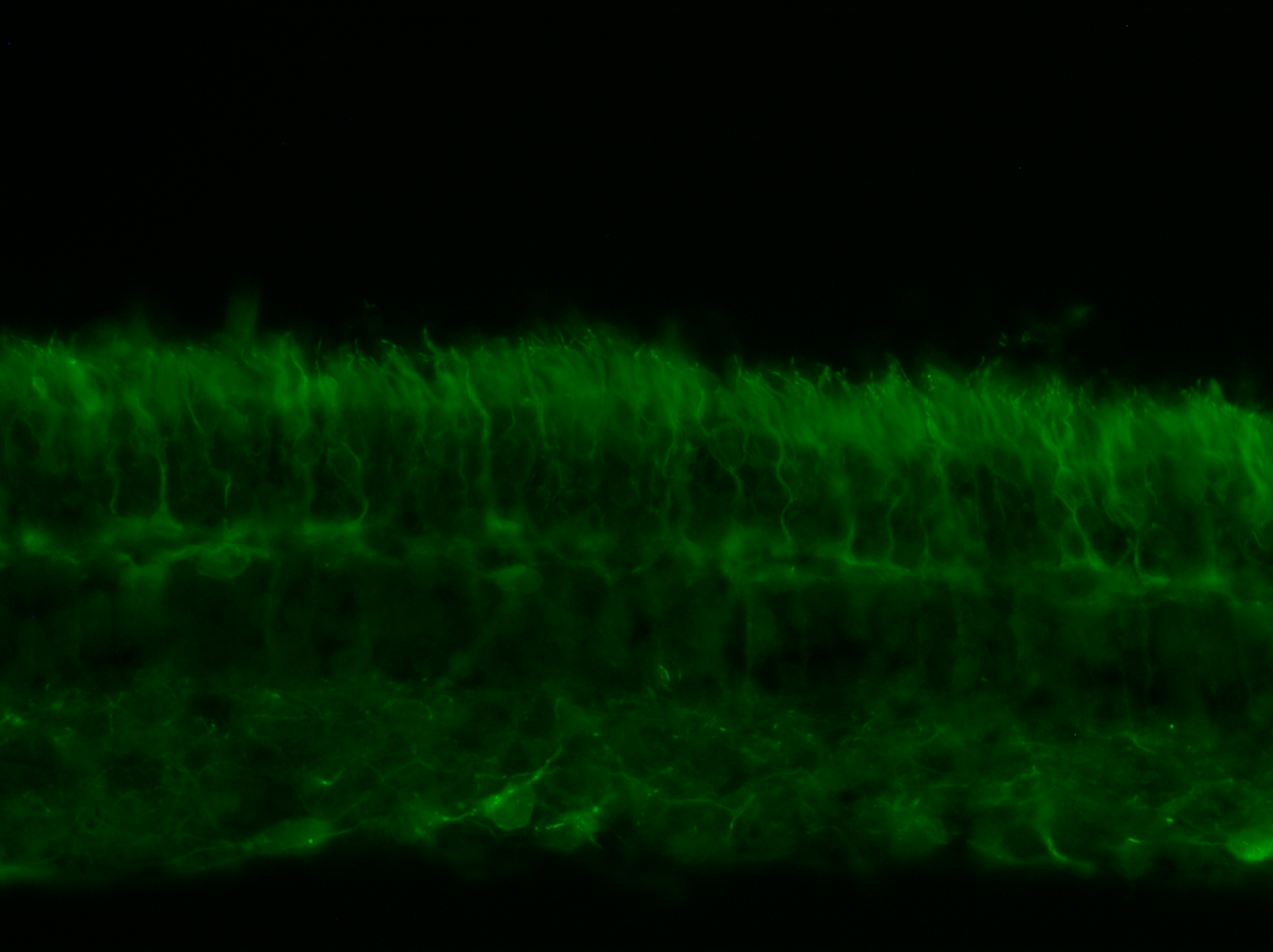

Supplement: Supplementary file 5 — Source Data Fig. 4 [file 44321_2024_53_MOESM5_ESM.zip › Figure 4/4B/FAM161A.tif]

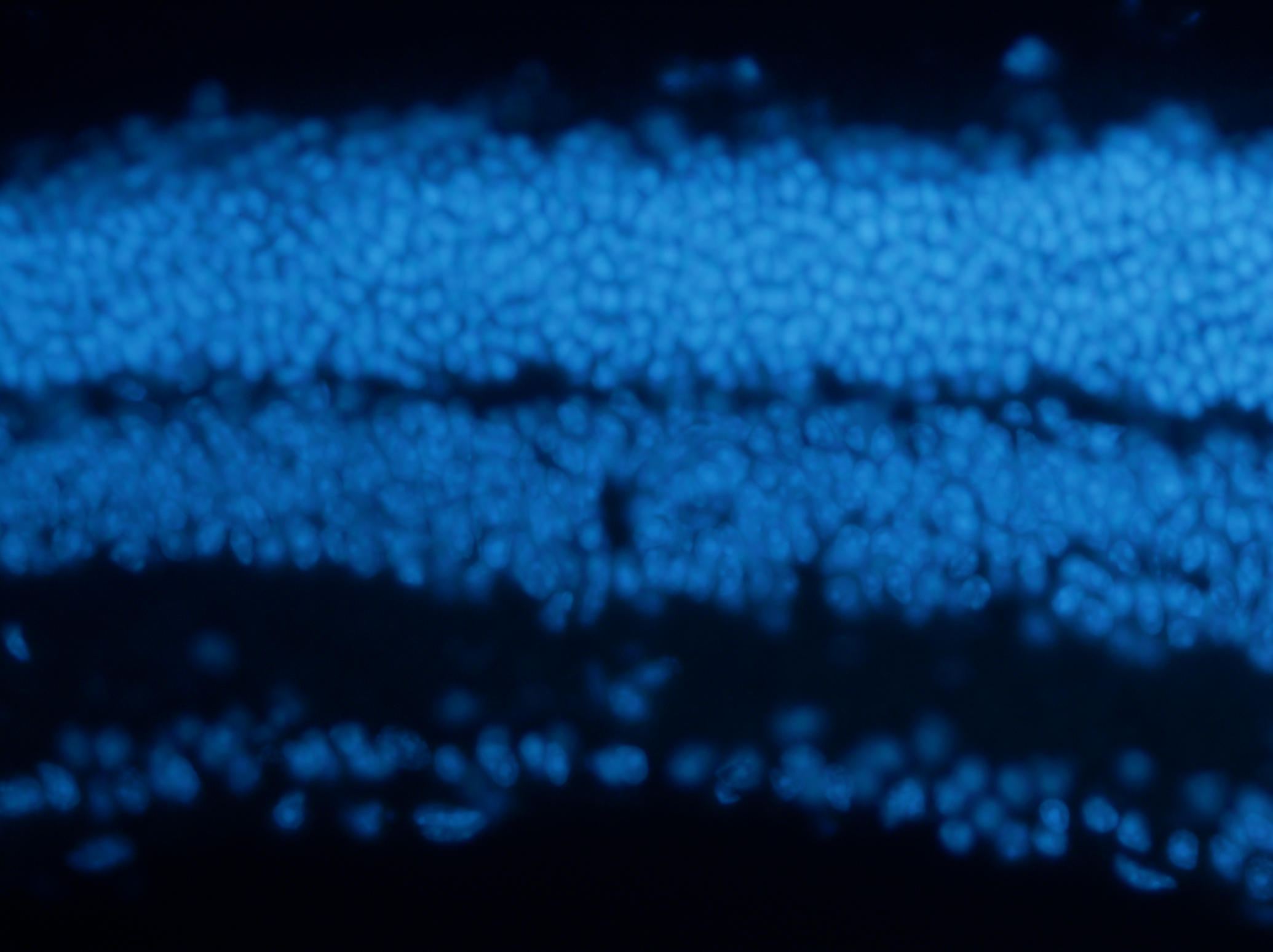

Supplement: Supplementary file 5 — Source Data Fig. 4 [file 44321_2024_53_MOESM5_ESM.zip › Figure 4/4C/DAPI.tif]

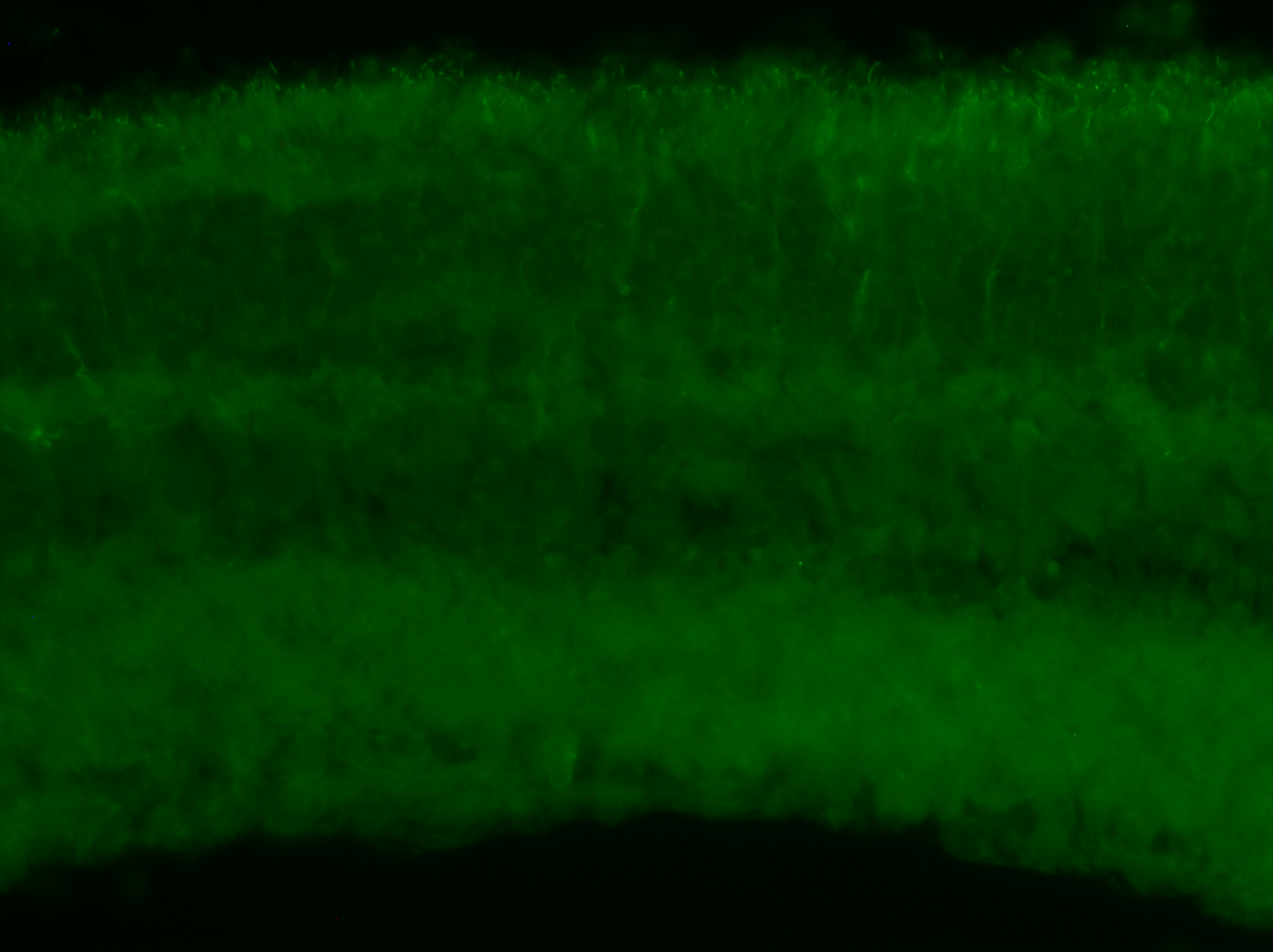

Supplement: Supplementary file 5 — Source Data Fig. 4 [file 44321_2024_53_MOESM5_ESM.zip › Figure 4/4C/FAM161A.tif]

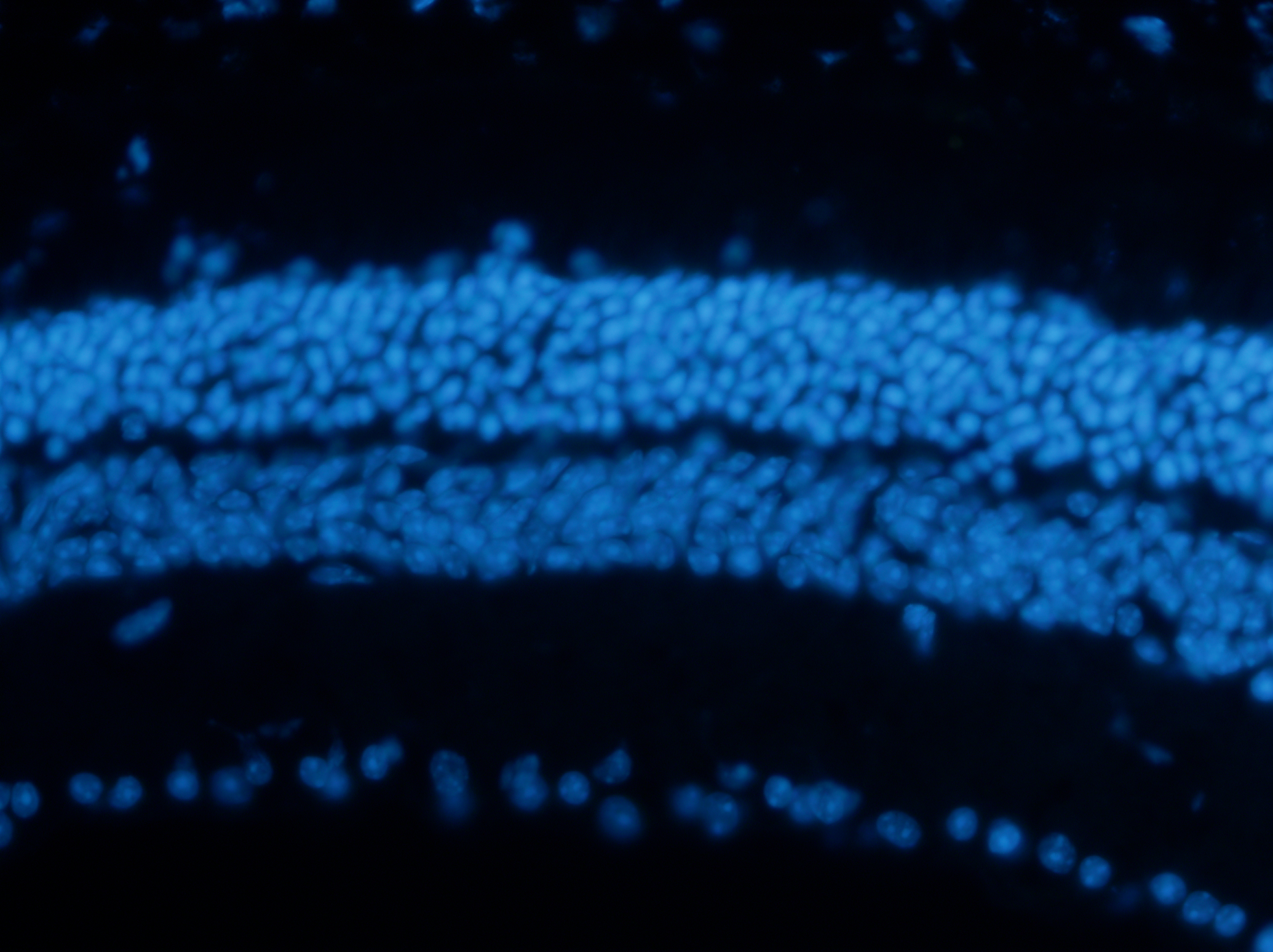

Supplement: Supplementary file 5 — Source Data Fig. 4 [file 44321_2024_53_MOESM5_ESM.zip › Figure 4/4D/DAPI.tif]

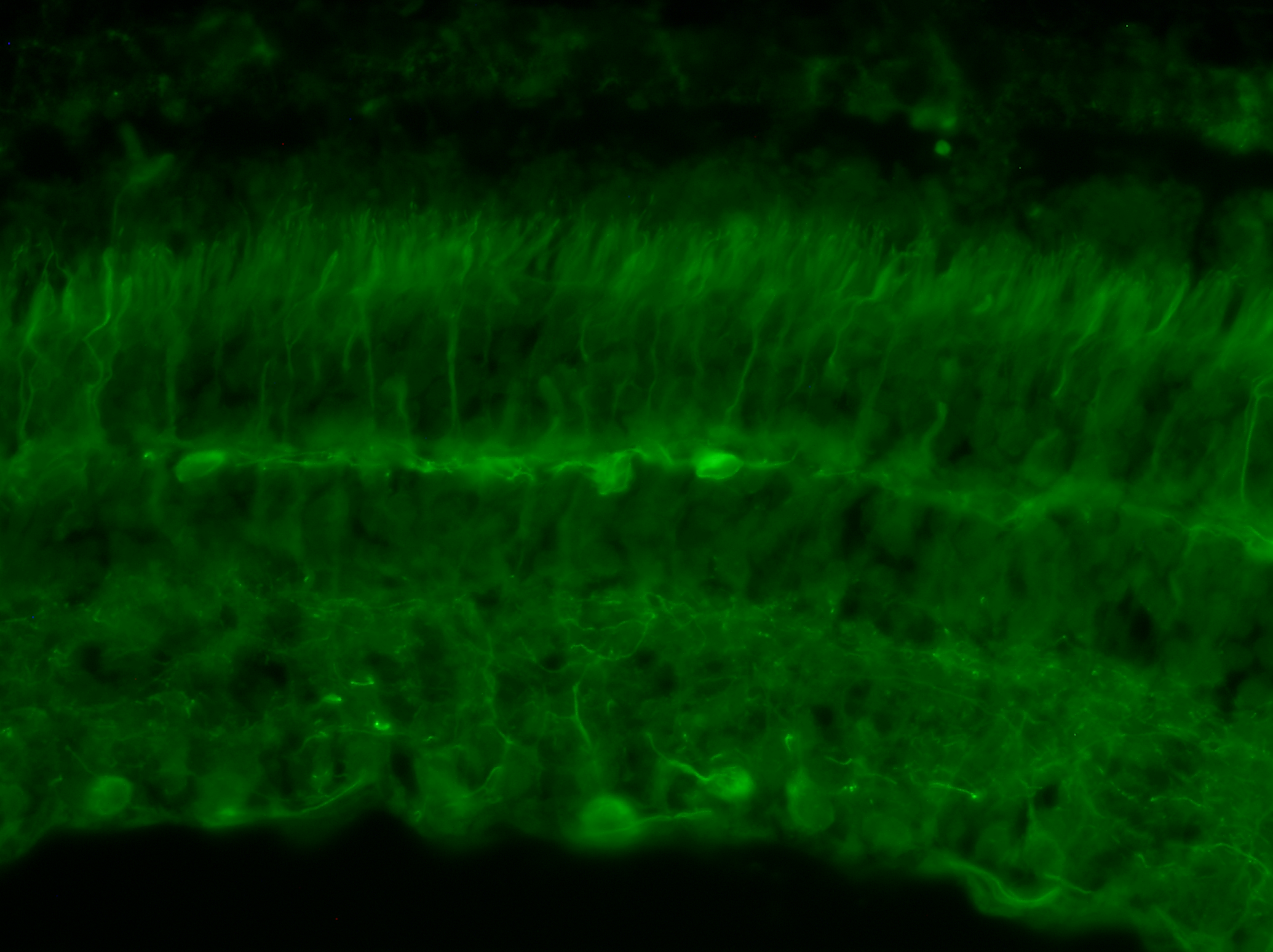

Supplement: Supplementary file 5 — Source Data Fig. 4 [file 44321_2024_53_MOESM5_ESM.zip › Figure 4/4D/FAM161A.tif]

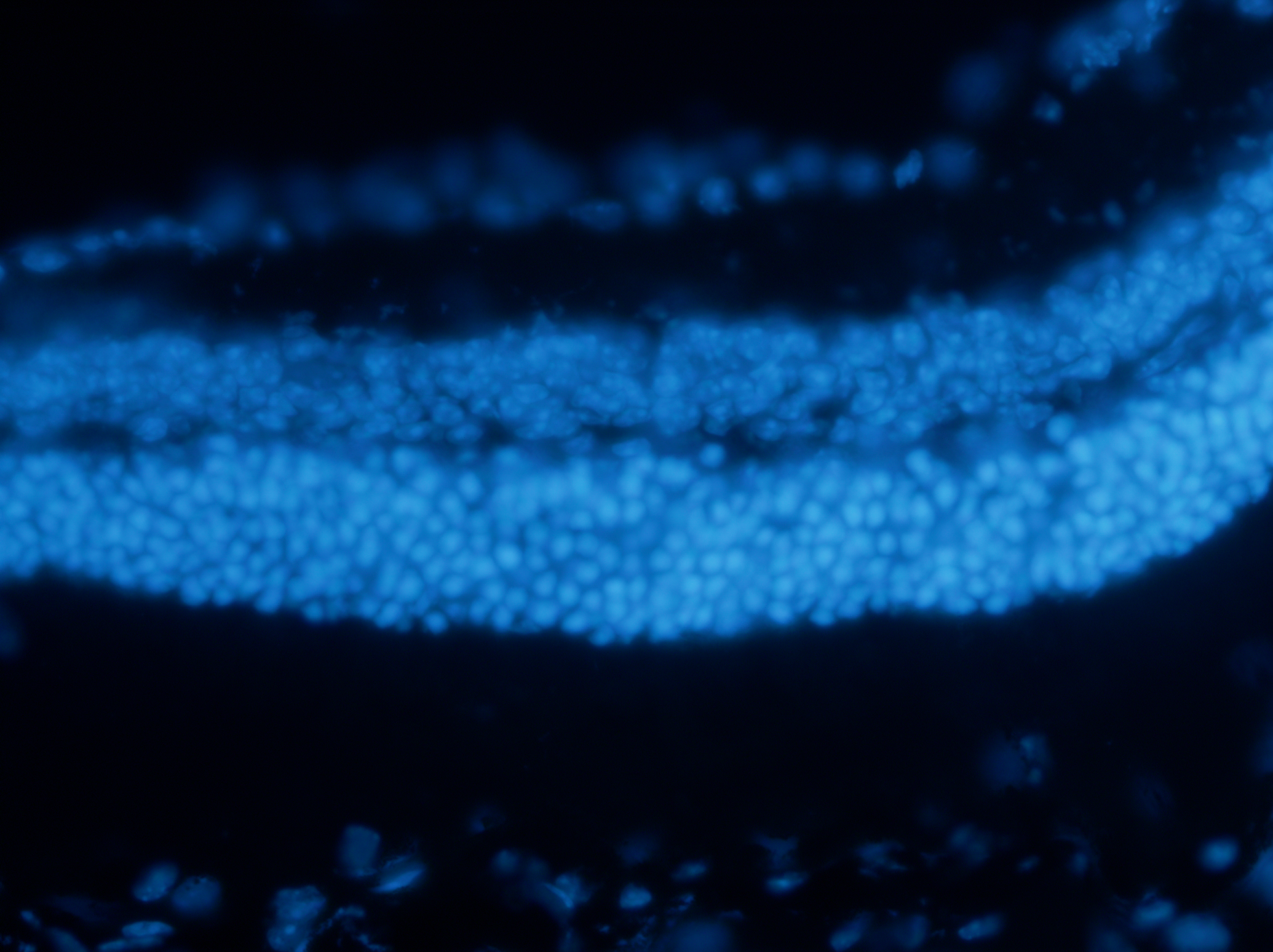

Supplement: Supplementary file 5 — Source Data Fig. 4 [file 44321_2024_53_MOESM5_ESM.zip › Figure 4/4E/DAPI.tif]

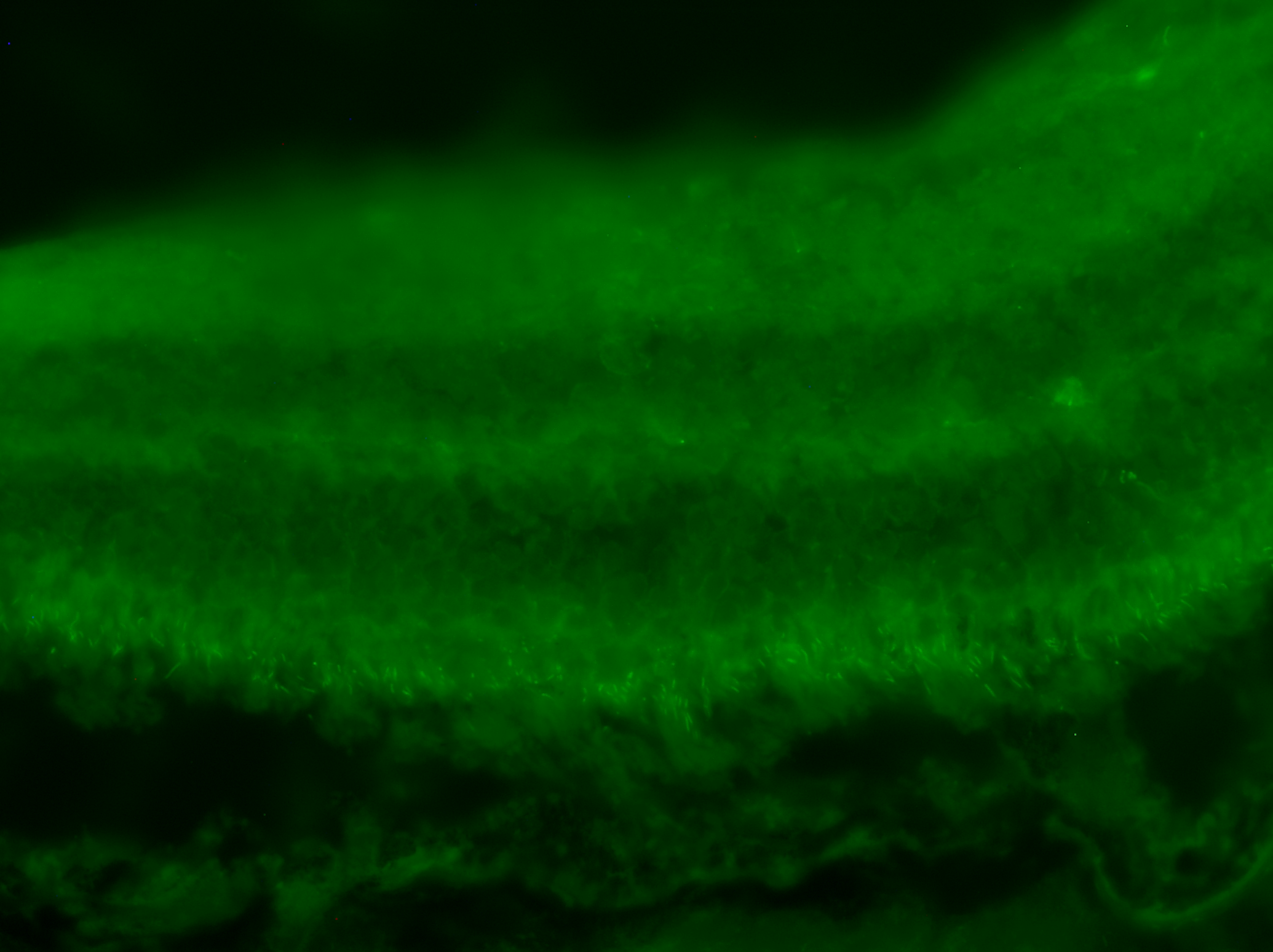

Supplement: Supplementary file 5 — Source Data Fig. 4 [file 44321_2024_53_MOESM5_ESM.zip › Figure 4/4E/FAM161A.tif]

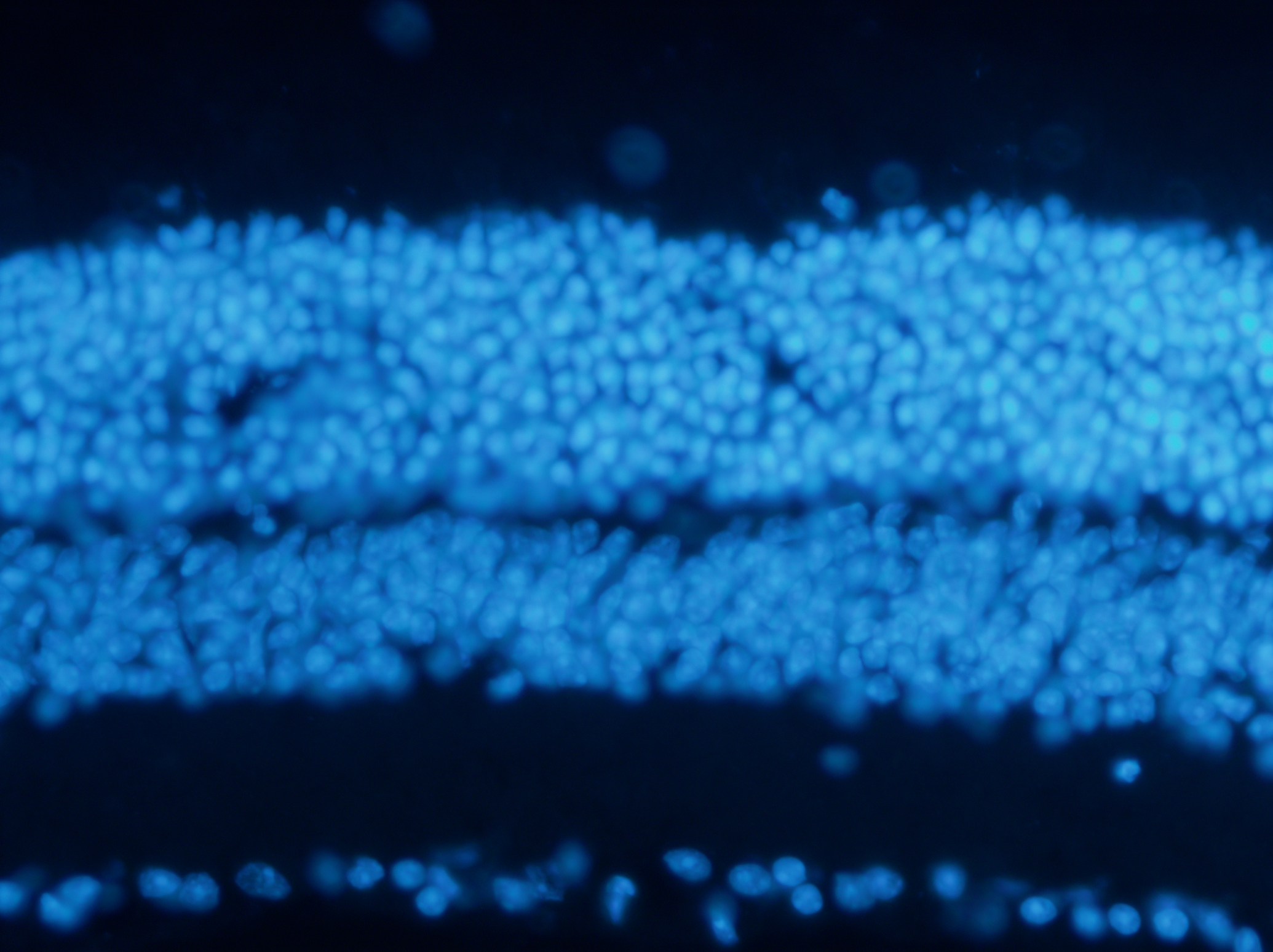

Supplement: Supplementary file 5 — Source Data Fig. 4 [file 44321_2024_53_MOESM5_ESM.zip › Figure 4/4F/DAPI.tif]

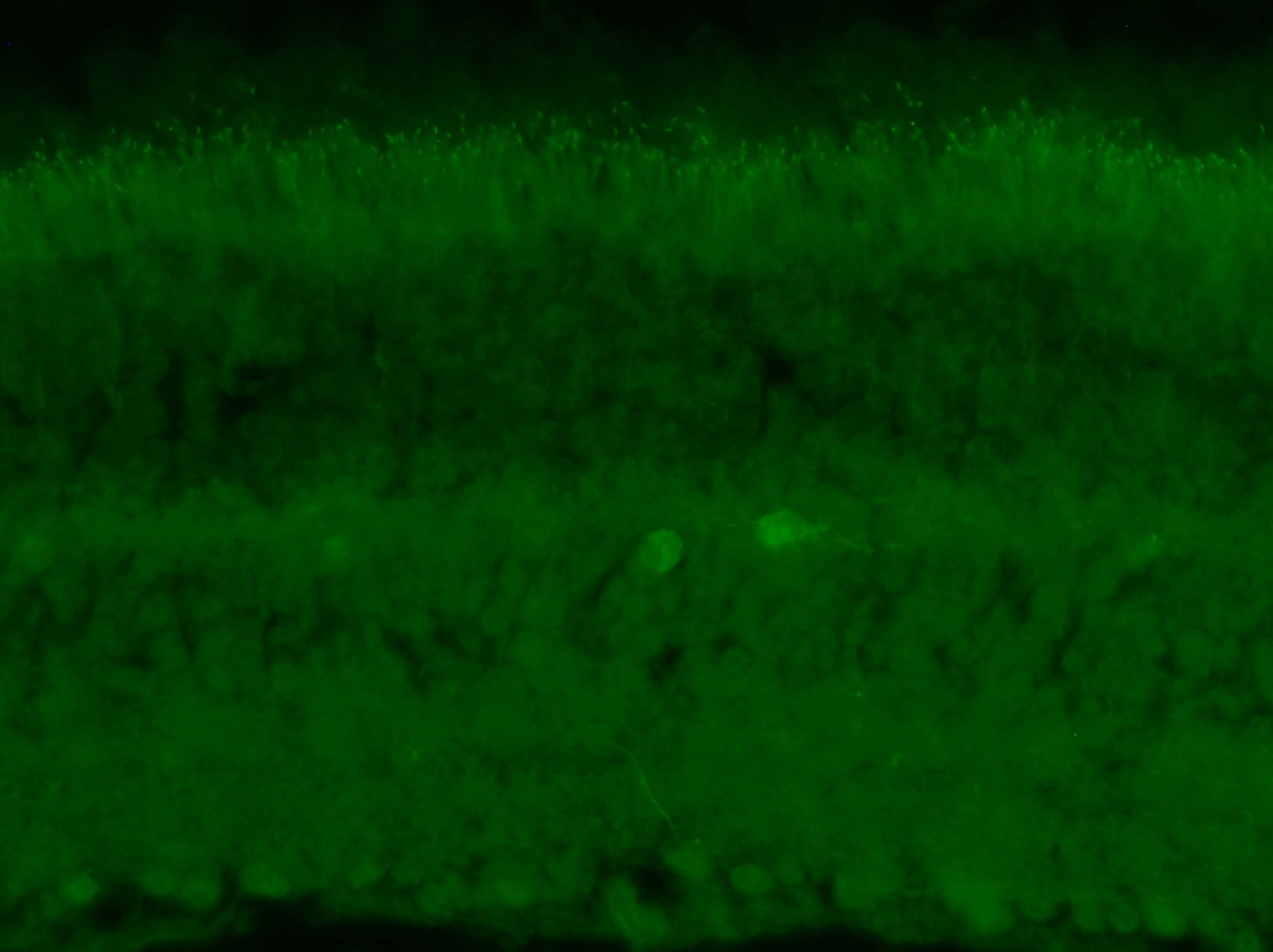

Supplement: Supplementary file 5 — Source Data Fig. 4 [file 44321_2024_53_MOESM5_ESM.zip › Figure 4/4F/FAM161A.tif]

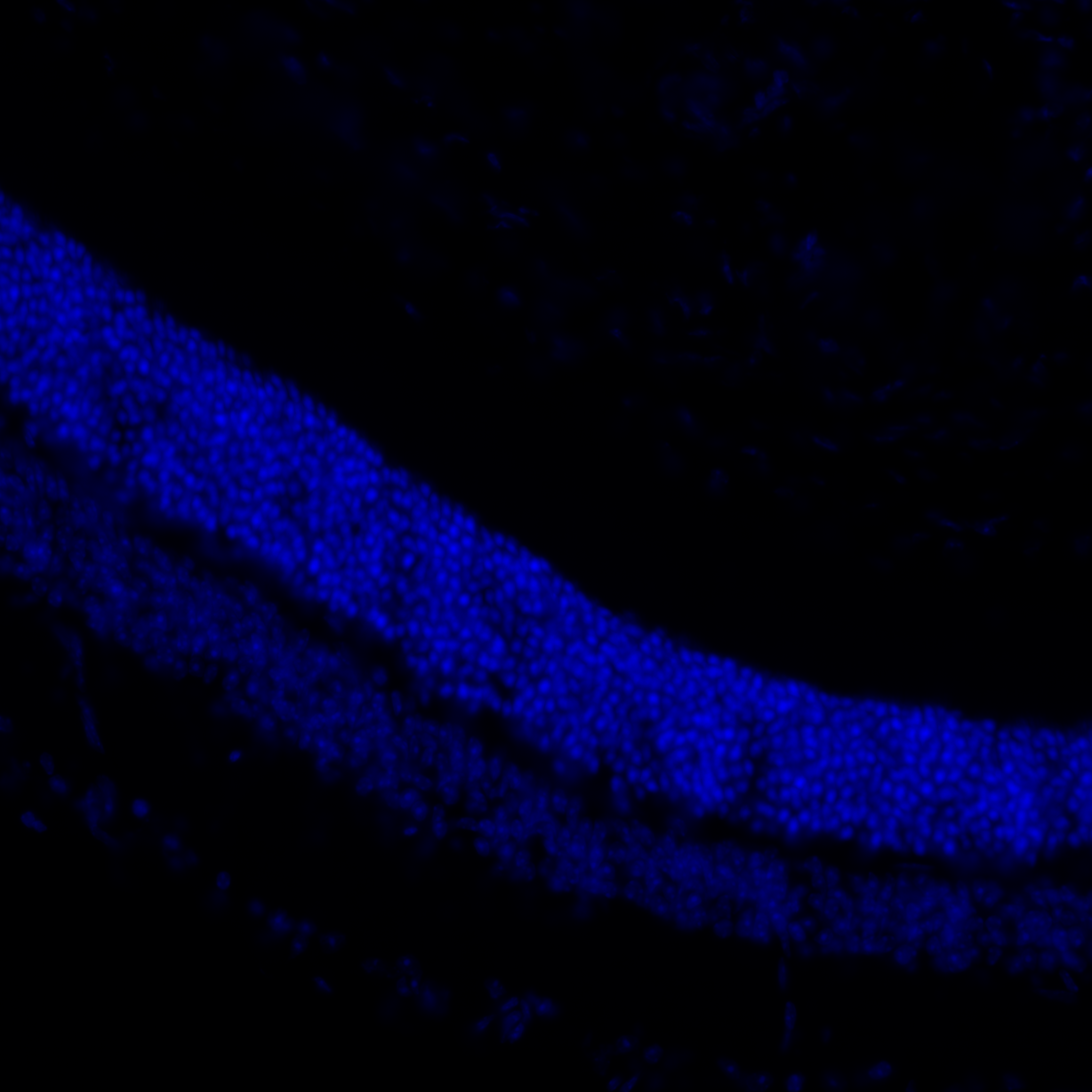

Supplement: Supplementary file 5 — Source Data Fig. 4 [file 44321_2024_53_MOESM5_ESM.zip › Figure 4/4G/DAPI.tif]

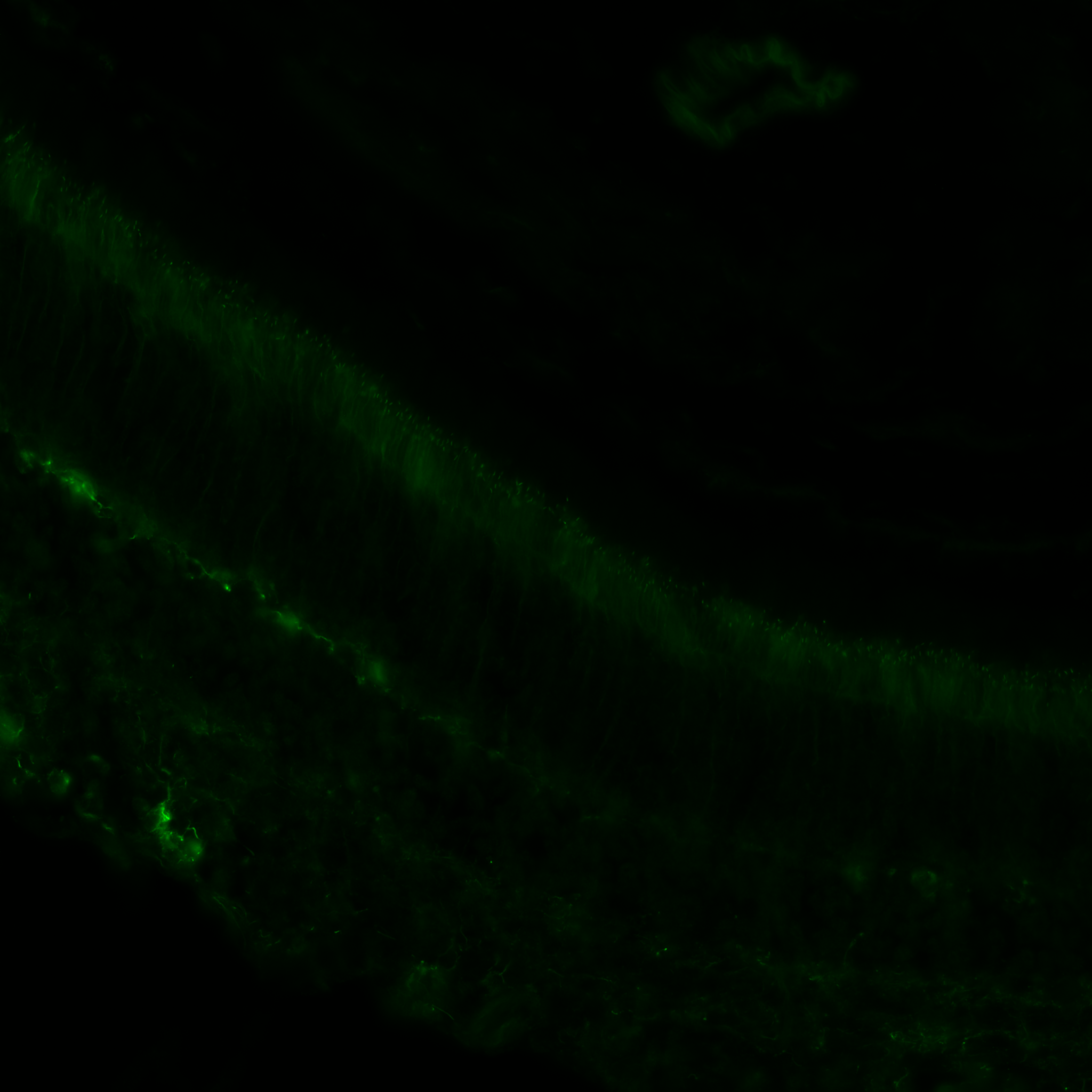

Supplement: Supplementary file 5 — Source Data Fig. 4 [file 44321_2024_53_MOESM5_ESM.zip › Figure 4/4G/FAM161A.tif]

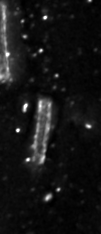

Supplement: Supplementary file 6 — Source Data Fig. 5 [file 44321_2024_53_MOESM6_ESM.zip › Figure 5/A/cep290.tif]

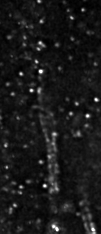

Supplement: Supplementary file 6 — Source Data Fig. 5 [file 44321_2024_53_MOESM6_ESM.zip › Figure 5/A/Fam161a.tif]

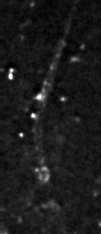

Supplement: Supplementary file 6 — Source Data Fig. 5 [file 44321_2024_53_MOESM6_ESM.zip › Figure 5/A/IFT81.tif]

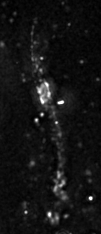

Supplement: Supplementary file 6 — Source Data Fig. 5 [file 44321_2024_53_MOESM6_ESM.zip › Figure 5/A/lca5.tif]

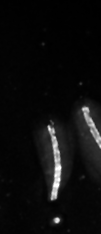

Supplement: Supplementary file 6 — Source Data Fig. 5 [file 44321_2024_53_MOESM6_ESM.zip › Figure 5/A/POC5.tif]

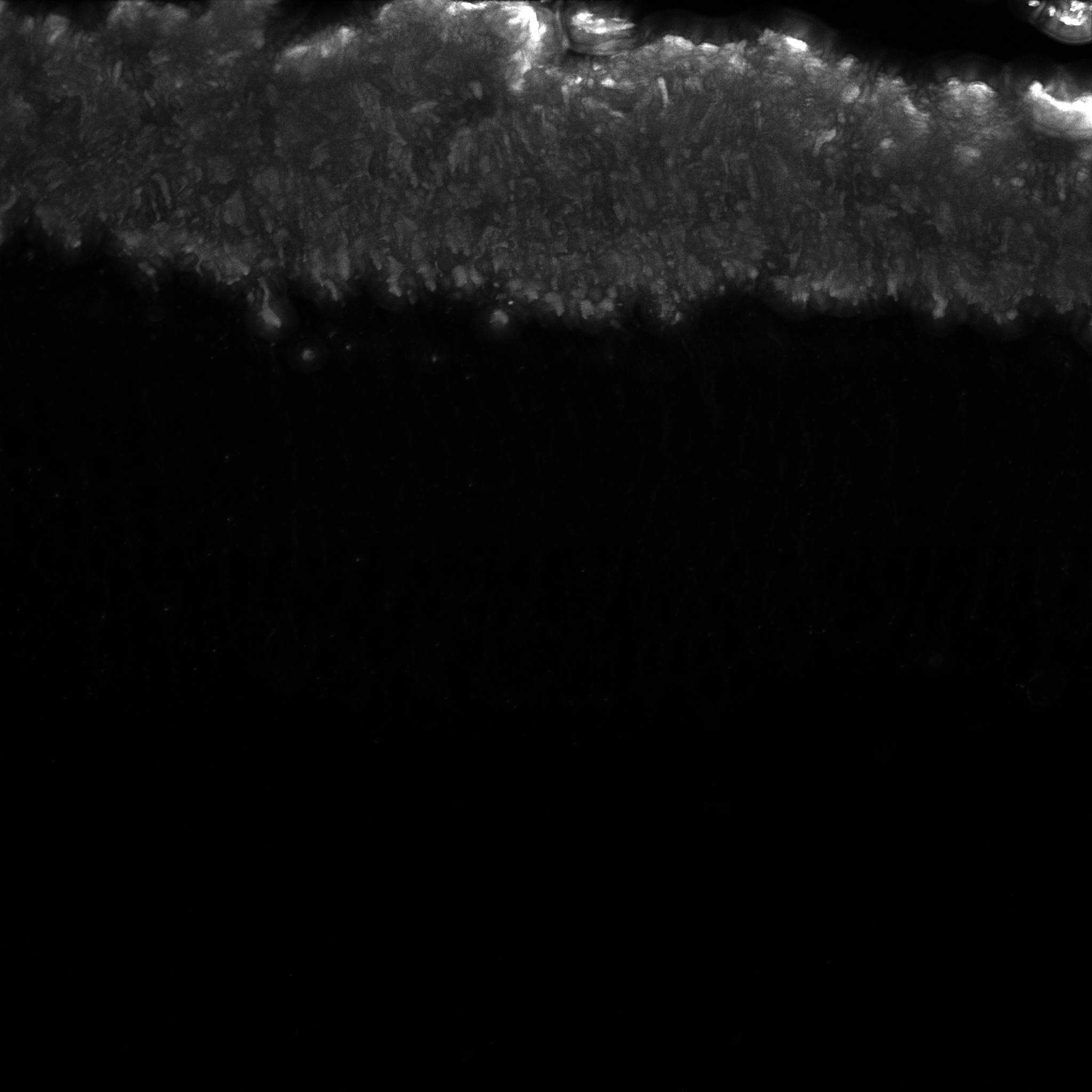

Supplement: Supplementary file 6 — Source Data Fig. 5 [file 44321_2024_53_MOESM6_ESM.zip › Figure 5/A/Rhodopsin.tif]

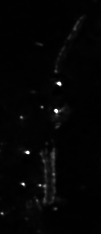

Supplement: Supplementary file 6 — Source Data Fig. 5 [file 44321_2024_53_MOESM6_ESM.zip › Figure 5/B/Cep290.tif]

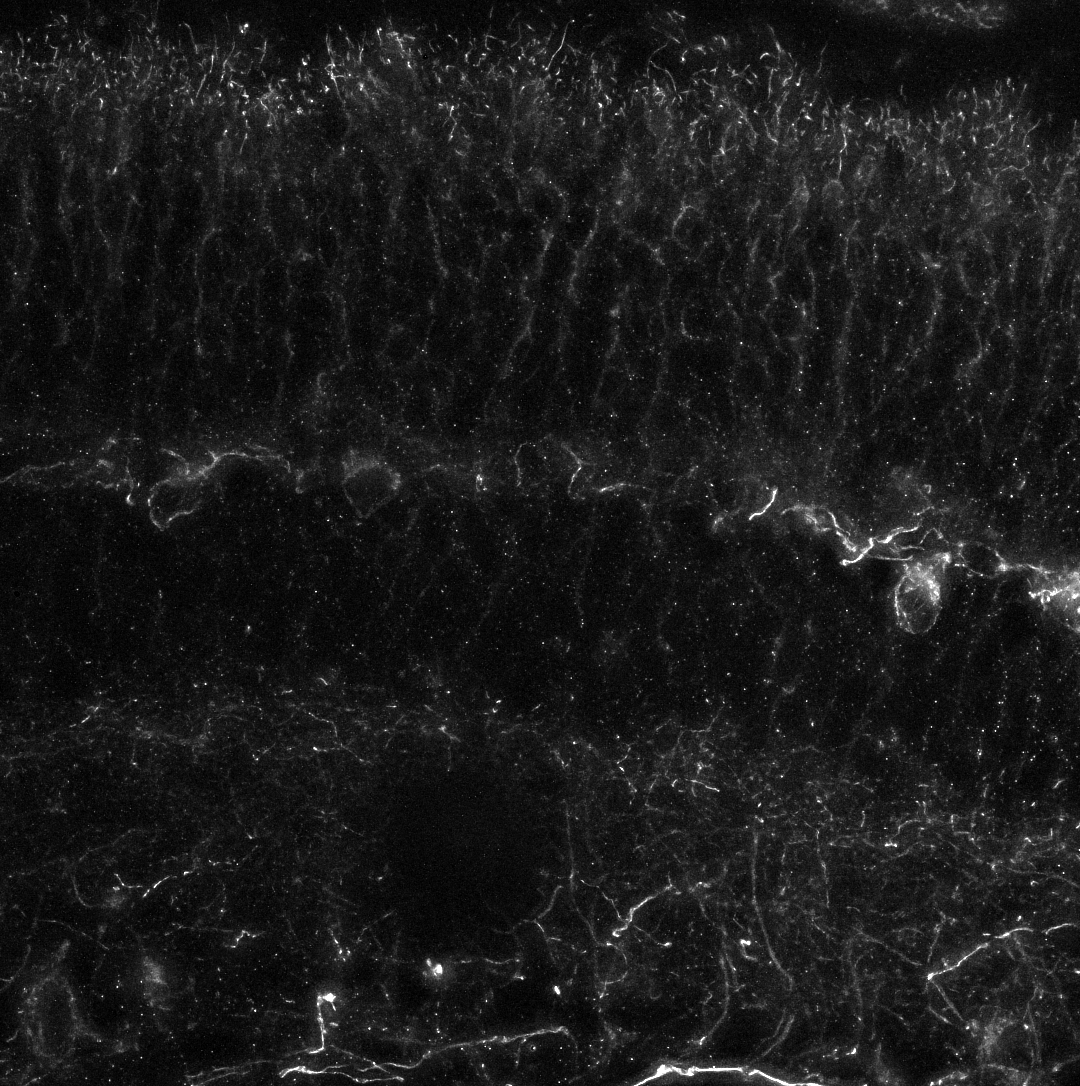

Supplement: Supplementary file 6 — Source Data Fig. 5 [file 44321_2024_53_MOESM6_ESM.zip › Figure 5/B/Fam161a low mag.tif]

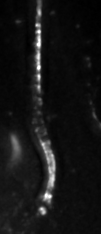

Supplement: Supplementary file 6 — Source Data Fig. 5 [file 44321_2024_53_MOESM6_ESM.zip › Figure 5/B/Fam161a.tif]

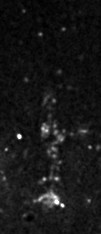

Supplement: Supplementary file 6 — Source Data Fig. 5 [file 44321_2024_53_MOESM6_ESM.zip › Figure 5/B/Ift81.tif]

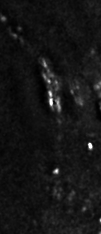

Supplement: Supplementary file 6 — Source Data Fig. 5 [file 44321_2024_53_MOESM6_ESM.zip › Figure 5/B/Lca5.tif]

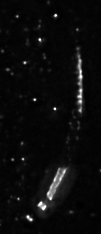

Supplement: Supplementary file 6 — Source Data Fig. 5 [file 44321_2024_53_MOESM6_ESM.zip › Figure 5/B/Poc5.tif]

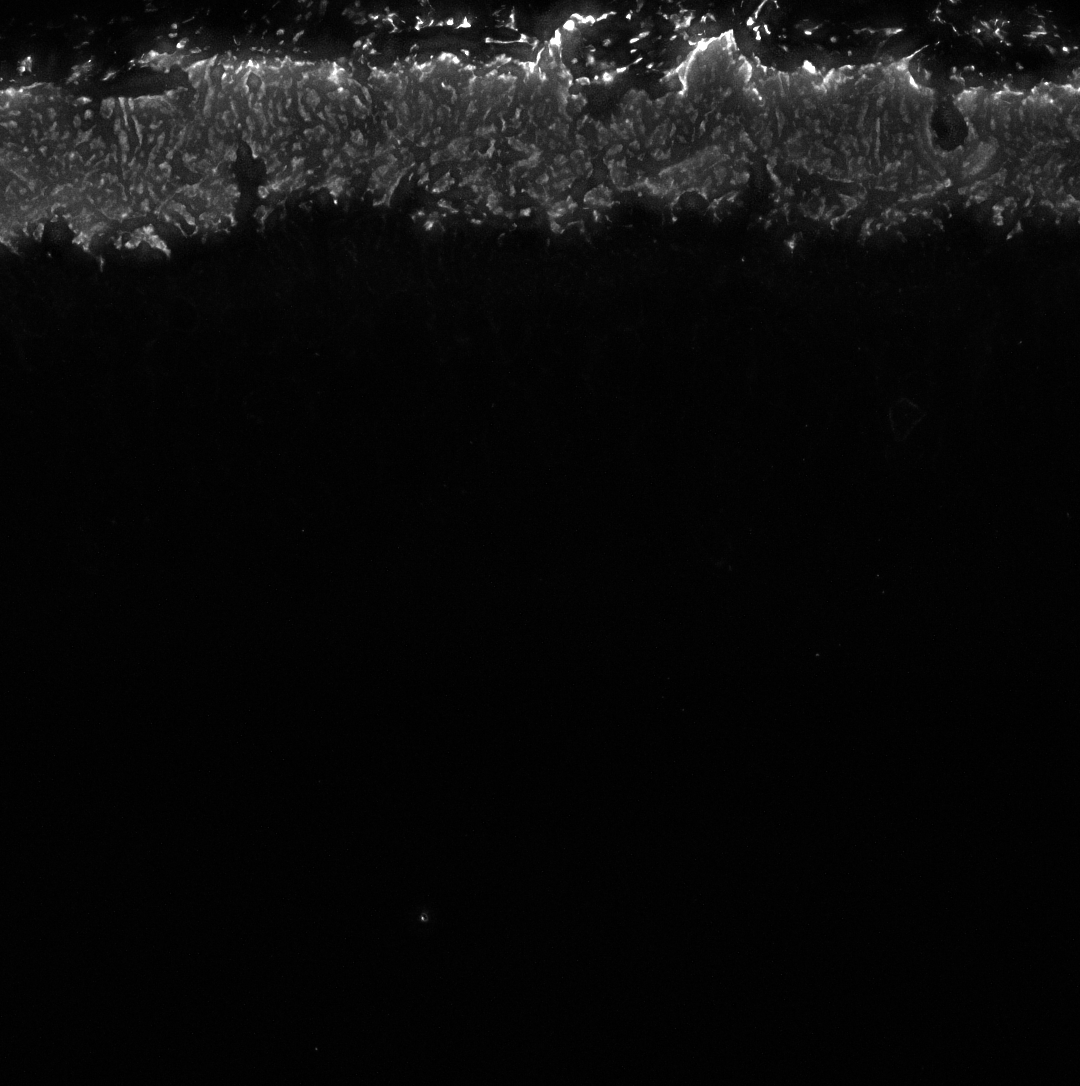

Supplement: Supplementary file 6 — Source Data Fig. 5 [file 44321_2024_53_MOESM6_ESM.zip › Figure 5/B/Rhodopsin.tif]

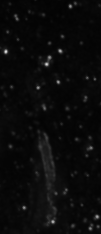

Supplement: Supplementary file 6 — Source Data Fig. 5 [file 44321_2024_53_MOESM6_ESM.zip › Figure 5/C/CEP290.tif]

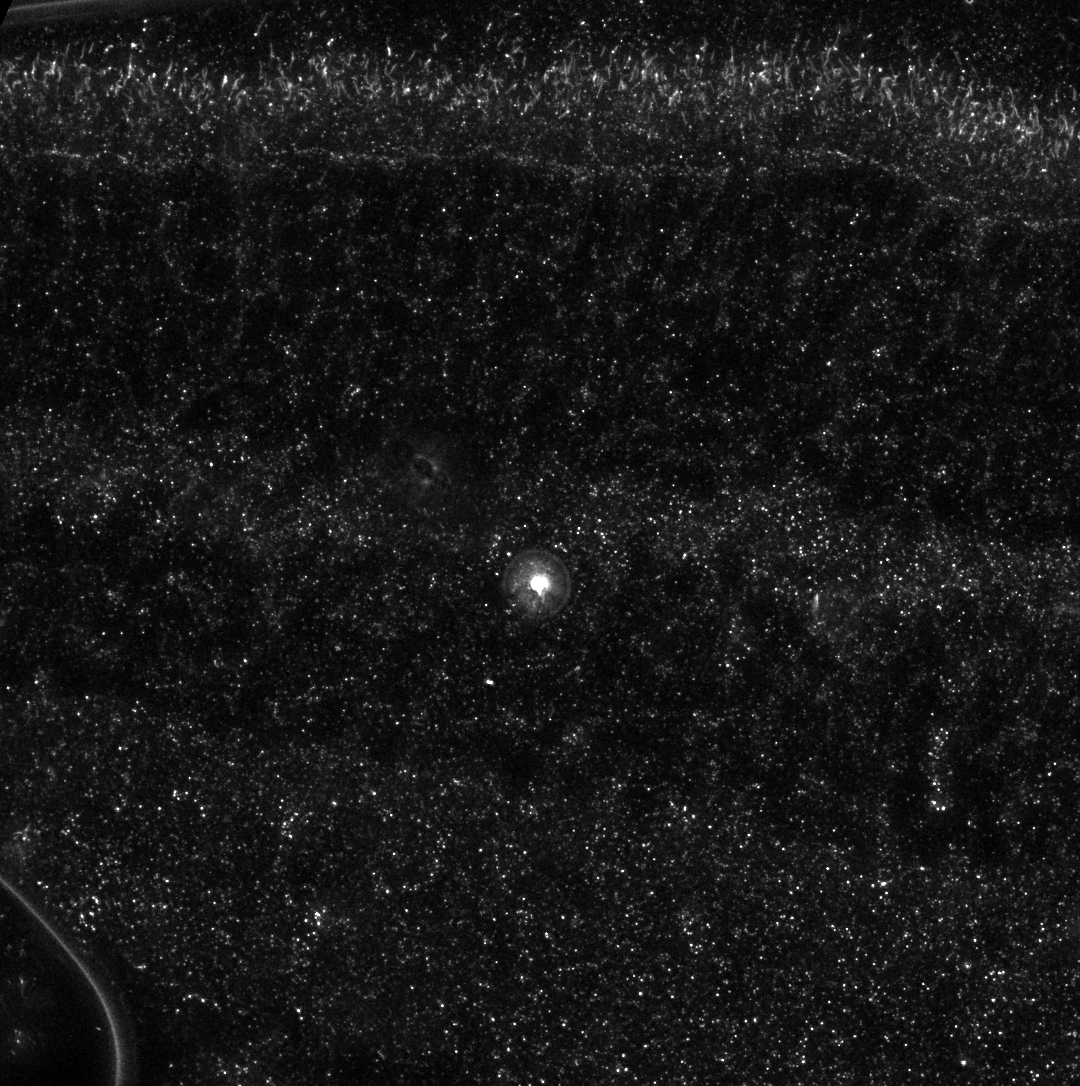

Supplement: Supplementary file 6 — Source Data Fig. 5 [file 44321_2024_53_MOESM6_ESM.zip › Figure 5/C/Fam161a low mag.tif]

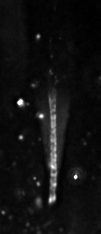

Supplement: Supplementary file 6 — Source Data Fig. 5 [file 44321_2024_53_MOESM6_ESM.zip › Figure 5/C/Fam161a.tif]

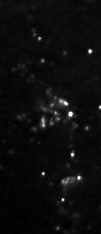

Supplement: Supplementary file 6 — Source Data Fig. 5 [file 44321_2024_53_MOESM6_ESM.zip › Figure 5/C/IFT81.tif]

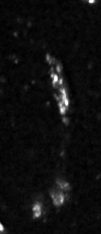

Supplement: Supplementary file 6 — Source Data Fig. 5 [file 44321_2024_53_MOESM6_ESM.zip › Figure 5/C/Lca5.tif]

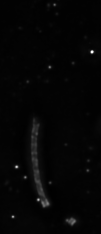

Supplement: Supplementary file 6 — Source Data Fig. 5 [file 44321_2024_53_MOESM6_ESM.zip › Figure 5/C/POC5.tif]

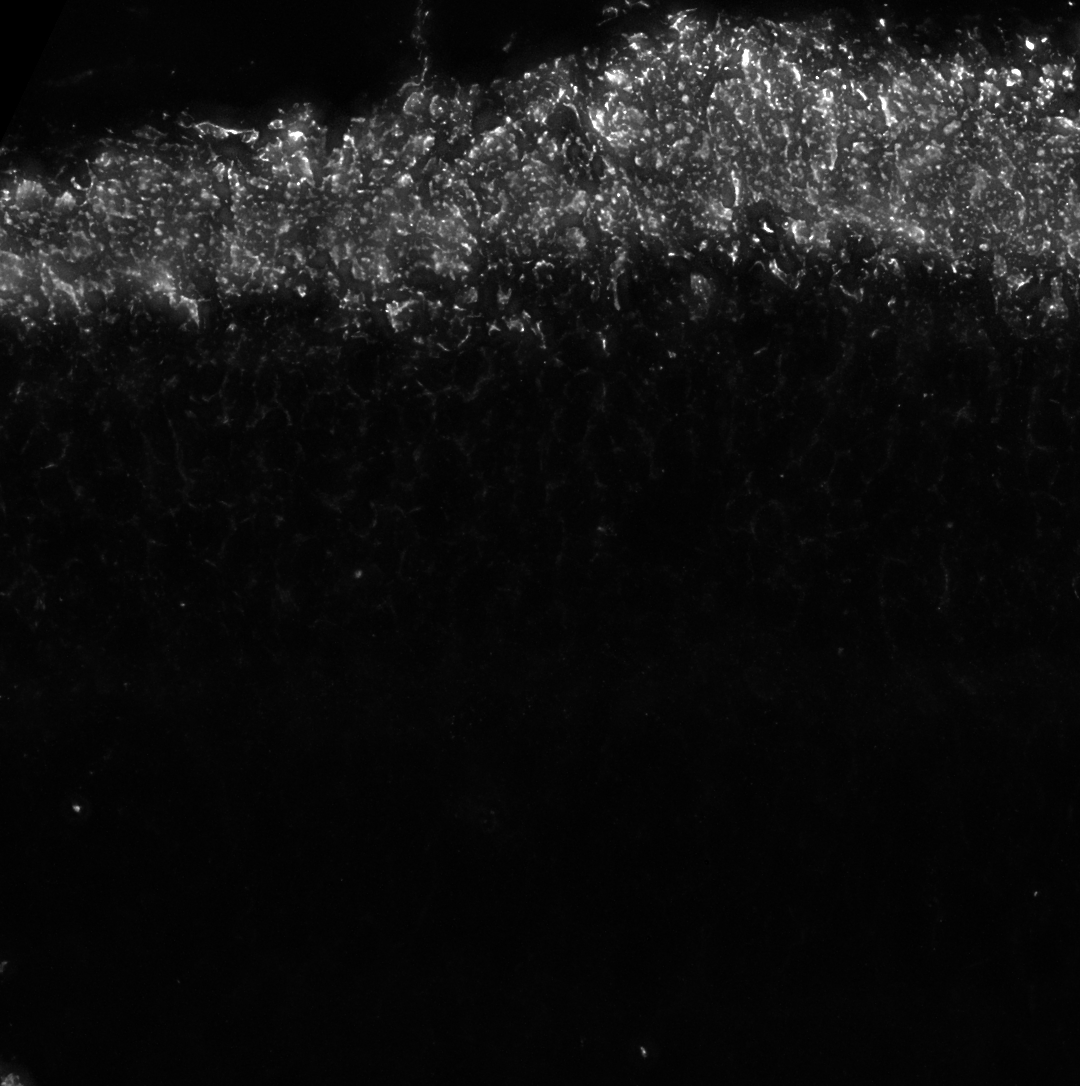

Supplement: Supplementary file 6 — Source Data Fig. 5 [file 44321_2024_53_MOESM6_ESM.zip › Figure 5/C/Rhodopsin.tif]

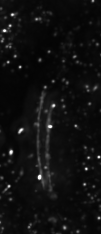

Supplement: Supplementary file 6 — Source Data Fig. 5 [file 44321_2024_53_MOESM6_ESM.zip › Figure 5/D/CEP290.tif]

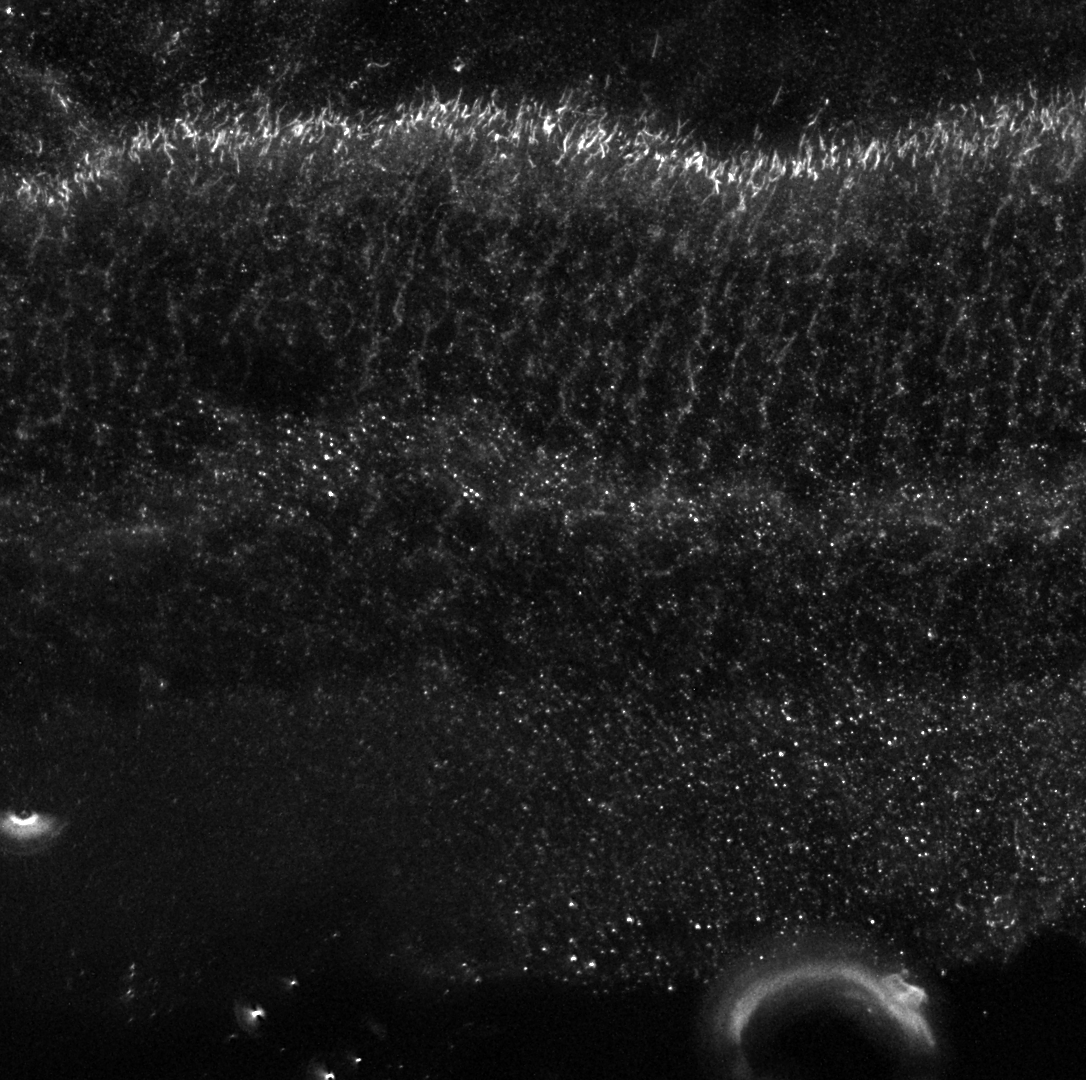

Supplement: Supplementary file 6 — Source Data Fig. 5 [file 44321_2024_53_MOESM6_ESM.zip › Figure 5/D/FAM161A low mag.tif]

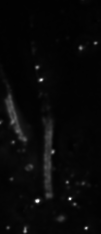

Supplement: Supplementary file 6 — Source Data Fig. 5 [file 44321_2024_53_MOESM6_ESM.zip › Figure 5/D/Fam161a.tif]

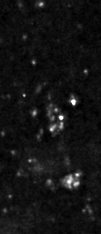

Supplement: Supplementary file 6 — Source Data Fig. 5 [file 44321_2024_53_MOESM6_ESM.zip › Figure 5/D/IFT81.tif]

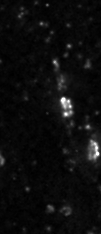

Supplement: Supplementary file 6 — Source Data Fig. 5 [file 44321_2024_53_MOESM6_ESM.zip › Figure 5/D/LCA5.tif]

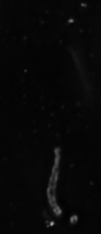

Supplement: Supplementary file 6 — Source Data Fig. 5 [file 44321_2024_53_MOESM6_ESM.zip › Figure 5/D/POC5.tif]

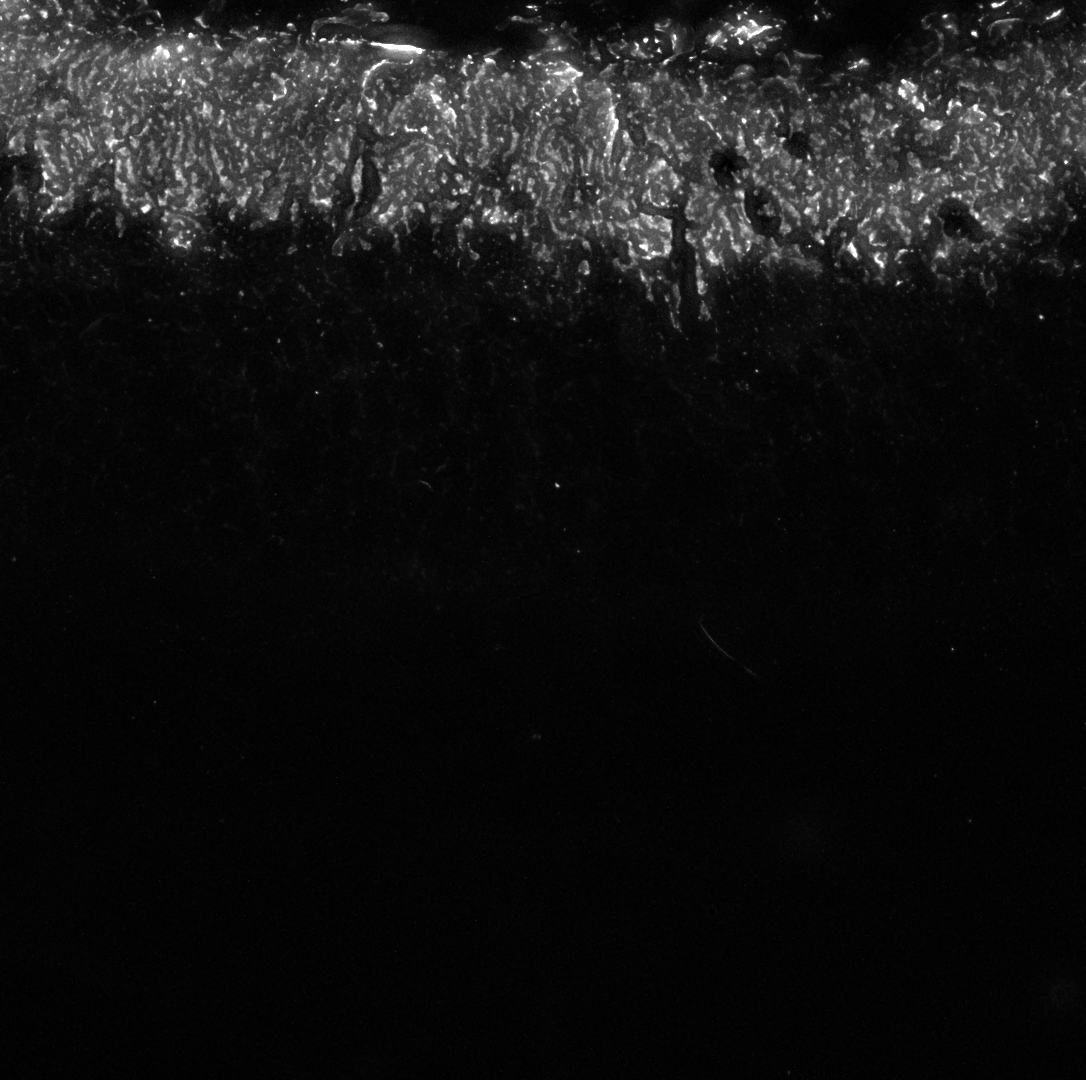

Supplement: Supplementary file 6 — Source Data Fig. 5 [file 44321_2024_53_MOESM6_ESM.zip › Figure 5/D/Rhodopsin.tif]

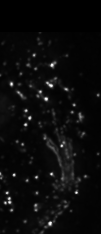

Supplement: Supplementary file 6 — Source Data Fig. 5 [file 44321_2024_53_MOESM6_ESM.zip › Figure 5/E/CEP290.tif]

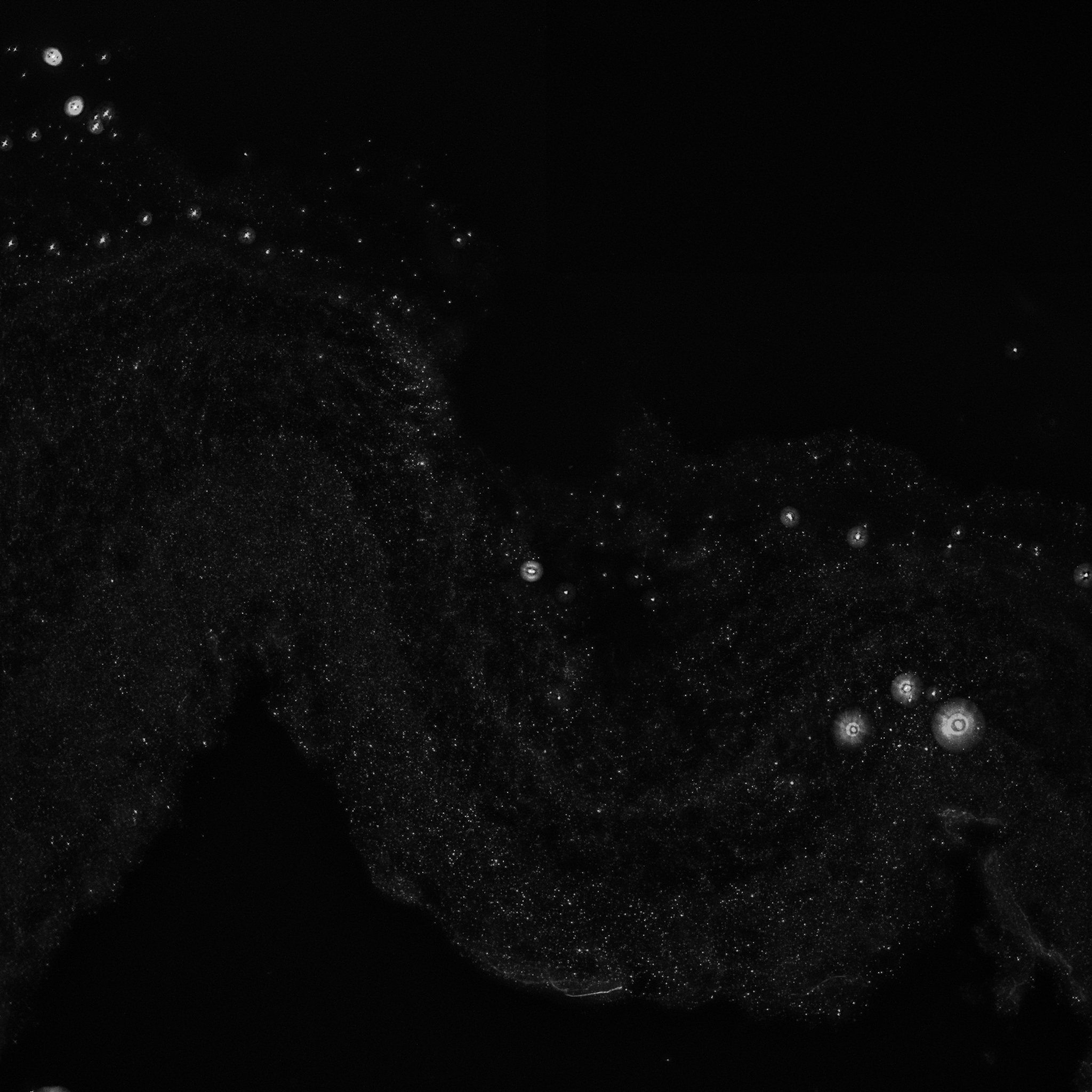

Supplement: Supplementary file 6 — Source Data Fig. 5 [file 44321_2024_53_MOESM6_ESM.zip › Figure 5/E/Fam161a low mag.tif]

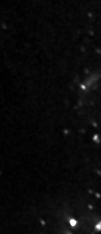

Supplement: Supplementary file 6 — Source Data Fig. 5 [file 44321_2024_53_MOESM6_ESM.zip › Figure 5/E/Fam161a.tif]

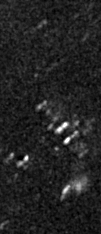

Supplement: Supplementary file 6 — Source Data Fig. 5 [file 44321_2024_53_MOESM6_ESM.zip › Figure 5/E/IFT81.tif]

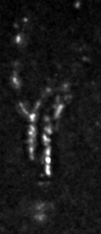

Supplement: Supplementary file 6 — Source Data Fig. 5 [file 44321_2024_53_MOESM6_ESM.zip › Figure 5/E/LCA5.tif]
