# Supplementary figures and images for: Fine-tuning FAM161A gene augmentation therapy to restore retinal function (part 2 of 2)
Source: EMBO Mol Med. 2024 Mar 19;16(4):805–22. doi: 10.1038/s44321-024-00053-x (PMC11018783; doi:10.1038/s44321-024-00053-x)

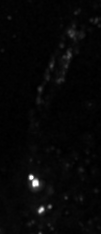

Supplement: Supplementary file 6 — Source Data Fig. 5 [file 44321_2024_53_MOESM6_ESM.zip › Figure 5/E/POC5.tif]

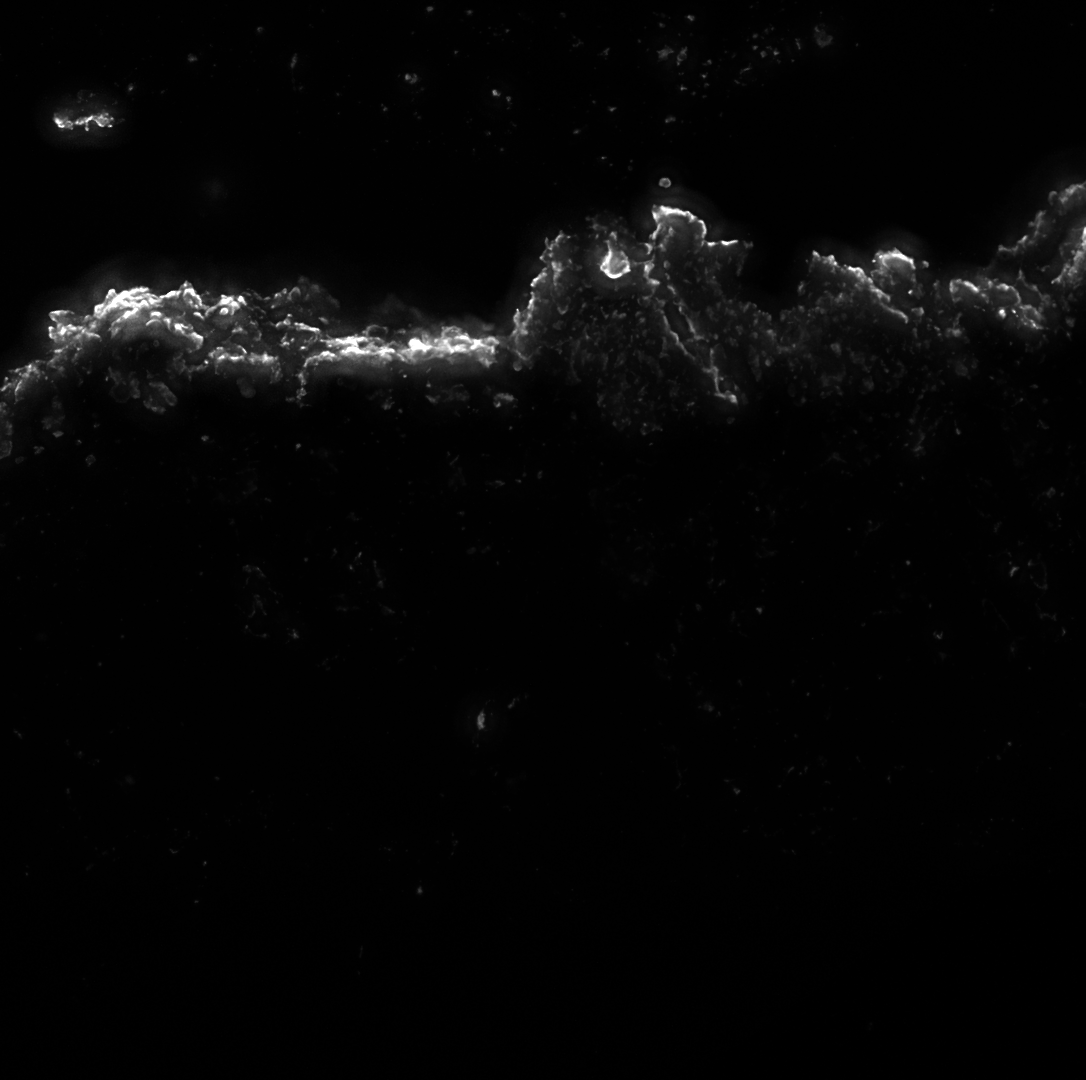

Supplement: Supplementary file 6 — Source Data Fig. 5 [file 44321_2024_53_MOESM6_ESM.zip › Figure 5/E/Rhodopsin.tif]

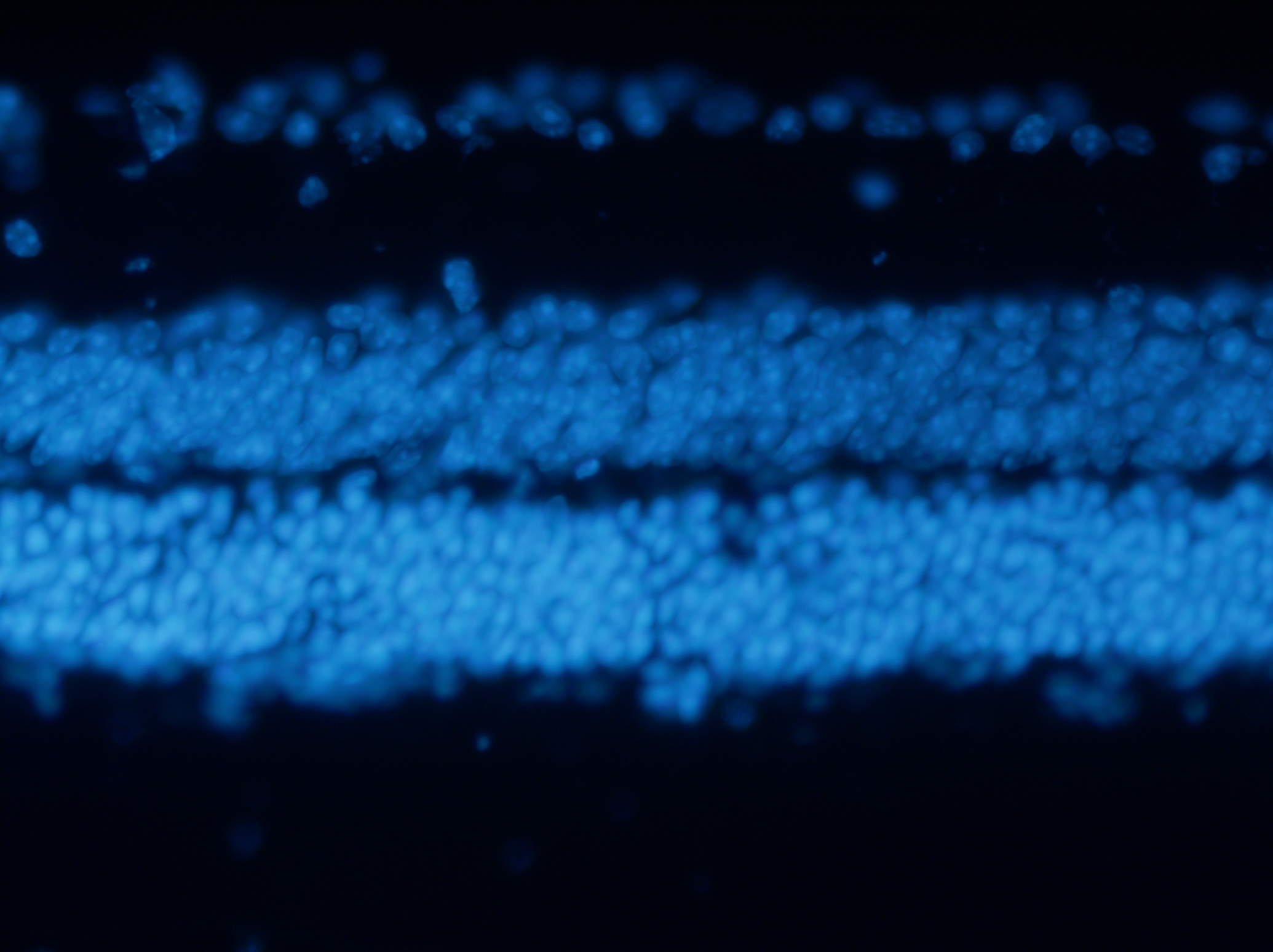

Supplement: Supplementary file 9 — Appendix Source Data [file 44321_2024_53_MOESM9_ESM.zip › Appendix/S2/10e9_DAPI.tif]

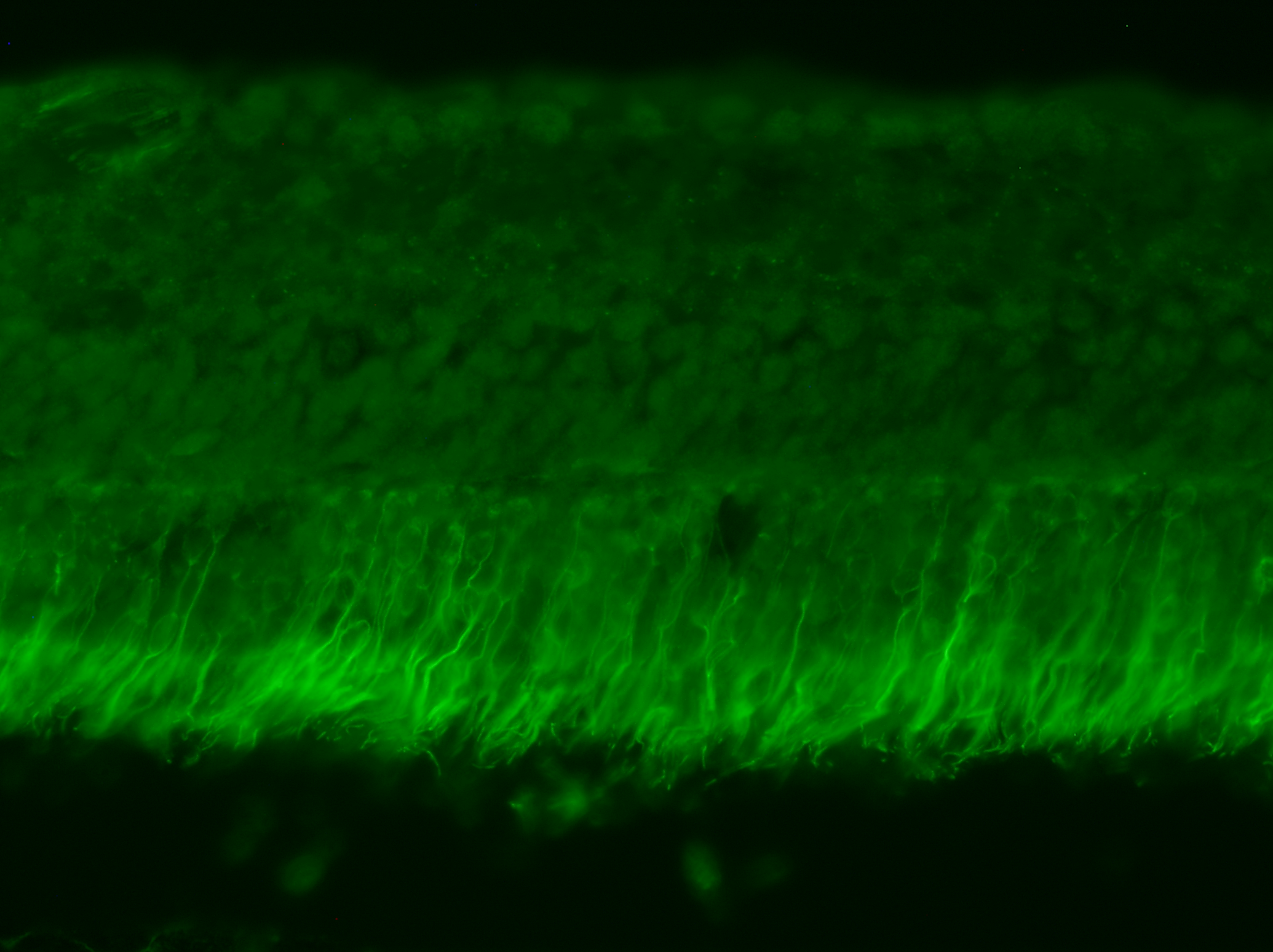

Supplement: Supplementary file 9 — Appendix Source Data [file 44321_2024_53_MOESM9_ESM.zip › Appendix/S2/10e9_FAM161A.tif]

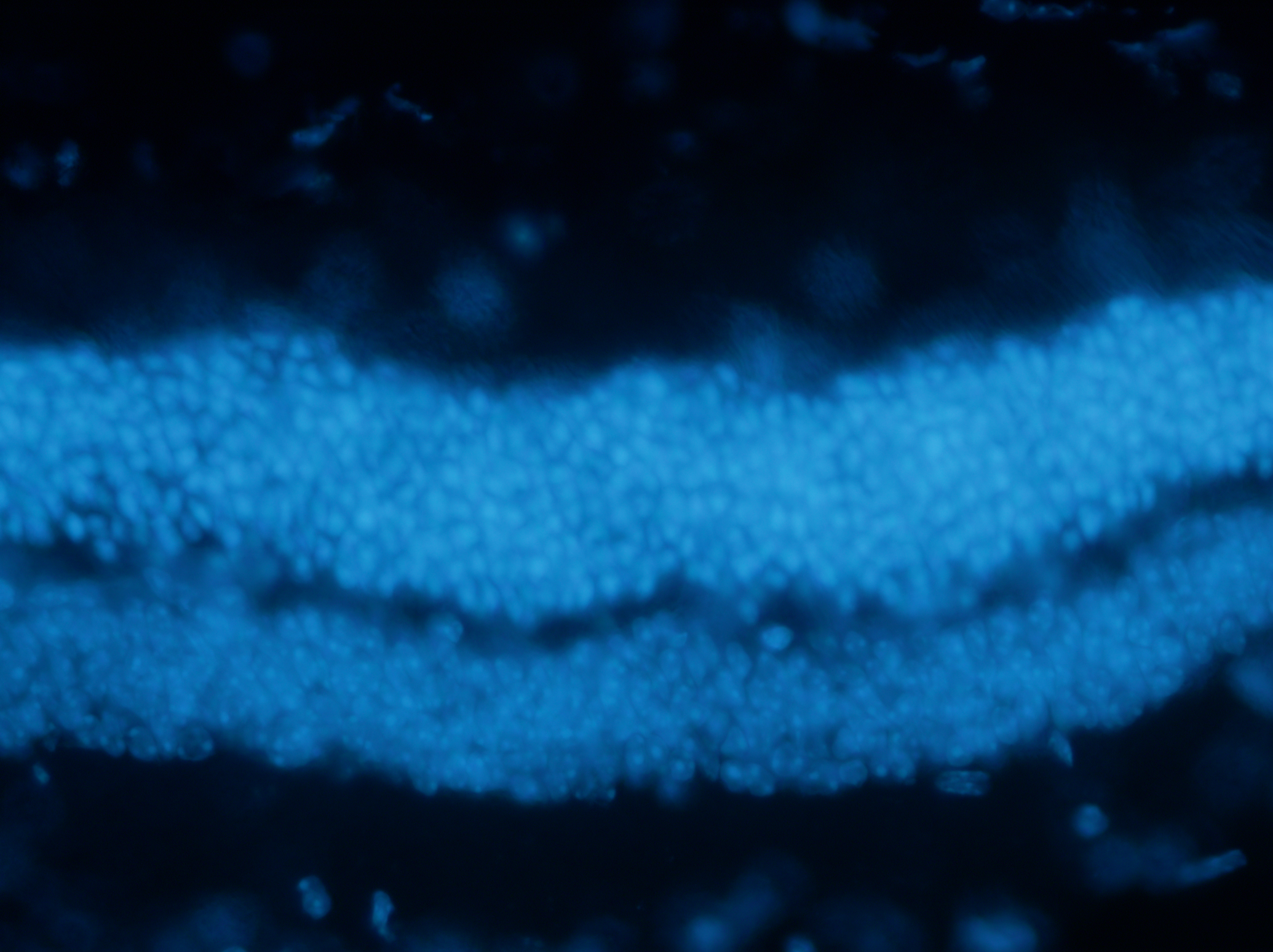

Supplement: Supplementary file 9 — Appendix Source Data [file 44321_2024_53_MOESM9_ESM.zip › Appendix/S2/1e8_DAPI.tif]

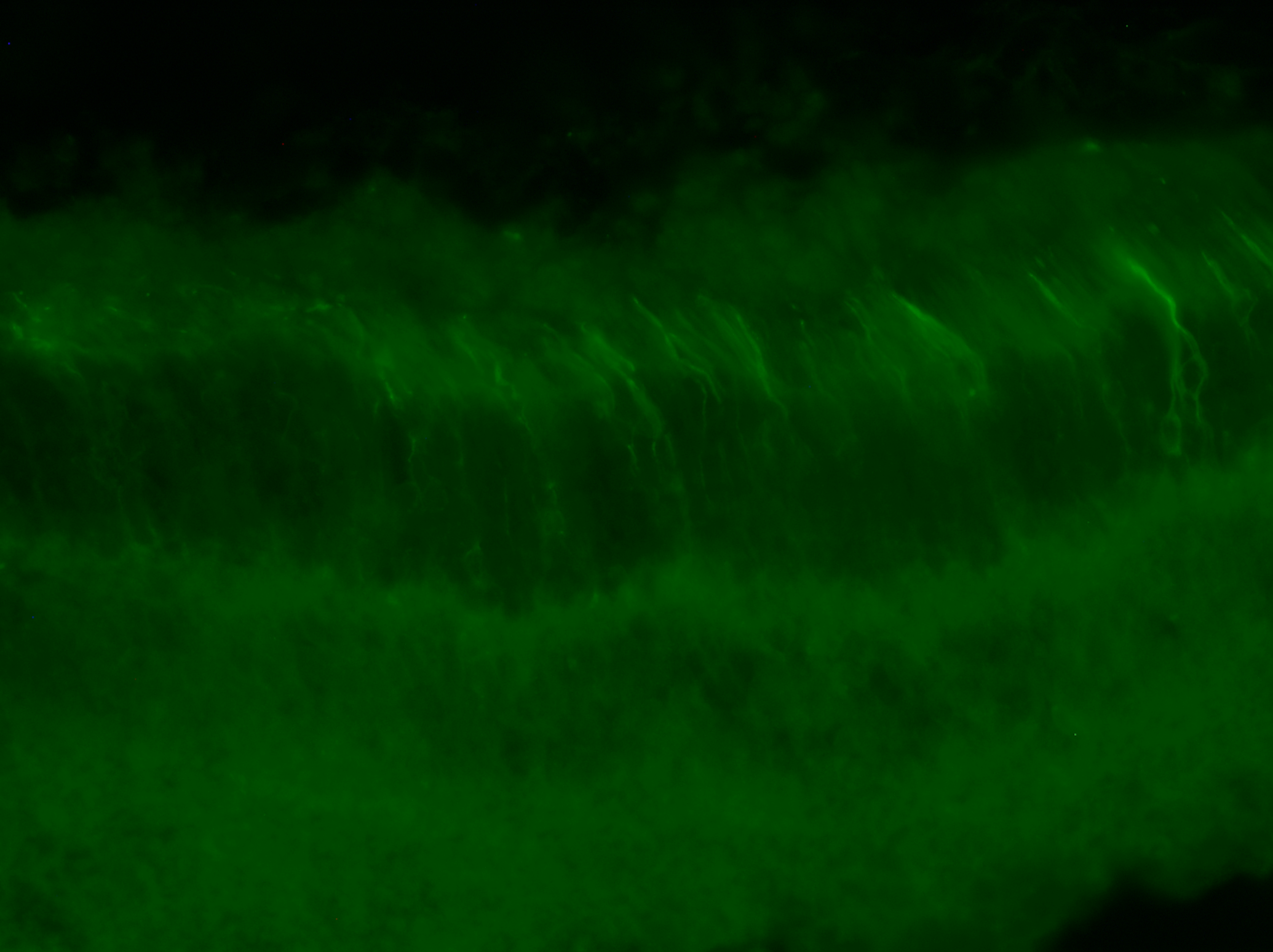

Supplement: Supplementary file 9 — Appendix Source Data [file 44321_2024_53_MOESM9_ESM.zip › Appendix/S2/1e8_FAM161A.tif]

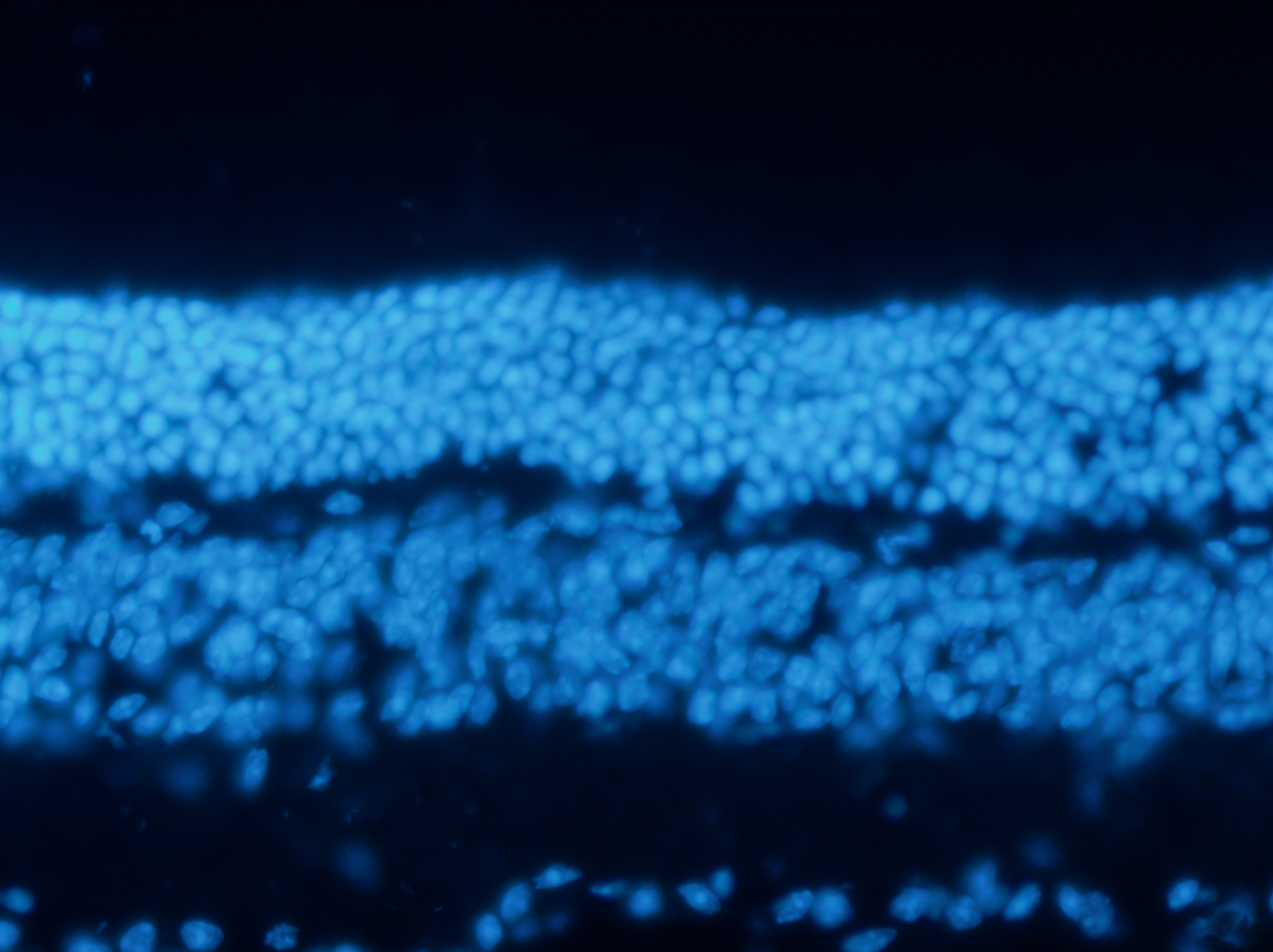

Supplement: Supplementary file 9 — Appendix Source Data [file 44321_2024_53_MOESM9_ESM.zip › Appendix/S3/HL_DAPI.tif]

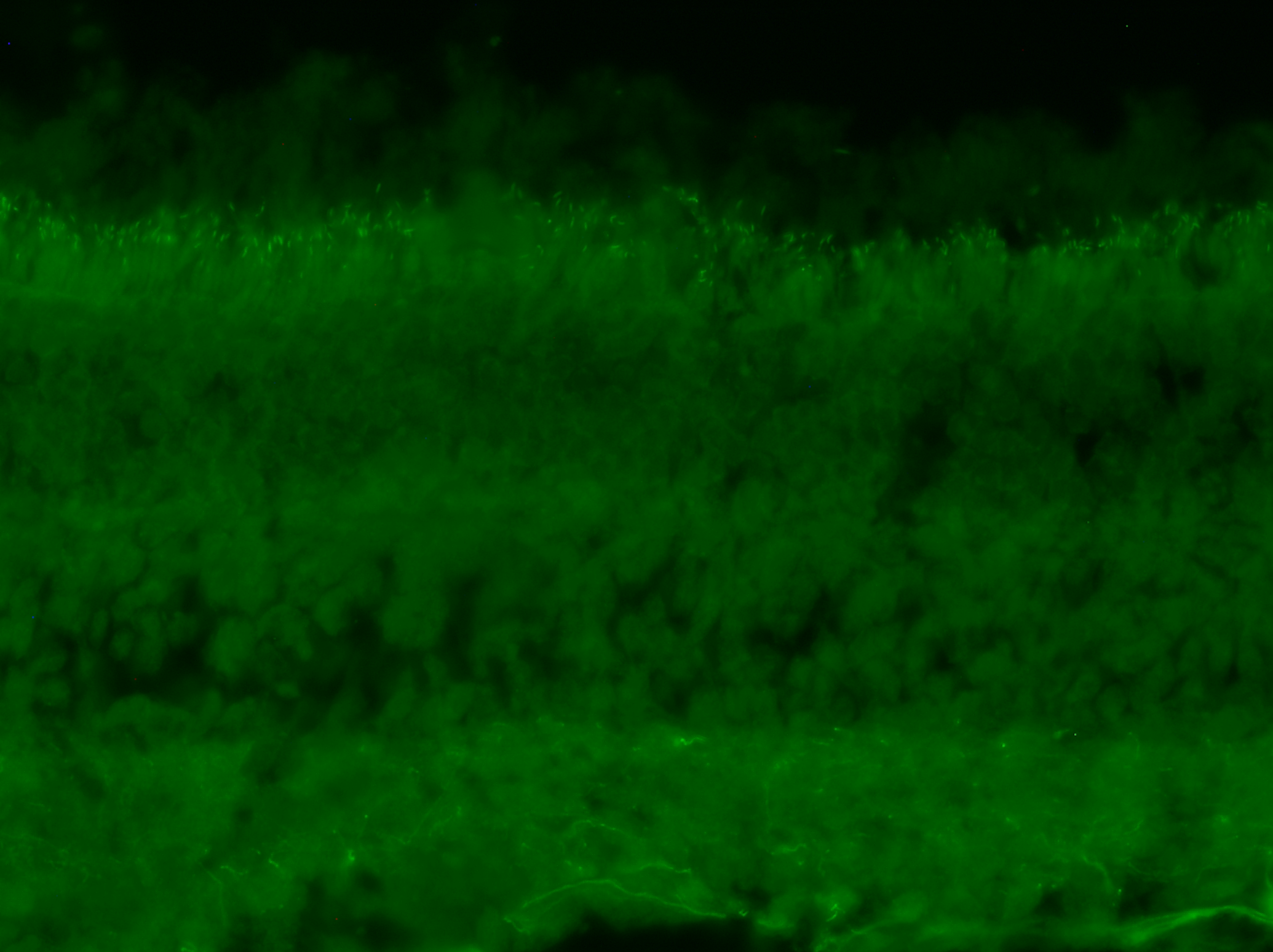

Supplement: Supplementary file 9 — Appendix Source Data [file 44321_2024_53_MOESM9_ESM.zip › Appendix/S3/HL_FAM161A.tif]

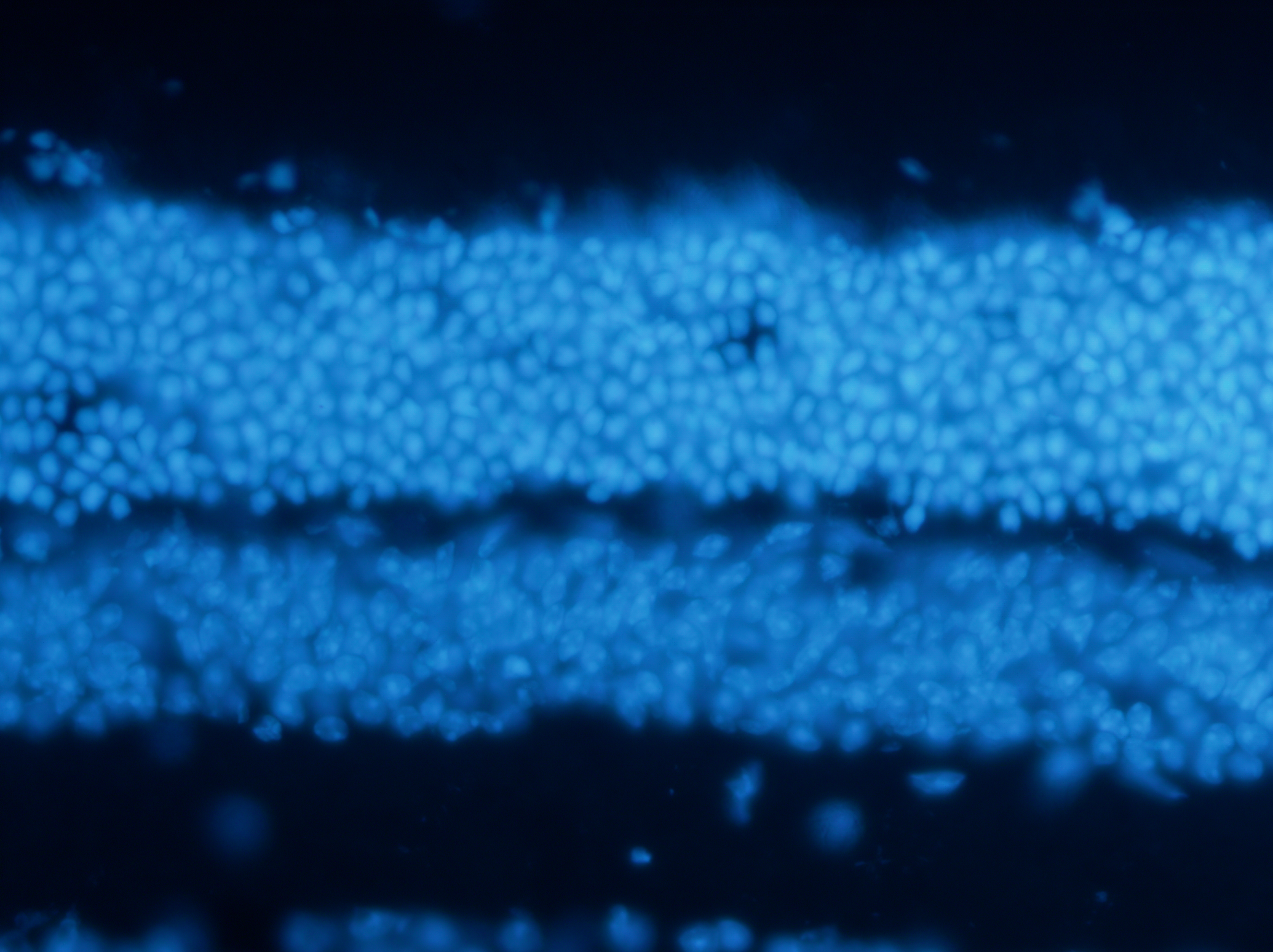

Supplement: Supplementary file 9 — Appendix Source Data [file 44321_2024_53_MOESM9_ESM.zip › Appendix/S3/HL+HS_DAPI.tif]

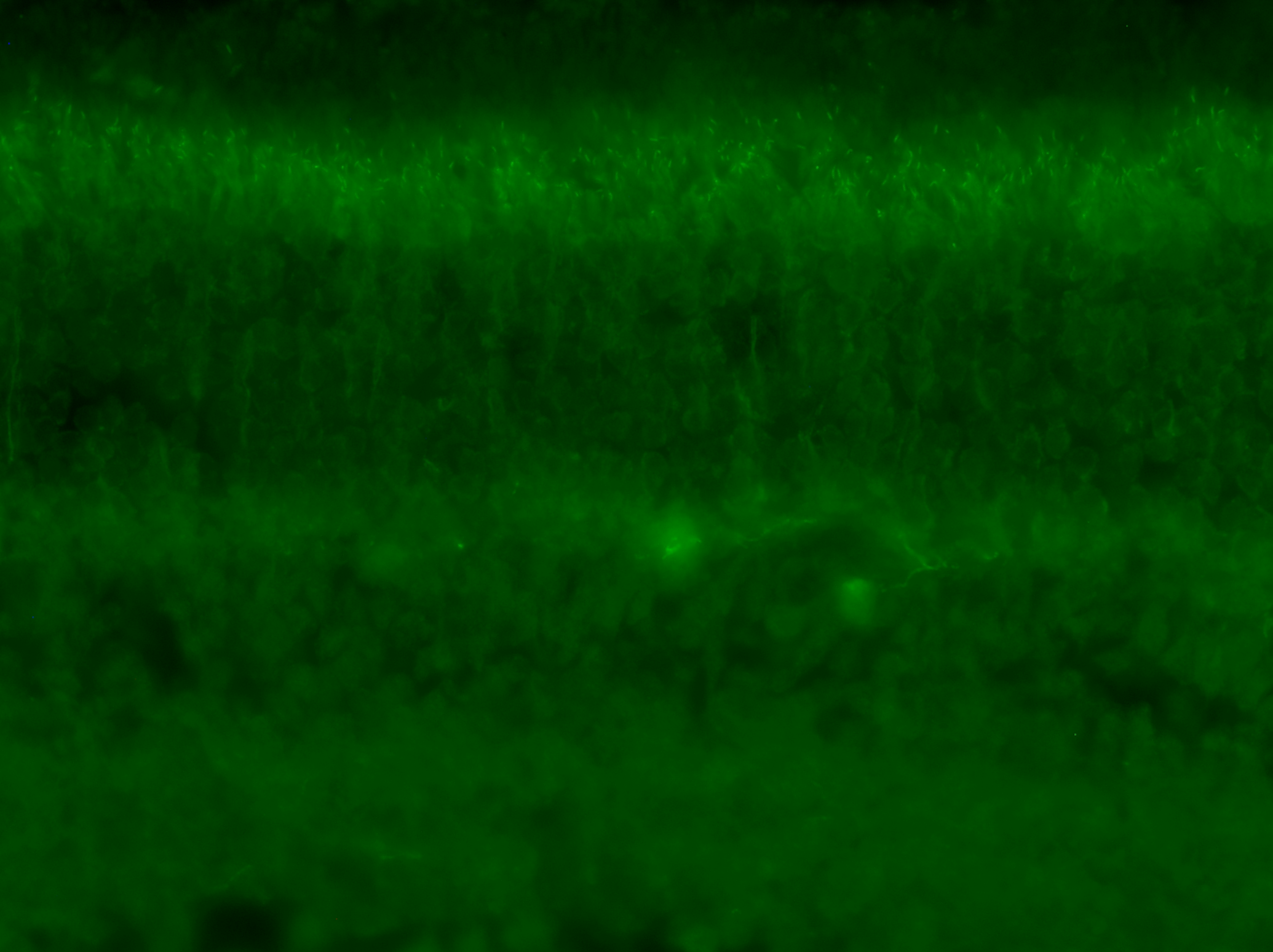

Supplement: Supplementary file 9 — Appendix Source Data [file 44321_2024_53_MOESM9_ESM.zip › Appendix/S3/HL+HS_FAM161A.tif]

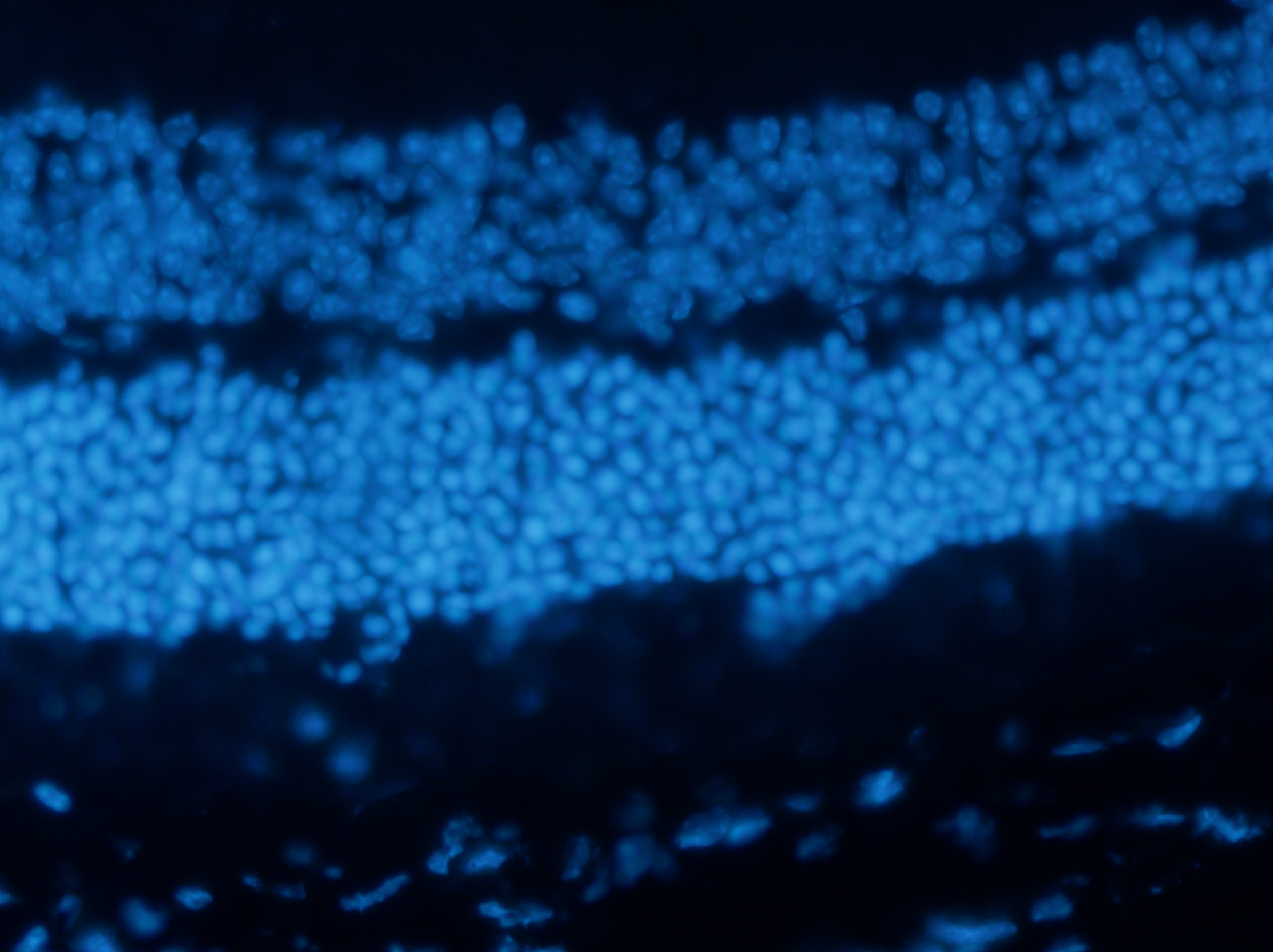

Supplement: Supplementary file 9 — Appendix Source Data [file 44321_2024_53_MOESM9_ESM.zip › Appendix/S3/HS_DAPI.tif]

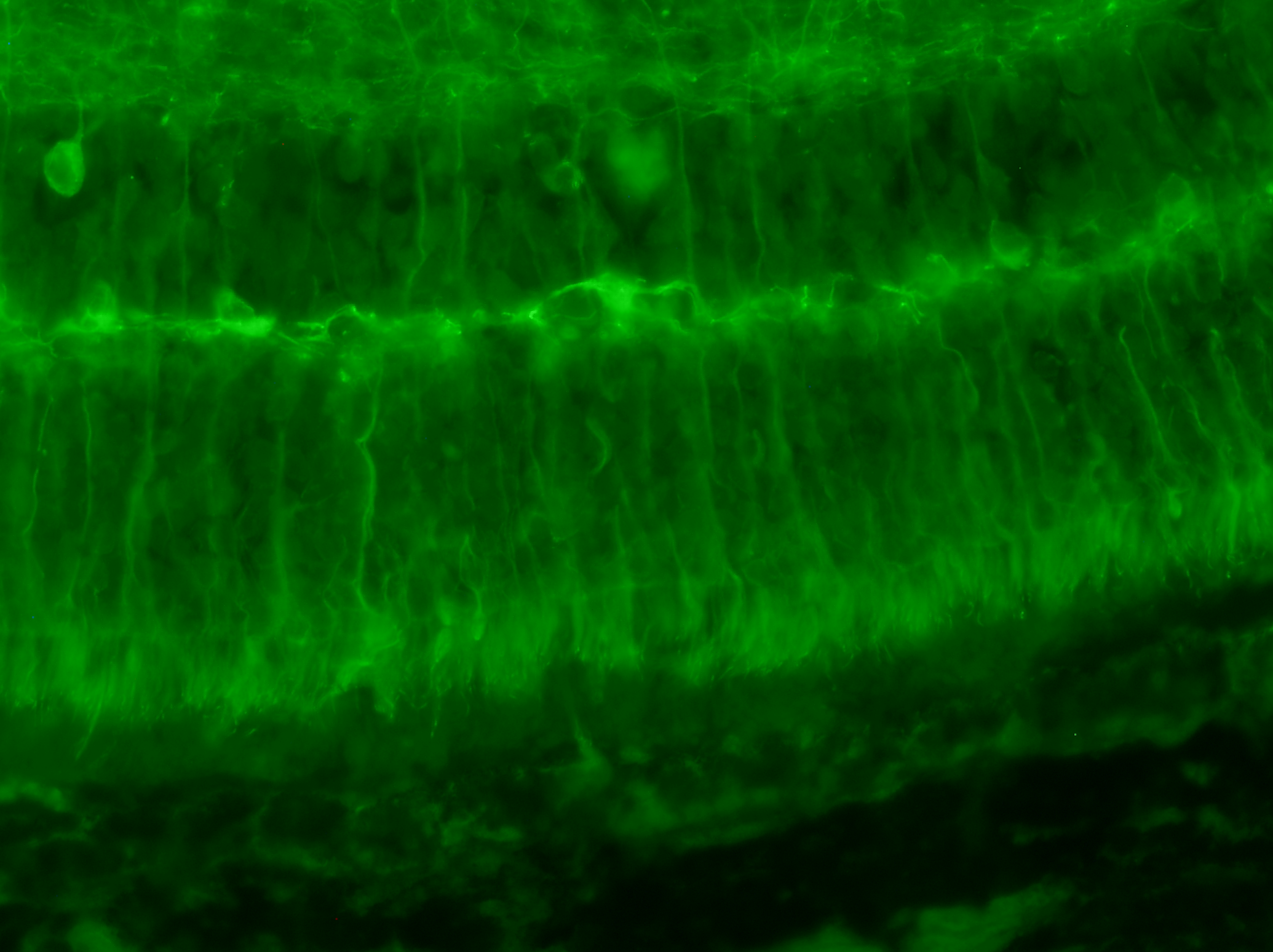

Supplement: Supplementary file 9 — Appendix Source Data [file 44321_2024_53_MOESM9_ESM.zip › Appendix/S3/HS_FAM161A.tif]

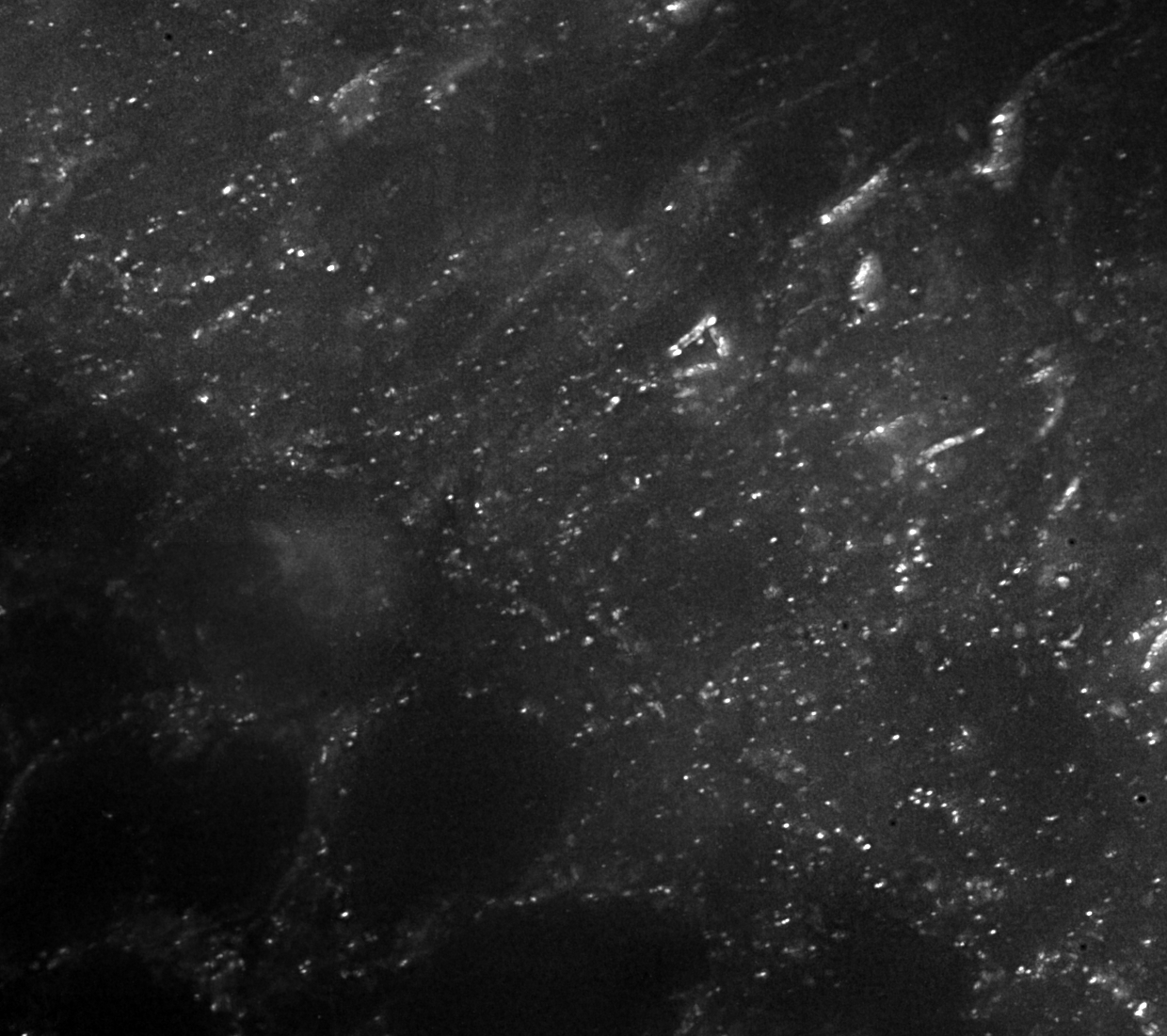

Supplement: Supplementary file 9 — Appendix Source Data [file 44321_2024_53_MOESM9_ESM.zip › Appendix/S4/HL fam161a tubulin.tif]

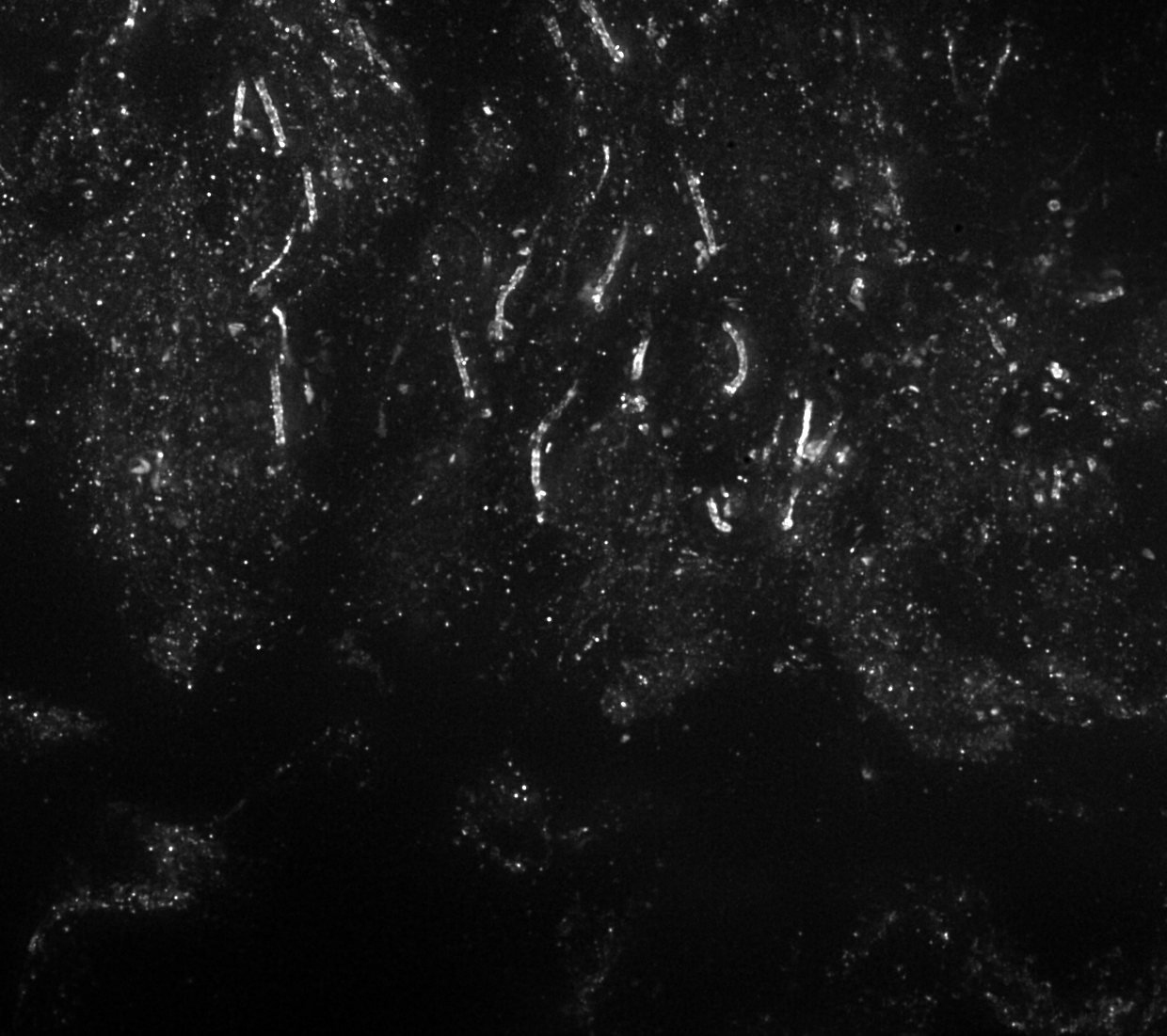

Supplement: Supplementary file 9 — Appendix Source Data [file 44321_2024_53_MOESM9_ESM.zip › Appendix/S4/HS + HL fam161a tubulin.tif]

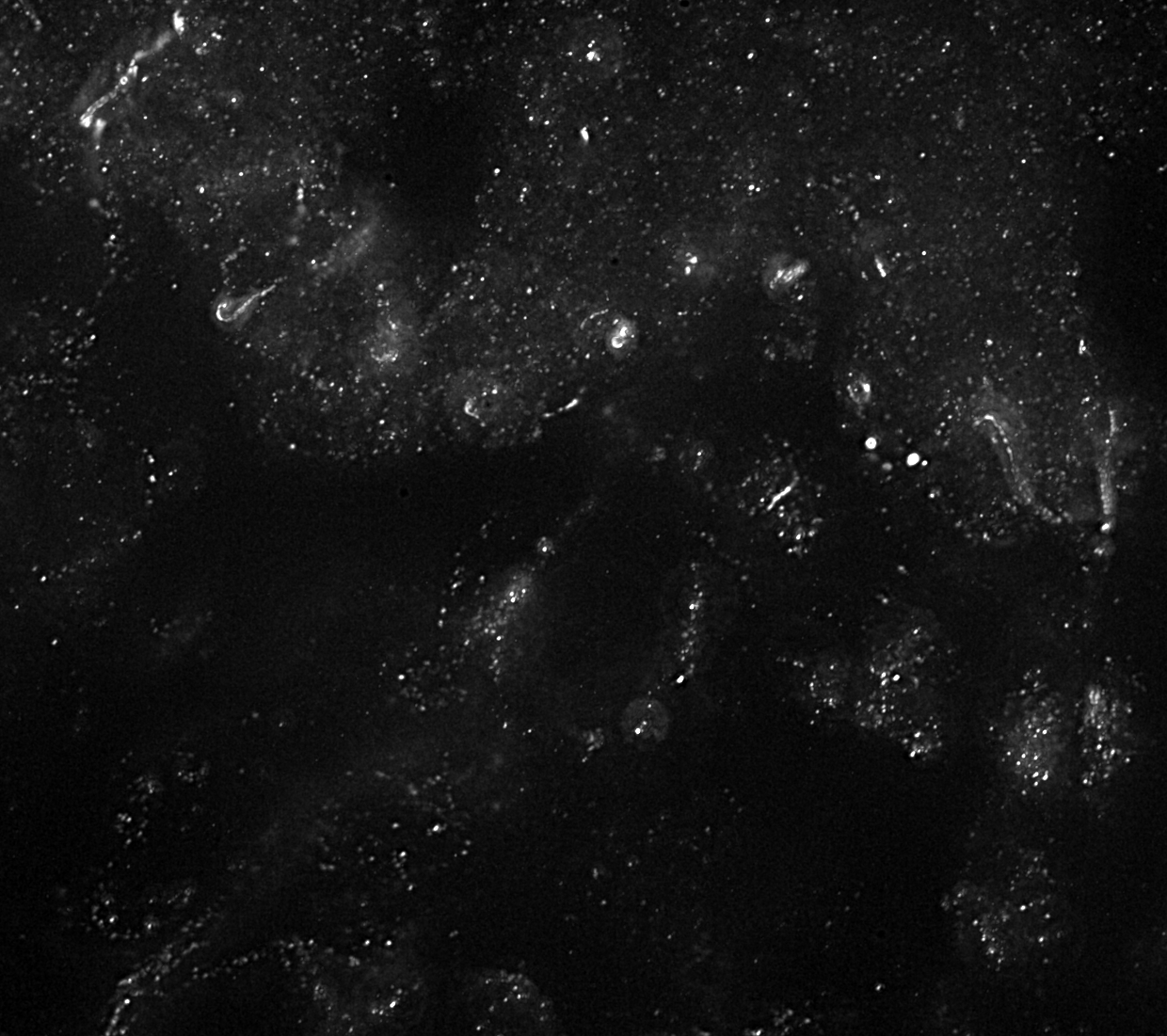

Supplement: Supplementary file 9 — Appendix Source Data [file 44321_2024_53_MOESM9_ESM.zip › Appendix/S4/HS fam161a tubulin.tif]

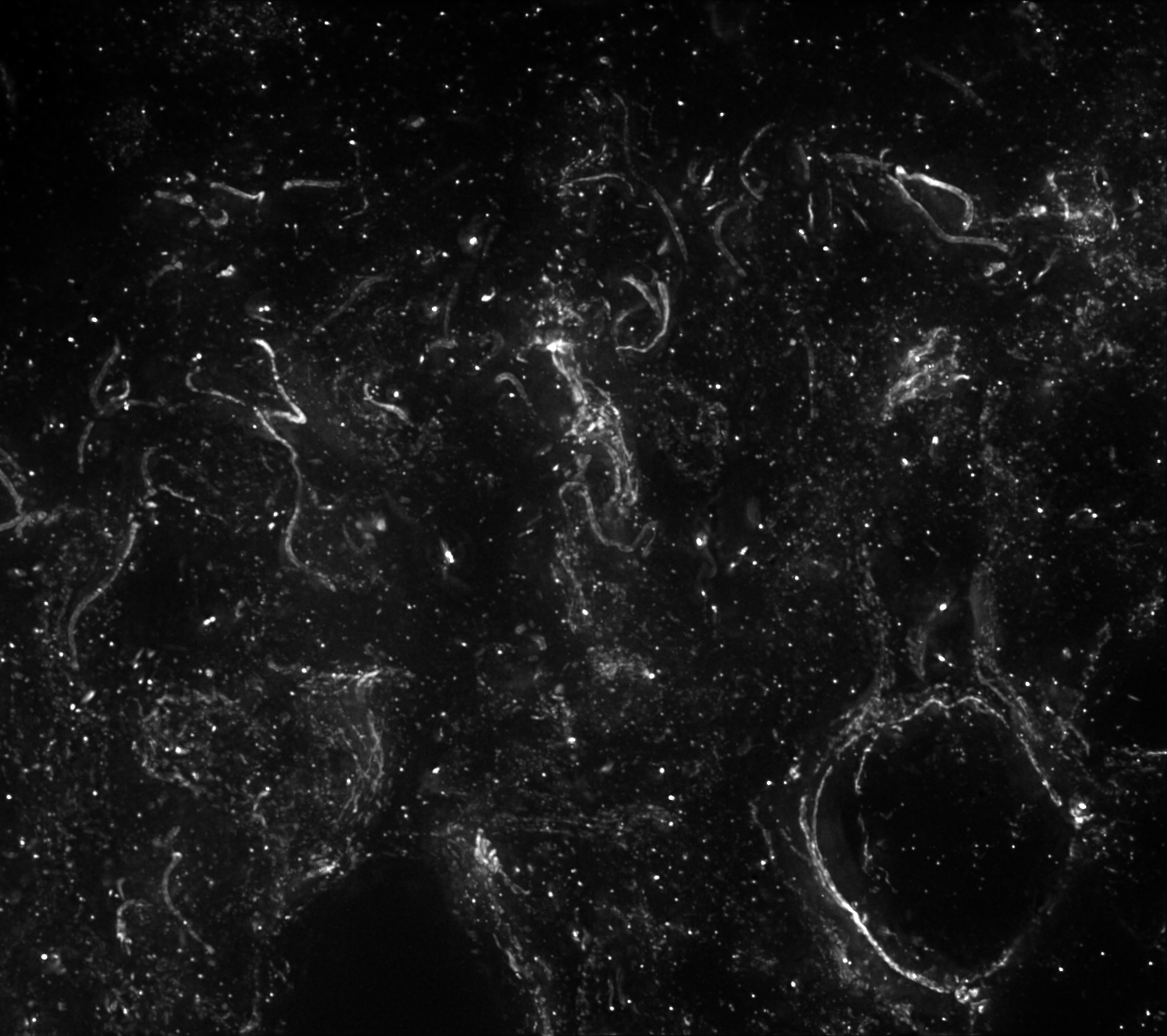

Supplement: Supplementary file 9 — Appendix Source Data [file 44321_2024_53_MOESM9_ESM.zip › Appendix/S4/IRBP fam161a tubulin.tif]

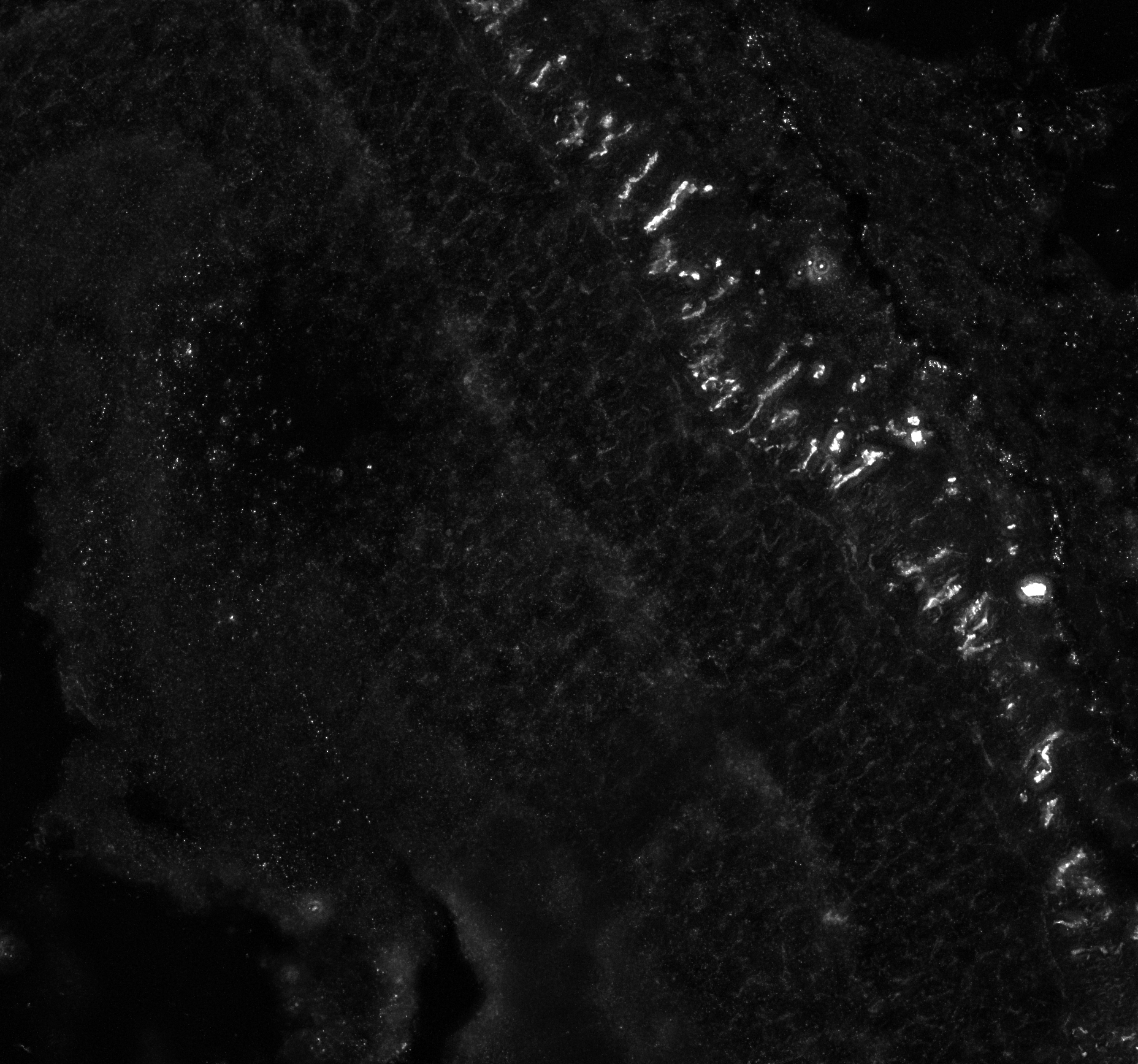

Supplement: Supplementary file 9 — Appendix Source Data [file 44321_2024_53_MOESM9_ESM.zip › Appendix/S5/HL fam161a opsin 488 tubulin low mag.tif]

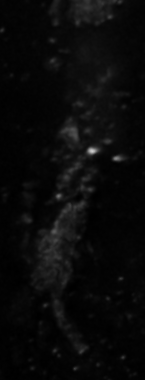

Supplement: Supplementary file 9 — Appendix Source Data [file 44321_2024_53_MOESM9_ESM.zip › Appendix/S5/HL fam161a opsin 488 tubulin.tif]

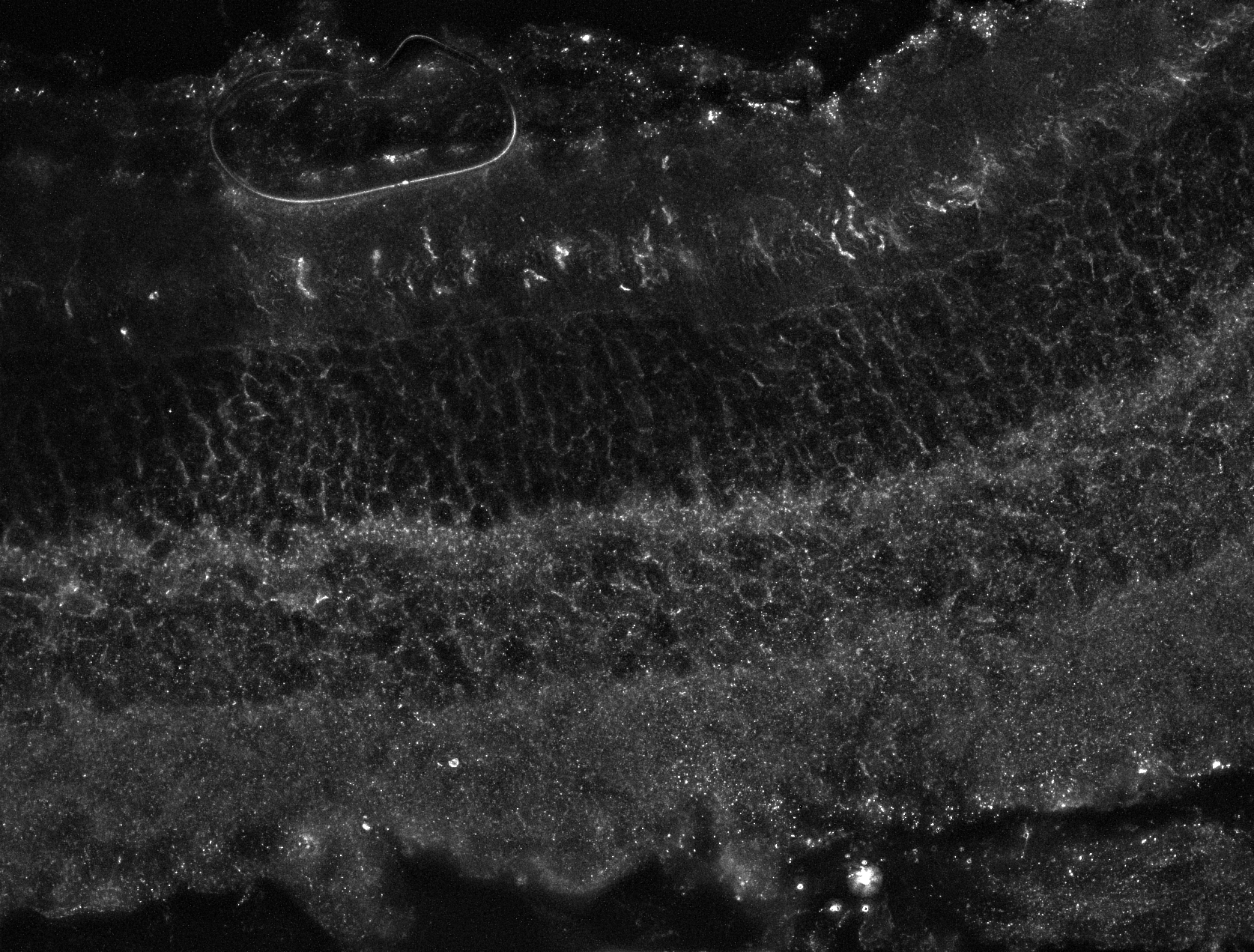

Supplement: Supplementary file 9 — Appendix Source Data [file 44321_2024_53_MOESM9_ESM.zip › Appendix/S5/HS + HL Fam161a opsin 488 tubulin low mag.tif]

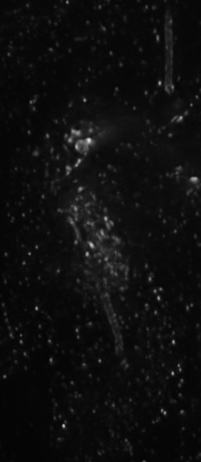

Supplement: Supplementary file 9 — Appendix Source Data [file 44321_2024_53_MOESM9_ESM.zip › Appendix/S5/HS + HL Fam161a opsin 488 tubulin.tif]

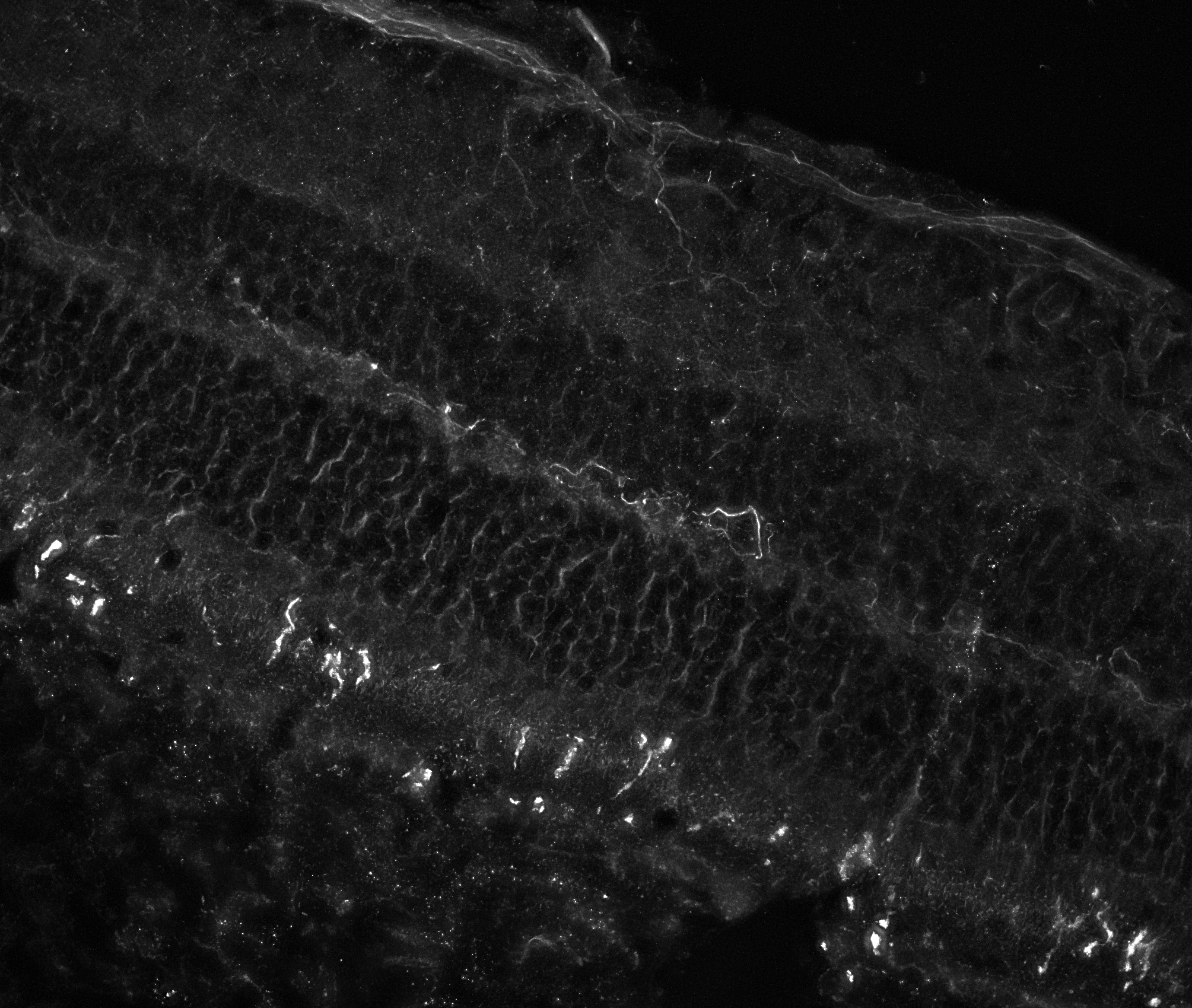

Supplement: Supplementary file 9 — Appendix Source Data [file 44321_2024_53_MOESM9_ESM.zip › Appendix/S5/HS fam161a opsin 488 tubulin low mag.tif]

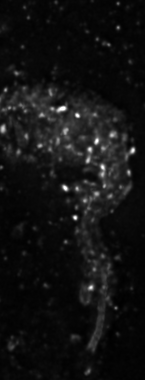

Supplement: Supplementary file 9 — Appendix Source Data [file 44321_2024_53_MOESM9_ESM.zip › Appendix/S5/HS fam161a opsin 488 tubulin.tif]
